# The *tweety* Gene Family: From Embryo to Disease

**DOI:** 10.3389/fnmol.2021.672511

**Published:** 2021-06-28

**Authors:** Rithvik R. Nalamalapu, Michelle Yue, Aaron R. Stone, Samantha Murphy, Margaret S. Saha

**Affiliations:** Department of Biology, College of William and Mary, Williamsburg, VA, United States

**Keywords:** *tweety*, *ttyh1*, *ttyh2*, *ttyh3*, embryo, neural development, TTY, chloride channel

## Abstract

The *tweety* genes encode gated chloride channels that are found in animals, plants, and even simple eukaryotes, signifying their deep evolutionary origin. In vertebrates, the *tweety* gene family is highly conserved and consists of three members—*ttyh1, ttyh2*, and* ttyh3*—that are important for the regulation of cell volume. While research has elucidated potential physiological functions of *ttyh1* in neural stem cell maintenance, proliferation, and filopodia formation during neural development, the roles of *ttyh2* and *ttyh3* are less characterized, though their expression patterns during embryonic and fetal development suggest potential roles in the development of a wide range of tissues including a role in the immune system in response to pathogen-associated molecules. Additionally, members of the *tweety* gene family have been implicated in various pathologies including cancers, particularly pediatric brain tumors, and neurodegenerative diseases such as Alzheimer’s and Parkinson’s disease. Here, we review the current state of research using information from published articles and open-source databases on the *tweety* gene family with regard to its structure, evolution, expression during development and adulthood, biochemical and cellular functions, and role in human disease. We also identify promising areas for further research to advance our understanding of this important, yet still understudied, family of genes.

## Introduction

The first member of the *tweety* gene family was initially identified in *Drosophila melanogaster* as a transcriptional unit in the *flightless* locus and was therefore named after a cartoon character that lacked the ability to fly (Campbell et al., 1993). Present in most invertebrates as a single gene or one with paralogs, the *tweety* genes encode chloride channels that are also highly conserved in most vertebrates where duplication events resulted in three distinct members, *ttyh1–3*, each with unique expression patterns (Campbell et al., 2000; Suzuki and Mizuno, 2004; Matthews et al., 2007; Kumada et al., 2010). The role of chloride channels in general, and the tweety family in particular, have been implicated in a wide variety of cellular and physiological processes in the mature organism, including cell volume regulation, muscle and neuron excitability, neurite growth, nociception, immune cell activation, stem cell migration, and wound healing (Chen et al., 2010; Duran et al., 2010; Stefaniuk et al., 2010; Guo et al., 2016; Kim et al., 2018; Han et al., 2019, 2021; Okada, 2019; Yasko et al., 2019). However, *tweety* genes are also prominently expressed throughout embryonic development, particularly in the nervous system, suggesting an important role in embryogenesis (Chen et al., 2010; Brown et al., 2015; Halleran et al., 2015; Poroca et al., 2017; Jentsch and Pusch, 2018; Karimi et al., 2018; Kim et al., 2018; Han et al., 2019). Additionally, recent research has implicated dysfunction of *tweety* family genes in a variety of human diseases (Rae et al., 2001; Xu et al., 2006; Toiyama et al., 2007; van Dijk et al., 2012; Wiernasz et al., 2014; Taylor et al., 2016; Jung et al., 2017). While a growing body of research has begun to reveal the critical importance of *tweety* genes in embryonic development, adult physiology, and pathological conditions, many intriguing questions remain unanswered regarding their function. In this review, we discuss our current knowledge of the *tweety* gene family and also identify current gaps in our knowledge of *tweety* genes and the proteins they encode as well as promising areas for further research that will extend our knowledge of this important and ancient gene family.

## Structure and Biochemical Function of Tweety Family Proteins

### Tweety Family Protein Structure

While various organisms have different numbers of *tweety* genes based on the number of gene/genome duplication events, all Tweety proteins are anion channels that exhibit a common structure consisting of multiple transmembrane domains (Campbell et al., 2000; Rae et al., 2001; Suzuki and Mizuno, 2004; He et al., 2008b). The structure of several of the Tweety proteins, however, has historically been contested. It was initially proposed that both human TTYH1 and TTYH2 have five transmembrane domains in a 2–2–1 arrangement, also referred to as the Tweety domain, consisting of a pair of transmembrane domains near the N-terminus, another pair of transmembrane domains separated from the first pair by a hydrophilic region, and a fifth transmembrane domain at the C-terminus (Campbell et al., 2000; Rae et al., 2001; Lu et al., 2020). However, Han et al. (2019), using the topology prediction software TMHMM, proposed that the previously predicted transmembrane domain at the C-terminus of Ttyh1 is instead an intramembrane domain. They verified this by expressing a murine Ttyh1-GFP fusion protein in HEK293T cells and conducting immunocytochemistry with two separate antibodies, one that binds specifically to the N-terminus of Ttyh1 and another that binds specifically to the C-terminus GFP. They demonstrated that both the N- and C-termini were located intracellularly for Ttyh1, confirming that, topologically, this last membrane domain must be intramembrane (Han et al., 2019). Interestingly, their TMHMM results placed the N- and C-termini of Ttyh1 extracellularly, which contradicted their experimental results. However, given that the TMHMM program only determines probabilities, Han et al. concluded that the N- and C-termini were located intracellularly for Ttyh1 (Sonnhammer et al., 1998; Krogh et al., 2001; Han et al., 2019). The structure of TTYH3 has also been disputed. Using transmembrane domain prediction tools and empirical studies based on the location of glycosylated residues, it was initially proposed that TTYH3 has six transmembrane domains (Suzuki and Mizuno, 2004). However, this was questioned in a subsequent article that also used hydropathy analysis and glycosylation assays and concluded that TTYH3 has five predicted transmembrane domains (He et al., 2008b).

Among orthologs and paralogs, the transmembrane domains of Tweety proteins are relatively conserved, although significant variation exists at the proteins’ C-terminal ends. For example, the original Tweety protein discovered in *D. melanogaster* and its paralog CG3638 have an extended C-terminus consisting of 520 and 205 additional hydrophilic residues repeated in short 4–6 amino acid sequences (Campbell et al., 2000). In humans, the C-terminal region of TTYH1 is shorter than those of the two previously mentioned homologs (Suzuki and Mizuno, 2004). Interestingly, this C-terminus seems to only occur for *D. melanogaster* Tty and not for other invertebrate Tweety orthologs such as those from *Branchiostoma floridae* (amphioxus) and *Ciona intestinalis* (tunicate; NCBI Resource Coordinators, 2018). The functional importance of this C-terminal extension is not known.

Recent work has also identified additional domains required for Tweety function. In mice, Han et al. (2019) identified amino acids that are necessary for channel-pore formation in Ttyh1 and Ttyh2 through systematic 20 amino acid truncations of the proteins and subsequent measurement of anion current for each deletion mutant. After identifying potential candidates in loop 2 of the proteins, further analysis identified Arginine 165 in mouse Ttyh1 and Arginine 164 in mouse Ttyh2 as being necessary for channel-pore formation, residues also present in other mammalian Ttyh1 proteins (Han et al., 2019; Uniprot Consortium, 2019). The amino acids required for the formation of the channel pore in invertebrate and non-mammalian vertebrate Tweety proteins remain unknown.

In summary, Tweety proteins all contain the Tweety domain, the amino acids forming the characteristic 2–2–1 of hydrophobic regions. The channel pore-forming residue was found to be Arginine 165 in mouse Ttyh1 and Arginine 164 in mouse Ttyh2, but the channel pore-forming residues for non-mammalian Tweety proteins are still not known. While the transmembrane regions have been identified, the locations of the N- and C-termini for mammalian Ttyh2 and Ttyh3 and non-mammalian Tweety proteins have not been experimentally determined.

### Biochemical Function of Tweety Proteins

Although the original *Drosophila* Tweety protein was initially hypothesized to be an iron transporter due to the similarity of its transmembrane domains to those of iron transporters, later research contradicted this finding (Campbell et al., 2000; Suzuki and Mizuno, 2004). There is now agreement that *tweety* genes encode chloride channels, but the ongoing discussion still exists over the precise subtype of chloride channel (Table 1).

**Table 1:** Summary table describing what is and is not agreed upon about the roles of the Tweety homologs as maxi-anion channels and the mechanisms by which they are activated.

Protein	Maxi-anion Channel?	Calcium-regulated?	Swelling-dependent?
Ttyh1	Yes^(1,2)^/No^(3)^	No^(1,2)^	Yes^(1,2,4,5)^
Ttyh2	Yes^(1,2)^/No^(3)^	Yes^(1,2)^	Yes^(4,5)^/No^(1)^
Ttyh3	Yes^(1,2)^/No^(3)^	Yes^(1,2)^	Yes^(4,5)^/No^(1,2)^

*^1^Suzuki and Mizuno, 2004; ^2^Suzuki, 2006; ^3^Sabirov and Okada, 2009; ^4^Han et al., 2019; ^5^Woo et al., 2020*.

Using patch-clamp recording, Suzuki and Mizuno (2004) maintained that the Tweety family of proteins are gated chloride channels with maxi-anion channel properties, that is, wide-pore channels with large conductance that allow the passage of larger anions such as ATP and glutamate. However, because HEK293T cells transfected with two different TTYH1 splice variants did not show typical maxi-anion phenotypes, the Tweety proteins themselves may not serve as maxi-anion channels (as reported in Sabirov and Okada, 2009; Han et al., 2019).

While additional experimental data may be required to fully assess the identity of the Tweety homologs as maxi-anion channels, there is general agreement that TTYH2 and TTYH3 are calcium-dependent channels while TTYH1 is calcium-independent. However, all three act in a swelling-dependent manner and function as volume-regulated anion channels (VRAC; Suzuki and Mizuno, 2004; Suzuki, 2006; Han et al., 2019). These channels display I_Cl, Swell_ chloride currents that occur in response to an influx of water molecules causing cell swelling which in turn triggers an efflux of chloride ions through a VRAC_swell_ channel bringing intracellular volume to normal levels. All three murine Tweety paralogs display activity of swelling-dependent volume-regulated anion channels (VRAC_swell_) in mouse astrocyte primary culture when assessed by whole-cell patch-clamp recording and shRNA-mediated knockdown diminished current (Han et al., 2019; Supplementary Table 1). Additionally, expression of each TTYH in HEK293T and CHO-K1 cells resulted in an I_Cl, Swell_ similar to that of native astrocytes, and all three Tweety paralogs were shown to be involved in the regulated volume decrease through a VRAC current in hippocampal astrocytes (Han et al., 2019; Woo et al., 2020). Bae et al. (2019) found that TTYH1 and TTYH2 are both responsible for generating VRAC currents in the SNU-601 gastric cancer cell line after using CRISPR-Cas9 technology to delete exon 7 of the *TTYH1* gene and exons 2 and 3 of the *TTYH2* gene, which reduced VRAC currents in the resulting knockout cells. They also found that TTYH1 and TTYH2 can generate VRAC currents independently in cancer cells after observing VRAC currents in the HepG2 cell line—which expresses only *TTYH1*—and in the LoVo cancer cell line—which expresses only *TTYH2* (Bae et al., 2019).

Experiments to determine the subcellular location of Tweety proteins are consistent with its identification as a transmembrane channel. Matthews et al. (2007) localized expression to the Golgi apparatus or endoplasmic reticulum (ER) with the localization shifting to the plasma membrane and inducing filopodia formation. Another study using fractionation techniques localized Tyh1 expression to the smooth ER (Kumada et al., 2010). However, Stefaniuk et al. (2010) suggested that the localization to the ER could simply be a result of normal protein processing to the plasma membrane. Consistent with earlier work, Wiernasz et al. (2014), using double immunoflourescent staining with subcellular markers in rat hippocampal neurons, observed expression in the Golgi and ER as well as clathrin-coated vesicles, late endosomes, and lysosomes. Thus, the expression of these proteins in these organelles are consistent with post-translational processing and trafficking to the cell membrane and support its function as a transmembrane chloride channel involved in the regulation of cell volume through the creation of I_Cl, Swell_ (Table 1).

### Interacting Partners of Tweety Proteins

While there are only two studies focused specifically on proteins that directly interact with individual Tweety proteins, there are several large-scale interactome studies that shed light on putative interacting partners. In terms of Tweety-focused studies, TTYH2 has also been shown through yeast two-hybrid assay to interact with β-COP, a subunit of Coat Protein Complex I, a protein required for transport from the ER to the Golgi (Ryu et al., 2019). This was further supported through Co-IP experiments. Additionally, β-COP was shown to play a role in trafficking TTYH2 to the plasma membrane as coexpression of TTYH2 and β-COP in COS-7 cells reduced TTYH2 expression at the cell surface, likely due to its role in both retrograde and anterograde transport (Ryu et al., 2019). Additionally, TTYH2 and TTYH3 expression was shown to be regulated through ubiquitination *via* Nedd4-2, a ubiquitin ligase in HEK293 cells (He et al., 2008a).

There is additional experimental evidence suggesting interactions between TTYH3 and CDKAL1, LMAN2, DHRS9 P2RY12, LPAR1, CD70, FAM189A2, FAM134C, GPR141, TMEM206, ASGR2, CD27, PTGIR, S1PR1, SLCD6A1, TNFRSF1A, GYPB, TMEM171, IPPK, and FTR2; TTYH1 and CPSF4; and TTYH2 and MANSC1 in two separate interactome studies through high-throughput affinity purification mass-spectrometry in HEK293T cells (Huttlin et al., 2015, 2017; Szklarczyk et al., 2019). TTYH2 may also interact with CCND2, KAT2A, CDK2NB, GRM1, KDELR2, NF2, PDGFRA, ERBB2, FGFR4, and IGF1R based on a study using time-resolved fluorescence resonance energy transfer in multiple lung cancer cell lines (Ivanov et al., 2017; Li et al., 2017; Szklarczyk et al., 2019). Another study showed that TTYH2 may interact with GRB2 by using affinity purification-selected reaction monitoring mass-spectrometry in HEK293T cells (Bisson et al., 2011; Szklarczyk et al., 2019). A human protein microarray suggested that TTYH2 also interacts with IKBKG (Fenner et al., 2010; Szklarczyk et al., 2019). While the Tweety proteins have many potential interactants, the precise nature of these interactions is not fully understood; rigorous analysis of these putative binding partners of the Tweety proteins may provide additional insights into their structure and biochemical functions.

## Phylogeny and Evolution of *tweety* Family Genes

**Figure 1:**
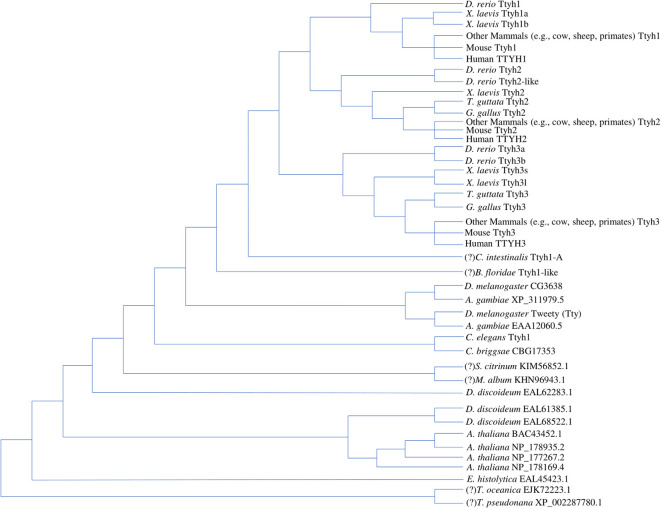
Tree diagram depicting the Tweety protein homologs identified across multiple lineages based on phylogenetic analyses by past literature. The positions of potential orthologs identified by OrthoDB are assumed based on a revised eukaryotic tree incorporating multiple phylogenomic studies, while the positions of potential amphioxus and tunicate Tweety homologs identified through BLAST searches are assumed based on the current understanding of chordate evolution (Donoghue, 2017; Kriventseva et al., 2019; Burki et al., 2020). These have been labeled with a “(?)” in front of the species name. Multiple duplication events lead to the three Tweety paralogs found in most vertebrate species, while other eukaryotes have varying numbers of homologs (Campbell et al., 2000; Matthews et al., 2007; Han et al., 2019). The diagram does not attempt any phylogenetic analyses between the homologs and is only a summary of the findings by past literature and potential homologs identified through database searches. Protein accession numbers not included in the diagram are given in the Supplementary Material (Supplementary Accession Numbers; NCBI Resource Coordinators, 2018; https://www.ncbi.nlm.nih.gov/protein).

Current work concurs that the *tweety* gene family represents an ancient and phylogenetically diverse group of genes, which are summarized in the tree diagram depicting gene duplication events that resulted in three paralogs found in most vertebrates and varying numbers of homologs found in other eukaryotes (Figure 1). In the first phylogenetic analysis of Tweety proteins, Campbell et al. (2000) compared Tweety coding regions in *Drosophila melanogaster* (fruit fly)*, Caenorhabditis elegans* (roundworm), *Mus musculus* (house mouse), and *Homo sapiens*. They identified two *tweety* paralogs in *D. melanogaster*: *tty* and *CG3638*. PANTHER, a program that annotates proteins based on homologous sequences, also identified the uncharacterized gene *CG14540* as a potential third paralog in *D. melanogaster* (Thomas et al., 2003). The human TTYH1 shares 27% identity with the *D. melanogaster* Tty and 29% with the second *D. melanogaster* Tweety paralog at the amino acid level. Campbell et al. (2000) constructed an unrooted distance neighbor-joining tree, revealing a relatively distant relationship between the two Tweety proteins in *D. melanogaster* compared to the Tweety homologs found in mice and humans, suggesting an early divergence of these genes from a duplication event (Campbell et al., 2000).

Extending the phylogenetic analysis of Tweety proteins to a broader range of eukaryotes, Matthews et al. (2007) found that the conserved Tweety domain has a deep evolutionary history. BLAST searches conducted by Matthews et al. (2007) using the *D. melanogaster* Tty protein identified three homologs in *Dictyostelium discoideum* (slime mold), one homolog in *Entamoeba histolytica* (parasitic protozoan), and four homologs in *Arabidopsis thaliana* (flowering plant; Altschul et al., 1990,^1^). The identified amino acid sequences have low homology but contain a similar arrangement of transmembrane segments to that proposed by Campbell et al. (2000).

In the alignment, Matthews et al. (2007) trimmed the N-terminal region before the first predicted transmembrane region and the C-terminal region after the last predicted transmembrane region to construct a neighbor-joining tree rooted on *E. histolytica*. The constructed tree reveals the origin of the *tweety* genes as early as in the common ancestor of animals and plants, signifying its functional importance among eukaryotes (Matthews et al., 2007). Of the three *D. discoideum* homologs identified, one is closely related to animal Tweety proteins, while the other two are closer to Tweety in plants (*A. thaliana*). This is consistent with other phylogenetic analyses of proteins in the genome of *D. discoideum*, which diverged from the ancestor of animals and fungi after the divergence of plants (Williams, 2010). One homolog was identified in *Caenorhabditis briggsae* (roundworm), which has close relatedness to the *C. elegans tweety* homolog. *Anopheles gambiae* (mosquito) was also identified to have two homologs related to *tweety*, each closely related to the paralogs found in *D. melanogaster*. Matthews et al. (2007) constructed a second tree focusing on the evolution of *tweety* in vertebrates. They note a duplication event resulting in a divergence of *ttyh3* from the ancestral gene for *ttyh1* and *ttyh2* as well as a second duplication event leading to the emergence of *ttyh1* and *ttyh2*, resulting in three *tweety* genes found in most vertebrates. The relationship between vertebrate Tweety proteins was also analyzed by Han et al. (2019) who constructed a maximum-likelihood tree that was overall consistent with the findings of Matthews et al. (Matthews et al., 2007; Han et al., 2019).

Recent advances in genomic sequencing and the publication of a diverse set of non-model organism genomes allow for a more detailed phylogenetic analysis of *tweety*-like genes and proteins. BLAST searches on publicly available genomes reveal potential Tweety proteins in the following simple organisms: five homologs in *Salpingoeca rosetta* (choanoflagellate), one homolog in *Ostreococcus tauri* (chlorophyte), and two homologs in *Sphaeroforma arctica* (unicellular eukaryote). BLAST searches also reveal potential Tweety proteins in animals: one homolog in *Amphimedon queenslandica* (sponge), *Ciona intestinalis* (tunicate), *Branchiostoma floridae* (amphioxus), and four homologs in *Petromyzon marinus* (sea lamprey; Altschul et al., 1990; NCBI Resource Coordinators, 2018). The potential amphioxus and tunicate Tweety homologs have been included in the tree diagram (Figure 1) with their positions assumed based on the current understanding of vertebrate evolution (Donoghue, 2017). According to NCBI’s conserved domain database, these Tweety proteins contain a conserved domain called the Tweety_N superfamily, which is based on their similarities with the N-terminal domain of the *D. melanogaster* Tty protein (domain accession: cl12141). A member within the superfamily is a conserved domain also named Tweety_N (cd07912), which includes a putative pore region as its feature (Lu et al., 2020,^2^). OrthoDB, a database that collects genomes from public databases such as NCBI and assesses gene homology, identified potential *tweety* orthologs in specific fungi and protists: one ortholog in *Thalassiosira oceanica* (centric diatom), *Thalassiosira pseudonana* (centric diatom), *Metarhizium album* (entomopathogenic fungi), and *Scleroderma citrinum* (common earthball), and eight orthologs in *Sphaerobolus stellatus* (shotgun fungi; Kriventseva et al., 2019,^3^). Most of these orthologs identified by OrthoDB are included in the tree diagram (Figure 1) with their positions assumed based on a revised eukaryotic tree incorporating multiple phylogenomic studies (Burki et al., 2020). Unlike the Tweety proteins in vertebrates, most Tweety homologs in other eukaryotes have not been characterized, including the three homologs in the plant *A. thaliana*, which are annotated in NCBI as plasma membrane fusion protein (protein accession: NP_178935.2), envelope glycoprotein B (NP_178169.4), and transmembrane protein (NP_177267.2; NCBI Resource Coordinators, 2018,^4^).

Current research reveals multiple independent gene duplication events in vertebrates giving rise to numerous paralogous *tweety* genes. *Xenopus laevis* (African clawed frog), an allotetraploid, has homeologous pairs for *ttyh1* (*ttyh1.s*, encoding Ttyh1b, and *ttyh1.l*, encoding Ttyh1a) and *ttyh3 (ttyh3.s* and *ttyh3.l*), unlike its closely related species, *Xenopus tropicalis* (western clawed frog), which is a diploid and lacks these pairs. The homeologous pair for *ttyh2.s* (encoding Ttyh2) could not be identified and is most likely lost in *X. laevis*. The *ttyh3.l* gene in *X. laevis* encodes a notably shorter protein compared to *ttyh3.s* and other vertebrate Tweety proteins, suggesting the possibility of a specialized function (Karimi et al., 2018). *Danio rerio* (zebrafish), *Tetraodon nigroviridis* (green spotted puffer), and *Takifugu rubripes* (tiger puffer) have paralogous pairs for *ttyh2* (*ttyh2* and *ttyh2-like*, also referred to as *ttyh2l*) and *ttyh3* (*ttyh3a* and *ttyh3b*), which is consistent with the findings of Matthews et al. (2007) and with duplication events noted in fish. Specific subfamilies within teleost fish have considerably more *tweety* paralogs correlating to their polyploidization events, which were identified through the gene gain/loss tree of the *tweety* family by ENSEMBL (Leggatt and Iwama, 2003; Yates et al., 2020,^5^). The subfamily Cyprininae has paralogs ranging from 8 in *Cyprinus carpio* (common carp) to 15 in *Carassius auratus* (goldfish), while in the subfamily Salmoninae, *Hucho hucho* (huchen) contains 13 paralogs (Yates et al., 2020). Interestingly, only *ttyh2* and *ttyh3* could be identified in birds *Taeniopygia guttata* (zebra finch) and *Gallus gallus* (red junglefowl), suggesting the possibility that *ttyh1* was lost (Altschul et al., 1990; NCBI Resource Coordinators, 2018).

An interesting finding from BLAST searches with the *D. melanogaster* Tty protein is a 100% alignment with a portion of a hypothetical protein in *Acinetobacter baumannii*, a gram-negative bacterium (protein accession: WP_143046262.1). While only 96 amino acids in length, this bacterium’s protein sequence is identical to the *D. melanogaster* Tty sequence from position 215–310 (Altschul et al., 1990; NCBI Resource Coordinators, 2018). According to SwissProt, these positions in the Tty protein contain two transmembrane segments with a cytoplasmic domain between them followed by an extracellular domain (Uniprot Consortium, 2019,^6^). These positions also have high Tweety protein alignments in both vertebrates and other eukaryotes. Additionally, the *A. baumannii* sequence aligns with the Tweety conserved domain Tweety_N superfamily. No sequences with significant similarities were found in any other bacterial species using the BLAST search tool, suggesting the possibility of a horizontal gene transfer event in which *A. baumannii* acquired the sequence from the *D. melanogaster* genome. While the sequence could be a potential Tweety homolog, no conclusions could be made on the sequence’s origin due to the lack of experimental evidence.

In summary, the *tweety* gene family has a deep evolutionary history, extending as early as the common ancestor of animals and plants. Multiple studies have shown that *tweety* genes in vertebrates evolved through two separate gene duplication events resulting in three paralogs (with the possibility of* ttyh1* being lost in birds). They have also demonstrated that the *tweety* paralogs underwent further duplication events in *X. laevis* and fish. While a relatively clear view of the evolutionary history of *tweety* genes exists for vertebrates, the *tweety* homologs have not been extensively studied in other eukaryotes, which vary in the number of homologs. This includes non-vertebrate chordates such as tunicates and amphioxi within the Deuterostomia superphylum; the Nematoda and Arthropoda phyla within the Ecdysozoa superphylum; and other clades of Eukarya including plants, fungi, and choanoflagellates. Further analysis of the expression patterns and functions of Tweety proteins across all lineages will provide additional insight into the evolution and role of the ancestral *tweety* genes.

## Expression of *tweety* Genes During Development and Adulthood

A complete understanding of the functional role of *tweety* family genes requires a thorough analysis of mRNA and protein expression patterns throughout development and beyond. While RNA-Seq analysis has provided a wealth of data, somewhat surprisingly, there are relatively few analyses of *tweety* gene expression using detailed histological analysis to identify cell/tissue type specificity. Here we review current *tweety* expression data throughout development (Supplementary Table 2) and in the mature organism (Supplementary Table 3) using all available sources of mRNA and protein expression data including published articles as well as gene expression and organism-specific expression databases.

### Invertebrate *tweety* Gene Expression

RNA-Seq data available on FlyBase indicates expression of both* tty* and its paralog *CG3638* across embryonic, larval, and pupal development, although not in identical patterns (Thurmond et al., 2019). Tissue-specific RNA-Seq data shows both genes being consistently expressed in the CNS and imaginal discs during the larval wandering stage and the fat bodies during the pupal stage. *CG3638* is generally expressed at higher levels and, unlike *tty*, is also expressed within the digestive system during the larval wandering stage (Thurmond et al., 2019). Proteomics confirms CG3638’s—but not Tty’s—expression throughout development (Thurmond et al., 2019). It should be noted that the Fly-FISH *in situ* hybridization database did not detect *tty* expression at any developmental stage (Lécuyer et al., 2007; Wilk et al., 2016). *C. elegans’s* only *tweety* homolog *ttyh1* is expressed throughout early embryonic stage into larval development and adulthood, with demonstrated expression in the embryonic pharynx and several adult tissues (Levin et al., 2012; Hashimshony et al., 2015; Harris et al., 2020). In echinoderms, RNA-Seq data on *S. pupuratus* reports that *ttyh2* expression is absent until 18 h after fertilization, when levels increase through early gastrulation (Cameron et al., 2009; Tu et al., 2014; Kudtarkar and Cameron, 2017; Cary et al., 2018).

In adults, both *tty* and *CG3638* are expressed across several tissues in *D. melanogaster*, particularly the head; however, expression of *CG3638* is stronger than that of *tty* across most sampled adult organs and is present within the digestive tract while *tty* is not (Thurmond et al., 2019).

Although expression studies (primarily RNA-Seq) are largely restricted to model organisms, *tweety* family genes in invertebrates generally display broad temporal and spatial tissue distribution. However, given that ISH was performed only in flies, there is a need for additional tissue-specific analysis at the RNA and protein level.

### Vertebrate *tweety* Gene Expression in Early Development: Gametogenesis Through Blastula

Gamete expression data derives primarily from the tetraploid *X. laevis* and *M. musculus*. qRT-PCR on *X. laevis* eggs revealed low levels of expression of all three* tweety* genes in unfertilized eggs (Halleran et al., 2015). Mouse RNA-Seq experiments have also detected very low to moderate levels of *ttyh2* and* tthy3* expression in oocytes; however, only one study detected significant expression of *ttyh1* (Bult et al., 2019; Papatheodorou et al., 2020).

Data on *tweety* gene family expression during cleavage and blastula stages also stem largely from amphibians, with some data available from mice. In mice, at the single-cell stage, *ttyh1* expression was essentially undetectable while *tthy2* was expressed at low levels and* ttyh3* at moderate levels. By the two-cell stage, all three genes were expressed at detectable levels (Bult et al., 2019).

More extensive-expression data is available from non-mammalian vertebrates. In *X. tropicalis*, RNA-Seq data shows very low levels of *ttyh1* and *ttyh2* during cleavage stages while *ttyh3* displays a steady increase in expression, peaking at the late blastula stages (Karimi et al., 2018). In *X. laevis*, pre-gastrulation expression varies between genes. While no signal was detectable using *in situ* hybridization (ISH), qRT-PCR revealed low levels of *tweety* family gene expression through blastula stages with slightly elevated levels near the mid-blastula transition for *ttyh1* and *ttyh3*, before a subsequent drop back to basal levels (Halleran et al., 2015). According to RNA-Seq data, although *ttyh2* expression remains absent throughout blastula stages, *ttyh1.s* is expressed in fertilized eggs, before decreasing during the cleavage through blastula stages. Both homeologs of *ttyh3* increase in expression at the mid-blastula transition (Karimi et al., 2018). Similar to *Xenopus*, *D. rerio tweety* gene expression during the blastula and cleavage stages vary between genes. While *ttyh1* expression is absent during cleavage and blastula stages, *ttyh2*, *ttyh2l*, *ttyh3a*, and* ttyh3b* are expressed at low to moderate levels depending on the gene (White et al., 2017).

Although limited to a few model organisms, all studied species express one or more of the *tweety* genes in oocytes through the blastula stages, with specific temporal patterns varying among genes and species. These data, which rely almost exclusively on RNA-Seq and RT-PCR results, could benefit from histological analysis.

### Expression of Vertebrate *tweety* Genes in Later Development: Gastrulation Through Organogenesis

From gastrulation onwards, mRNA levels of all three *tweety* family genes increase significantly and show distinct expression patterns.

#### *ttyh1* Expression

During gastrulation and organogenesis, *ttyh1* expression is localized to neural tissues in most vertebrate organisms. In mice, immunohisotochemistry revealed broad expression starting at late gastrula stages with elevated levels in M-phase cells (Kumada et al., 2010). By E14, ISH and immunohistochemistry experiments detected prominent expression localized to the brain and spinal cord with continuing pronounced expression in dividing cells, presumably neural progenitors (Kumada et al., 2010). ISH experiments also detected expression in the ventricular zone of the mouse brain and forebrain during organogenesis (Abramova et al., 2005; Kawaguchi et al., 2008) in addition to strong expression throughout the nervous system (Blackshaw et al., 2004; Visel et al., 2007; Allen Institute for Brain Science, 2008a; Diez-Roux et al., 2011; Thompson et al., 2014; Bult et al., 2019). Expression was also observed outside of the nervous system in the pancreas and ear and less prominently in several other organ systems (Visel et al., 2007; Bult et al., 2019). RNA-Seq experiments in embryonic mice have corroborated the localization of *ttyh1* to the CNS through the fetal stages (Brown et al., 2015; Papatheodorou et al., 2020).

Rat RNA-seq data also localizes the highest levels of expression to the developing brain, with much lower levels of expression present in the developing testes (Papatheodorou et al., 2020). Experiments conducted in hippocampus embryonic neuronal cell cultures further confirm neuronal expression (Stefaniuk et al., 2010; Wiernasz et al., 2014). Experiments in rat pup cell cultures detect glial (Wiernasz et al., 2014) and neuronal (Matthews et al., 2007) expression, with neuronal expression being preferentially localized to the axons. Human mass-spectrometry and RNA-Seq datasets show inconsistent expression in other tissues but consistent expression in the CNS (Kim et al., 2014; Brown et al., 2015; Papatheodorou et al., 2020).

In non-mammalian vertebrates, *ttyh1* also localizes to the developing CNS during gastrulation and organogenesis. In *X. laevis*, *ttyh1* mRNA ISH signal was detectable at neurula stages, with strong expression in the midbrain and eyes by the late neurula stages. By the tailbud stages, expression extended along most of the anterior-posterior neural axis (Halleran et al., 2015). As in mice, *ttyh1* is localized to the ventricular area, a region of actively dividing cells. Both qRT-PCR and RNA-Seq data show significant increases in transcript levels throughout neurulation (Halleran et al., 2015; Karimi et al., 2018). In *D. rerio*, previously absent or low *ttyh1* expression also appears during gastrulation (White et al., 2017).

#### *ttyh2* Expression

Mouse ISH data show weak to moderate expression throughout the CNS, in a single cell, punctate distribution pattern, in addition to expression in the adrenal gland, brain vasculature, and parts of the retina (Blackshaw et al., 2004; Diez-Roux et al., 2011; Hupe et al., 2017; Bult et al., 2019). RNA-Seq mouse data shows at least low levels of *ttyh2* expression in all sampled embryonic and fetal tissues, with highest expression being in the brain or testis/ovaries depending on study and stage (Brown et al., 2015; Papatheodorou et al., 2020).

The most detailed studies of *ttyh2* expression are from non-mammalian vertebrates. In *Xenopus, a* strong ISH *ttyh2* signal was present within the ganglia V, VII, IX, and X, with signal first detectable at the hatching stage (Halleran et al., 2015). In zebrafish, ISH data detected both *ttyh2* and *ttyh2l* at segmentation onwards, although only signals for* ttyh2l* were present in early to mid-somitogenesis, with signals being detected consistently in the adaxial (muscle precursor) cells but also in the neural tube, neurons, and trigeminal placode. By late somitogenesis, *ttyh2l* expression is restricted to the cranial ganglion and optic vesicle while expression of *ttyh2* appears in the CNS before extending to the retina as development progresses. In the mid-pharyngeal stages, *ttyh2l* expression appears in the retina, where it will remain in the hatching stages, and spinal cord, where it will diminish (Thisse and Thisse, 2004; Ruzicka et al., 2019). These expression patterns are consistent with RNA-Seq data showing increased expression following gastrulation (White et al., 2017).

#### *ttyh3* Expression

Mice RNA-Seq datasets identify *ttyh3* expression from somitogenesis into fetal stages. Two of these datasets also characterize tissue localization, with both reporting significant expression in the CNS and in several other tissues (Brown et al., 2015; Papatheodorou et al., 2020). Consistent with mouse expression, rat RNA-Seq data shows expression across multiple different tissue types, particularly the brain, ovary, and testis (Papatheodorou et al., 2020). In *Xenopus laevis*, qRT-PCR data identifies peak expression during neurula and early tailbud stages followed by a subsequent decline (Halleran et al., 2015). RNA-Seq data, however, identifies an earlier peak (Karimi et al., 2018). ISH experiments detected strong *ttyh3* signals throughout the anterior nervous system by the neurula stage with signals extending to the spinal cord by tailbud stages. Transient signals in the somites were also detected. Unlike *ttyh1, ttyh3* was detected primarily in postmitotic neural cells (Halleran et al., 2015). In zebrafish, RNA-Seq data also reveals expression levels increasing after gastrulation (White et al., 2017).

In summary, expression analyses point to consistent patterns of *tweety* gene expression across all vertebrate taxa analyzed, with *ttyh2* and *ttyh3* typically demonstrating expression in a broader array of tissues compared to *ttyh1* which is localized to the central nervous system. RNA-Seq analysis remains the primary mode of study for *ttyh3* while *ttyh2* and *ttyh1* patterns incorporate more detailed histological data, creating an opportunity for future study of *ttyh3*.

### Adult Expression of Vertebrate *tweety* Genes

Although detailed histological analysis of* tweety* gene expression in the mature organism is lacking, RNA-Seq data shows that *tweety* genes are still expressed in the fully developed organism with expression patterns similar to those observed in later organogenesis stages.

#### *ttyh1* Expression

Similar to embryonic and fetal development, *ttyh1* is prominently expressed in the CNS. RNA-Seq studies in rats show a consistent pattern of maximal expression in the CNS, particularly in the visual cortex, and to a lesser extent in the testes and pituitary gland (Steen et al., 1999). RT-PCR in adult rat brains detected high levels of expression in the brain stem, cerebellum, and cerebral cortex (Morciano et al., 2009). ISH experiments confirm brain expression (Matthews et al., 2007; Morciano et al., 2009), with one finding strong mRNA signals in the olfactory bulb, cerebral cortex, and cerebellum, with less intense expression in other regions of the brain (Morciano et al., 2009). A combination of western blots and immunohistochemistry experiments have shown clear expression in rat neurons (Stefaniuk and Lukasiuk, 2010; Stefaniuk et al., 2010). One study further co-localized *ttyh1* expression to the presynaptic active zone; however, two subsequent studies have only detected “negligible” (Wiernasz et al., 2014) or “rare” (Stefaniuk et al., 2010) colocalization with synaptic/synaptic vesicle markers.

Data for murine *ttyh1* mRNA and Ttyh1 protein is consistent with rat data as the highest levels of expression were found within the nervous system and the testis (Brown et al., 2015; Schmidt et al., 2018; Papatheodorou et al., 2020; Samaras et al., 2020). ISH further confirms the presence of *ttyh1* expression throughout the brain (Lein et al., 2007). RT-PCR analysis specifically identified *ttyh1* expression in the dorsal root ganglion (Al-Jumaily et al., 2007). RNA-Seq and mass-spectrometry datasets in other mammals align with these trends, with human RNA-Seq and mass-spectrometry datasets localizing the highest expression to the CNS across multiple isoforms (Supplementary Table 3). Adult expression in non-mammalian vertebrates also continues to be restricted to the CNS, specifically the brain in both *Xenopus laevis* and *tropicalis* (Karimi et al., 2018; Papatheodorou et al., 2020).

#### *ttyh2* Expression

Expression data on *ttyh2* in mature organisms almost exclusively derives from RNA-Seq data and shows broad tissue distribution. In mice, RNA-Seq studies report prominent expression in the CNS, adrenal gland, digestive system, liver, and testes, with ISH data confirming expression in the brain and spinal cord (Lein et al., 2007; Allen Institute for Brain Science, 2008b; Brown et al., 2015; Papatheodorou et al., 2020). Mass-spectrometry data further supports the expression of multiple Ttyh2 isoforms within the brain (Schmidt et al., 2018; Samaras et al., 2020). Like *ttyh1*, RT-PCR experiments identified *ttyh2* expression in adult dorsal root ganglia (Al-Jumaily et al., 2007). A single microarray experiment notes upregulated expression of *ttyh2* within adult mice myelinating oligodendrocyte cells (Edgar et al., 2013). In rat RNA-Seq datasets, *ttyh2* expression was typically highest in the brain, adrenal gland, or liver depending on the strain and study, with an additional study recording notably higher *ttyh2* expression in the superior olivary complex relative to the hippocampus or corpus striatum (Steen et al., 1999; Nothwang et al., 2006). Human RNA-Seq datasets typically identify the highest levels of expression in the CNS (Brown et al., 2015; Papatheodorou et al., 2020). Earlier northern blot experiments also detected expression in the brain and testis, with comparatively lower levels of expression in the heart and ovary and even lower levels in leukocytes, skeletal muscle, and spleen (Rae et al., 2001). Proteomics consistently notes expression in the brain; however, depending on the experiment, expression can be comparatively higher in places such as the breast and colon muscle (Stelzer et al., 2016; Schmidt et al., 2018; Samaras et al., 2020) or bone marrow (Papatheodorou et al., 2020). RNA-Seq datasets from other mammalian species are largely consistent with rat, human, and mice datasets (Supplementary Table 3).

Non-mammalian vertebrate expression is similar to that observed in mammals. In *X. laevis*, RNA-Seq analysis identified maximal adult expression within the eyes then brain and lower levels in the intestine, spleen, kidney, liver, lung, and testes, a result consistent with that in *X. tropicalis* (Karimi et al., 2018; Papatheodorou et al., 2020). In bird species *G. gallus* and reptile species *A. carolinesis, ttyh2* expression was the highest in the brain, with similar levels of expression being detected in the spleen in one *G. gallus* experiment (Papatheodorou et al., 2020).

#### *ttyh3* Expression

Like *ttyh2*, *ttyh3* is expressed in a wide range of tissues in the mature organism. In mice, most RNA-Seq studies localized maximal expression to the CNS, which mass-spectrometry data corroborates (Schmidt et al., 2018; Papatheodorou et al., 2020; Samaras et al., 2020); however, one study localized highest expression to the adrenal. Nonetheless, all RNA-Seq studies recorded significant expression across most sampled tissues (Brown et al., 2015; Papatheodorou et al., 2020). Like *ttyh1* and* ttyh2*, ISH work identified expression in the brain, and RT-PCR identified *ttyh3* expression in the dorsal root ganglia (Al-Jumaily et al., 2007; Lein et al., 2007). Rat RNA-Seq experiments identified significant expression in the spleen, lung, and brain and detectable expression in many organs/tissues (Steen et al., 1999).

Across human RNA-Seq and mass-spectrometry datasets, significant levels of adult expression were found in the CNS, kidney, spleen, bone, platelets, or immune cells depending upon the specific study, with some mass-spectrometry datasets reporting notably narrower expression ranges than RNA-Seq datasets (Kim et al., 2014; Brown et al., 2015; Stelzer et al., 2016; Schmidt et al., 2018; Papatheodorou et al., 2020; Samaras et al., 2020). Whereas mass-spectrometry data showed adult expression of all identified TTYH3 isoforms as maximal in bone, RNA-Seq data on splicing variants localized the highest expression to the brain, although only this dataset does not include values for the bone (GTex Consortium, 2015; Schmidt et al., 2018; Samaras et al., 2020). In other primate species, RNA-Seq analysis reports a wide tissue distribution of expression that varies among species (Thierry-Mieg and Thierry-Mieg, 2006). In non-primate mammals, expression levels varied with the organism being assayed, but the highest expression was typically reported in the lung, brain, or spleen (Steen et al., 1999; Papatheodorou et al., 2020).

In non-mammalian vertebrate species *G. gallus* and *X. laevis*, expression was broad, with the highest levels of expression usually localized to either the CNS and/or spleen depending on the study (Karimi et al., 2018; Papatheodorou et al., 2020). In summary, similar to embryonic expression patterns in vertebrates, *ttyh1* expression remains restricted to the CNS while *ttyh2* and *ttyh3* are expressed in a broader array of tissues including the liver and adrenal gland for *ttyh2* and spleen, lungs, and immune cells for *ttyh3*. These data are based almost exclusively on RNA-Seq and mass spectrometry data; there is a significant need for more corroborative histological analysis.

## Physiological Functions of Tweety Proteins

While the expression patterns of the various *tweety* genes provide critical information suggestive of their possible roles in the developing and mature organism, functional experiments are required to confirm these putative roles. Despite relatively few experimental studies that attempt to assess the function of the *tweety* genes, particularly for *ttyh2* and* ttyh3*, the current body of work points to a wide range of functions for the *tweety* gene family in both embryonic development and the mature organism (Supplementary Table 1).

### Function of Ttyh1 Proteins

An early study attempting to determine the role of *ttyh1* suggested its involvement in mitosis and its critical role in development at the morula and blastula stages in mice. *ttyh1* was observed to be strongly upregulated in some mouse embryo mitotic cells, suggesting that *ttyh1* is involved in progenitor cell and stem cell mitosis (Kumada et al., 2010). Immunostaining against Ttyh1 showed elevated expression of Ttyh1 in the ER of cells during metaphase and anaphase in parasagittal sections of E7.5, E11.5, and E14.5 mouse embryos (Kumada et al., 2010). Furthermore, an engineered germline loss of function mutation in *ttyh1* in which exons 5–10 were replaced with a neomycin resistance gene cassette showed early embryonic lethality in mice embryos (Kumada et al., 2010). Localization of Ttyh1 to the ER led to the hypothesis that Ttyh1 was essential for embryonic development through a possible role in maintaining calcium homeostasis during mitosis (Kumada et al., 2010). However, there is no additional evidence supporting this claim. Another study observed the localization of Ttyh1 to the ER but hypothesized that this observation was likely due to being a site of post-translational processing rather than suggesting a novel function of the Ttyh1 protein (Stefaniuk et al., 2010). Additionally, a later article contested the observation that knocking out *ttyh1* causes embryonic lethality; they proposed that the observed phenotypes were due to the knockout of a long noncoding RNA gene *NR_033548.1* which is located within the *ttyh1* gene (Wu et al., 2019). This hypothesis was further supported after mouse embryos from a knockout mouse line, created by using CRISPR-Cas9 technology to delete exon 4 of the *ttyh1* allele, developed normally. The deletion of exon 4 in *ttyh1* resulted in a truncated protein that left the *NR-033548.1* gene intact (Wu et al., 2019). However, disruption of *ttyh1* did result in a reduction of neural stem cell stemness in the embryo; neurospheres derived from *ttyh1*-deficient mice were smaller in size and number (Wu et al., 2019).

While Ttyh1 does not appear necessary for embryonic development at the blastula stage, consistent with its very low levels of expression at this stage, there is general agreement that it plays a role in maintaining neural stem cell properties in the embryo based on its expression patterns and functional analyses. This was demonstrated by neurosphere frequency increasing after overexpressing Ttyh1 in mouse E14.5 primary neural progenitors using a retroviral vector containing the murine stem cell virus long terminal repeat which drives the overexpression of *ttyh1* (Kim et al., 2018).

Ttyh1 was shown to maintain neural stem cell properties by increasing the expression of the direct downstream targets of Notch following overexpression in neural progenitor cells. *ttyh1* increased Notch IntraCellular Domain (NICD) production through the degradation of RER1 and subsequently increased maturation of γ-secretase complexes (Kim et al., 2018). The increase in NICD production was supported by western blot data using an α-NICD antibody (Kim et al., 2018). Thus, *ttyh1* appears to maintain neural stem cell properties by increasing Notch activity (Kim et al., 2018). Using microarray analysis, another study did find, however, that *ttyh1* expression is significantly upregulated in late neurula stage *Xenopus laevis* embryos after over-expressing NICD when compared to GFP-treated embryos (Vasiliu et al., 2015). Furthermore, a second study supported the idea that *ttyh1* acts downstream of Notch as treatment of mouse neurospheres with a γ-secretase inhibitor, which downregulates Notch activity, caused a downregulation of *ttyh1* at the mRNA and protein levels as assessed by qRT-PCR and western blot data (Wu et al., 2019). Where *ttyh1* is positioned in the Notch pathway signaling—whether it is upstream or downstream of NICD activation, or whether there is an auto-regulatory loop—remains an open question.

Consistent with its role in stem cell proliferation, *ttyh1* was also strongly upregulated in newly postmitotic Müller glia, the ventricular zone of the E13 and E14 mouse brain, apical progenitor cells, the presynaptic active zone of 3- to 4-week old Wistar rats, and the ventricular zone of the human hypothalamus—all highly proliferative regions (Blackshaw et al., 2004; Abramova et al., 2005; Kawaguchi et al., 2008; Morciano et al., 2009; Okamoto et al., 2016; Zhou et al., 2020).

In the developing nervous system, *ttyh1* has also been implicated in neurite growth. An siRNA-mediated knockdown of *ttyh1* in hippocampal neurons cultured from E18-E19 Wistar rats showed increased MAP2 aggregation (Stefaniuk et al., 2010). TTYH1 also appears to play a role in filopodia formation and cell adhesion as deconvolution microscopy showed colocalization of the engineered TTYH1-GFP fusion protein with filopodia and integrin on HEK293 cells (Matthews et al., 2007). Enhanced filopodia formation was also observed in neurons that overexpressed TTYH1 further supporting this idea (Stefaniuk et al., 2010). Additionally, overexpression of *ttyh1* in a hippocampal neuron cell culture showed increased neuritogenesis (Stefaniuk et al., 2010).

In the mature nervous system, several studies suggest that* ttyh1* is involved in nociception. An RNA-Seq experiment analyzed nociceptor sensory neurons in mice with and without the production of a spinal cord injury using a vessel clip (which applies a force to the wound). RNA-Seq analysis showed a downregulation of *ttyh1* in mice with a spinal cord injury when compared to sham mice, which underwent the same procedure without the vessel clip (Yasko et al., 2019). Another study found that the knockout of *ttyh1* in mice *via* CRISPR-Cas9 led to a reduced pain response and reduced nociceptor excitability in mice. Knocking down *ttyh1* in nociceptors resulted in reduced nociception and pain hypersensitivity in mice, further supporting the idea that *ttyh1* plays a role in nociception (Han et al., 2021).

Finally, regarding a completely different role, *ttyh1* was shown to be downregulated (among other genes) in human epithelial Caco-2 cells when treated with vitamin D3 (Claro da Silva et al., 2016). However, additional work is required to determine the role of *ttyh1* in the vitamin D3 pathway. In summary, *ttyh1* appears to play a central role in neural development and maintaining neural stem cell-like properties during early neurogenesis, and it may do so by interacting with the Notch signaling pathway. *ttyh1* also appears to play a role in mitosis and nociception, although these two potential roles of *ttyh1* still need more definitive evidence to flesh out mechanistic details.

### Function of Ttyh2 Proteins

Compared to *ttyh1, ttyh2* is not as extensively studied. While expression studies suggest that *ttyh2* may play a role in neural development given its strong expression in both *Xenopus* and zebrafish cranial nerves during neural embryonic development, this putative role requires functional experimental analysis (Halleran et al., 2015; Ruzicka et al., 2019). Likewise, single-cell RNA-Seq analysis revealed relatively high expression of *ttyh2* in most of the radial glial cells and outer radial glia that were analyzed, indicating a possible role in the development of this cell type (Johnson et al., 2015).

Ttyh2 has also been shown to be involved in the formation of cell membrane extensions as Hori et al. (2019) found that overexpressing *ttyh2* in retinal pigment epithelium cells caused an increase in membrane extensions in those cells.

Other investigations have focused on the role of *ttyh2* in immune function. One study found that infection of mice with lymphocytic choriomeningitis mammarenavirus caused *ttyh2* downregulation in splenic natural killer cells when compared to those of uninfected mice (Papatheodorou et al., 2020). Another study found that treatment of H1N1-infected human monocyte-derived dendritic cells with Poly I:C, an immunostimulant used to simulate viral infection, upregulated *ttyh2* (Papatheodorou et al., 2020). Likewise, treatment of primary human macrophages from patients with ulcerative colitis cultured with heat-killed *E. coli* reported *ttyh2* upregulation (Papatheodorou et al., 2020). These studies suggest a role for *ttyh2* in the immune response, although additional functional studies are needed to confirm this. This role is, however, consistent with the strong expression of *ttyh2* in organs rich with immune cells, like the spleen. Overall, *ttyh2* may be involved in the immune response; *ttyh2* may also play a role in neural development given its strong expression in the cranial nerves and glial cells of developing embryos.

### Function of Ttyh3 Proteins

*ttyh3*, like *ttyh2*, is relatively understudied compared to *ttyh1*. *ttyh3* also likely plays a role in neural development given its strong expression in neural tissue in early embryonic neural development, but its precise role remains unknown in the absence of functional studies (Brown et al., 2015; Halleran et al., 2015; Ruzicka et al., 2019).

*ttyh3* may be involved in Schwann cell repair after nerve injury as RNA-Seq analysis of Schwann cells following injury to the sciatic nerve of mice showed an upregulation of *ttyh3* when compared to mice without nerve injury (Papatheodorou et al., 2020). However, more definitive experiments are needed to determine if *ttyh3* is involved in this process.

Like *ttyh2*, *ttyh3* also seems to play a role in the adult immune system, regulating immune cell activation in response to pathogens, specifically in response to pathogen-associated molecular patterns (PAMPs). Ttyh3 was shown to facilitate ATP release in response to PAMPs (Chen et al., 2010). Additionally, neutrophils treated with lipoteichoic acid (an agonist for Toll-like receptor 2) were shown to upregulate *ttyh3* mRNA, possibly through downregulation of has-miR-1271-5p (Yen et al., 2019). TargetScan analysis showed that *ttyh3* was a downstream target of this miRNA, however, miRTarBase analysis did not corroborate this (Yen et al., 2019). It is hypothesized that this miRNA may interact with *ttyh3* among other genes in neutrophil activation (Yen et al., 2019). However, another study found that infection of mice with lymphocytic choriomeningitis mammarenavirus caused downregulation of *ttyh3* when compared to those of uninfected mice (Papatheodorou et al., 2020).

While this could be a pathogen-specific response, the downregulation of *ttyh3* in response to a pathogen in splenic killer cells is inconsistent with the results of the other studies that have shown *ttyh3* upregulation in response to pathogen and MAMP exposure, results that call for further investigation to determine the role of *ttyh3* in the immune system. In general, *ttyh3* may be involved in regulating immune cell activation in response to PAMPs. *ttyh3* may also play a role in neural development given its strong expression in neural tissue in embryonic neural development.

### Function of *Drosophila* Tweety Proteins

*Drosophila tty* was initially discovered in the *flightless* locus in *Drosophila* along with *fli*, *dodo*, and *penguin* (Campbell et al., 1993, 2000). A study that crossed a UAS-driven inverted repeat of *tty* with a Gal4 fly strain to induce RNAi knockdown of *tty* in F1 offspring found that the resulting embryos were viable and had a wild-type phenotype that was capable of flying (Mummery-Widmer et al., 2009). Another study, using the same UAS with the muscle-specific Mef2-Gal4 driver, found that the resulting offspring were viable (Schnorrer et al., 2010). To assess which genes are involved in heat nociception, Neely et al. (2010) used the pan-neuronal elav-Gal4 driver and found that resulting offspring were viable and did not exhibit decreased avoidance to noxious heat, indicating that *tty* does not play a role in heat nociception.

A paralog of *Drosophila tty*, *CG3638*, was shown to be embryonic lethal in *Drosophila* embryos in a P-element mutagenesis assay (Bourbon et al., 2002). This was further corroborated by a study showing that flies with UAS-driven inverted repeats of the *CG3638* gene crossed with flies expressing Gal4 produced F1 offspring that were embryonic lethal prior to the pupal stage (Mummery-Widmer et al., 2009). However, another study, using the muscle specific Mef2-Gal4 driver, found that the resulting offspring were viable (Schnorrer et al., 2010). In the study assessing the roles of certain genes in heat nociception, Neely et al. (2010) used the pan-neuronal elav-Gal4 driver and found that the resulting offspring were viable and did not exhibit decreased avoidance to noxious heat, indicating that *CG3638* does not play a role in heat nociception. Another study created a transgenic *Drosophila* line in which a YFP-RAB1 fusion gene was inserted into the *CG3638* gene, and this insertion proved to be viable in embryos (Zhang et al., 2007). *CG3638* may also play a role in the stress response, as the creation of heterozygous *Drosophila* containing only one functional *CG3638* allele led to decreased starvation resistance (Harbison et al., 2004). The gene may also play a role in *Drosophila* immune response as RNAi knockdown of *CG3638* resulted in reduced phagocytosis of *C. albicans* cells in plasmatocytes (Stroschein-Stevenson et al., 2006). It is also possible that *CG3638* somehow influences organismal behavior as a homozygous knockout of *CG3638* through a P-element insertion assay resulting in male *Drosophila* displaying significantly increased levels of aggressive behavior (Edwards et al., 2009).

Even though the *tweety* gene family was identified over two decades ago, much remains unknown regarding its functions within the developing and mature organism, both in *Drosophila* and vertebrates. Despite being in the *flightless* locus in *Drosophila*, *tty* is not necessary for *Drosophila* flight. Additionally, there is some experimental evidence suggesting that the paralog of *Drosophila tty*, *CG3638*, may produce an embryonic lethal phenotype in developing embryos when knocked down. *CG3638* may also be involved in the *Drosophila* immune response. These two potential roles of *CG3638* still need more definitive evidence to understand the role of *CG3638* during development and the immune response.

## The Role of *tweety* Genes in Disease

While the role of the *tweety* gene family in cancer (particularly brain and colon cancer) is well established (Supplementary Table 4), a growing number of studies are documenting its role in neurological diseases (e.g., status epilepticus, Alzheimer’s disease, Parkinson’s disease, and amyotrophic lateral sclerosis; Supplementary Table 5) and other disorders (e.g., hypertension, cystic fibrosis, and Turner syndrome; Supplementary Table 6). Much of our knowledge about the role of *tweety* genes in disease derives from differential gene expression experiments comparing normal and pathological tissues; however, as described below, studies are increasingly attempting to investigate the role of this gene family in disease on a more mechanistic level.

### The Role of *tweety* Genes in Brain Cancer

A strong association exists between *TTYH1* and brain cancers, particularly glioblastoma. Notably, *TTYH1* shRNA knockdowns in glioblastoma cell lines caused reduced glioma invasiveness, abnormal neurite membrane protrusion morphology, decreased tumor size, and increased survival in mice (Jung et al., 2017). Additionally, linear regression revealed that Ttyh1 levels quantified and analyzed using western blot positively correlated with glioblastoma invasiveness *in vivo* (Jung et al., 2017). Furthermore, an oncogenic fusion between *TTYH3* and *BRAF* intron 1 was identified in a glioblastoma multiforme patient (Weinberg et al., 2019). This fusion protein is associated with increased MEK/ERK phosphorylation and pathway signal transduction, which is important in cell proliferation and transformation (Khokhlatchev et al., 1998; Chang and Karin, 2001; Kolch, 2005; Kyriakis and Avruch, 2012; Weinberg et al., 2019).

Additional evidence for the role of *tweety* genes in gliomas comes from the association between TTYH1 and tumor microtube formation in glioblastoma cells (Osswald et al., 2015; Jung et al., 2017). Prominent in gliomas, tumor microtubes are cell membrane protrusions extending ten to hundreds of microns long and are likely involved in intercellular communication and glioma propagation (Osswald et al., 2015; Jung et al., 2017). Immunocytochemistry showed human glioblastoma stem cells *in vitro* having punctate TTYH1 localization concentrated at the membrane and growth cone-like tips of tumor microtubes, suggesting TTYH1’s possible involvement in tumor microtube growth (Jung et al., 2017). Furthermore, glioblastomas with 1p/19q chromosome arms codeletions (associated with diminished glioma propagation and impaired tumor microtube formation and function) show *TTYH1* and *TTYH2* downregulation and *TTYH3* upregulation compared to 1p/19q non-codeleted gliomas (Osswald et al., 2015; Jung et al., 2017).

Transplant studies also corroborate this association between *tweety* and microtube formation. When glioblastoma cells grown under differentiating, serum-containing conditions were implanted into mice brains, tumor microtube formation became impaired and RNA microarray showed corresponding *ttyh1* downregulation (Jung et al., 2017). Additionally, *in vivo* two-photon microscopy in mice implanted with human glioma cells showed that TTYH1-positivity was tumor microtube count-dependent as higher TTYH1-positivity was found in glioma cells with one to two tumor microtubes compared to those with more than four (Jung et al., 2017). Cells with one to two tumor microtubes were more motile than those with more than four, suggesting TTYH1’s role in facilitating glioma propagation, potentially through tumor microtube-associated mechanisms (Jung et al., 2017). Overall, reasonably consistent correlations exist between *tweety 1* downregulation, decreased tumor microtube function, and decreased glioma propagation, suggesting *tweety 1* may facilitate glioma invasiveness, potentially through tumor microtube-associated mechanisms.

A caveat when comparing pathological to normal tissues and cells is that RNA-Seq and microarray experiments demonstrate how *TTYH1* and *TTYH2* expression patterns and cancers may be cell and tissue-type-dependent. For example, *TTYH1* and *TTYH2* were upregulated in infiltrating compared to core neoplastic cells in glioblastoma patients (Darmanis et al., 2017). *tweety 2* differential expression assays also demonstrated cell and tissue type-dependent expression—RNA microarray of mouse brain glioma-associated macrophages revealed *ttyh2* upregulation while RNA-Seq of human glioma biopsies showed *TTYH2* downregulation (Huang et al., 2014; Papatheodorou et al., 2020). However, *TTYH3* consistently demonstrated upregulation in glioma tissues (Papatheodorou et al., 2020).

*TTYH1* is also implicated in other brain cancer types. In particular, RNA-Seq of embryonal tumors with multilayered rosettes demonstrated that C19MC (the chr19q13.41 miRNA cluster) amplification was due to a *TTYH1-*C19MC fusion (Archer and Pomeroy, 2014; Kleinman et al., 2014). In embryonal brain tumors associated with *C19MC*, this fusion dysregulated and increased expression of a *DNMT3B* isoform—a DNA methyltransferase that is found specifically in embryonic brains and is associated with significant overexpression in pediatric brain tumors (Sin-Chan and Huang, 2014; Gowher and Jeltsch, 2018). In medulloblastoma tumors, microarrays demonstrate consistent *TTYH1* downregulation and one microarray demonstrated *TTYH3* upregulation (Papatheodorou et al., 2020). Other microarrays demonstrate *TTYH1* and *TTYH2* downregulation in subependymal giant cell astrocytoma samples as well as *TTYH2* downregulation and *TTYH3* upregulation in atypical teratoid rhabdoid tumors (Papatheodorou et al., 2020). Interestingly, across gliomas and brain cancers, *TTYH3* shows consistent upregulation in pathological cells and tissue. Overall, RNA-Seq and microarray experiments provide correlations between brain cancers and one or more *tweety* genes while functional gene knockdowns and microscopic image analyses suggest functional associations including *tweety 1*’s facilitation of glioma invasiveness through tumor microtube-associated mechanisms, *TTYH1-*C19MC’s role in embryonal brain tumors, and *TTYH3*-*BRAF*’s role in glioma.

### The Role of *tweety* Genes in Colon Cancer

*TTYH2* has been studied in association with colon cancer through gene expression, functional gene knockdowns, and binding assays. RT-PCR found *TTYH2* upregulation in colon cancer cell lines (Caco-2, LoVo, and DLD-1) and human colon cancer tissue, although RNA-Seq of human blood platelets from colorectal cancer patients showed *TTYH2* downregulation (Toiyama et al., 2007; Papatheodorou et al., 2020). Similar to gliomas, differential *TTYH2* expression patterns between pathological and normal cells and tissue are seemingly cell and tissue type-dependent in colon cancer. Additionally, siRNA knockdown of *TTYH2* in colon cancer-derived cell lines Caco-2 and DLD-1 showed increased cell aggregation and impaired cell proliferation, suggesting a role of *TTYH2* in tumor growth and metastasis (Toiyama et al., 2007). The studies attempting to characterize *TTYH2* in colon cancer consistently suggest *TTYH2* facilitates tumor growth and/or metastasis.

In contrast with the functional studies conducted on *TTYH2* in colon cancer, *TTYH1* and *TTYH3* have only been studied in colon cancer through gene expression assays. RNA-Seq of human blood platelets from colorectal cancer patients demonstrated *TTYH1* downregulation (Papatheodorou et al., 2020). *TTYH3* demonstrated upregulation in association with colon cancer, consistent with the *TTYH3* upregulation pattern in brain cancers (Rhodes et al., 2004; Papatheodorou et al., 2020).

### The Role of *tweety* Genes in Other Cancers

In addition to central nervous system-associated cancers and colon cancers, *tweety* is implicated in other cancers including breast, clear cell sarcoma, gastric, histiocytic sarcoma, kidney, osteosarcoma, and thyroid cancers. Expression from immunohistochemistry images and clinical outcome data in patients with gastric cancer analyzed using various databases (e.g., Human Protein Atlas, Oncomine, UALCAN, and Gene Expression Omnibus) revealed upregulated TTYH3 in gastric cancer compared to normal tissue (Rhodes et al., 2004; Saha et al., 2019,^7^). Furthermore, Kaplan-Meier analysis (modeling patient survival probability over time) demonstrated a correlation between *TTYH3* upregulation and decreased patient survival (Saha et al., 2019). These findings suggest *TTYH3* facilitates gastric cancer pathology.

Although Darweesh et al. (2014) could not validate this pattern, overall, *TTYH1* downregulation in breast cancer-associated cells and tissue is consistent across RNA-Seq and microarray experiments (Rhodes et al., 2004; Darweesh et al., 2014; Papatheodorou et al., 2020 ). A Kaplan-Meier analysis also demonstrated a positive correlation between *TTYH1* expression and life expectancy in triple-negative breast cancer patients, conferring *TTYH1* as a potential disease biomarker and suggesting that *TTYH1* may hinder breast cancer progression (Zhong et al., 2020). In contrast with *TTYH1*, *TTYH2* and *TTYH3* have only been studied in breast cancer through RNA-Seq and microarray analyses with *TTYH2* showing downregulation and *TTYH3* showing upregulation in breast cancer-associated cells and tissues (Papatheodorou et al., 2020). The upregulation of *TTYH3* in breast cancer-associated cells is consistent with the upregulation reported in brain and colon cancers.

In addition to breast cancer, *TTYH2*’s role has been investigated in osteosarcoma where RNA-Seq showed *TTYH2* upregulation in mouse osteosarcoma samples (Papatheodorou et al., 2020). Additionally, an siRNA knockdown of *TTYH2* performed on osteosarcoma cell line U2OS (which showed high *TTYH2* expression) attempted to identify possible associations between *TTYH2* expression and cancerous properties of U2OS cells (Moon et al., 2019). This siRNA knockdown resulted in impaired U2OS invasion and migration but not cell proliferation (Moon et al., 2019). It also resulted in downregulation of transcription factors SLUG and ZEB1, which regulate epithelial-to-mesenchymal transition (EMT), a phenomenon characteristic of many osteosarcoma-associated signaling pathways (Moon et al., 2019). In contrast with *TTYH2*, *TTYH1* and *TTYH3* have only been studied in osteosarcoma through microarray and RNA-Seq experiments showing *TTYH1* downregulation and *TTYH3* upregulation (Papatheodorou et al., 2020). Again, *TTYH3* upregulation in osteosarcoma tissues and cells is consistent with *TTYH3* upregulation patterns seen in other cancers. In clear cell sarcomas, RNA-Seq demonstrated *ttyh1*, *ttyh2*, and *ttyh3* upregulation suggesting *tweety* may encourage clear cell sarcoma pathology (Papatheodorou et al., 2020). An intra-chromosomal fusion between exon 12 of *TTYH3* and exon 8 of *BRAF* on chromosome 7 was also identified in a case of histiocytic sarcoma without any other known cancer driver mutation (Egan et al., 2020).

In kidney cancers, DD-PCR and RT-PCR of human renal cell carcinoma samples demonstrated* TTYH2* upregulation (Rae et al., 2001). Additionally, microarrays demonstrated *ttyh1* upregulation, *ttyh2* downregulation, and *ttyh3* upregulation in mouse secondary tumors (Papatheodorou et al., 2020). Again, *ttyh3* upregulation in kidney tumor cells is consistent with *ttyh3* expression trends in other cancers (Papatheodorou et al., 2020). Additionally, a fusion transcript of *TTYH3* and* BRAF* has been identified in kinase fusion-positive thyroid carcinomas as one of many kinase fusions (Chu et al., 2020).

Finally, microarray and RNA-Seq have demonstrated abnormal *tweety* gene expression in various other cancers. For example, *tweety 1* has shown upregulation in lung cancer (in mouse lung epithelial cells) and tongue squamous cell carcinoma as well as downregulation in esophageal squamous cell carcinoma, esophageal adenocarcinoma, hepatobiliary carcinoma (in human blood platelets), lung cancer (in human blood platelets), pancreatic cancer, and skin squamous cell carcinoma (Papatheodorou et al., 2020). Overall, *tweety 1* is more commonly downregulated than upregulated in association with cancer, but this is not consistent among all tissues.

Microarray and RNA-Seq analyses have also demonstrated *tweety 2* upregulation in esophageal squamous cell carcinoma, esophageal adenocarcinoma, lung cancer (in mouse lung cells), lymphoma, and skin squamous cell carcinoma as well as downregulation in lung cancer (in human blood platelets), ovarian cancer, and pancreatic adenocarcinoma (Rhodes et al., 2004; Papatheodorou et al., 2020). Overall, *tweety 2* seems to be more commonly downregulated than upregulated in association with cancer, although not consistently in all tissues.

Through microarray and RNA-Seq analyses, *tweety 3* has demonstrated upregulation in esophageal squamous cell carcinoma, esophageal adenocarcinoma, hepatobiliary carcinoma (in human blood platelets), liver cancer, lung cancer, melanoma, pancreatic cancer, synovial sarcoma, squamous cell carcinoma, and uterine leiomyosarcoma as well as downregulation in lung cancer (in human blood platelets) and myxosarcoma (Rhodes et al., 2004; Papatheodorou et al., 2020). Overall, this suggests *tweety 3* is more commonly upregulated than downregulated in association with cancer, consistent with *tweety 3* upregulation exhibited in previously described cancers such as glioma, brain cancers, and colon cancer.

Overall, expression and functional studies demonstrate associations between *tweety* genes and cancers and suggest that they may serve as promising biomarker candidates for disease diagnosis or prognosis. As cancers are characterized by uncontrolled proliferation of pathological cells, the pattern of *tweety 1* downregulation, *tweety 2* upregulation, and *tweety 3* upregulation may suggest differential functions among the *tweety* genes with *tweety 1* regulating cell proliferation or impeding cancerous phenotypes and *tweety 2* and *tweety 3* facilitating cell proliferation or promoting cancerous phenotypes (Supplementary Table 4).

### The Role of *tweety* Genes in Neurological Disorders

In addition to cancers, *tweety* has also been implicated in neurological disorders including status epilepticus, Alzheimer’s disease, Parkinson’s disease, and amyotrophic lateral sclerosis (Supplementary Table 5). Both microarrays and immunohistochemistry have demonstrated *ttyh1* overexpression in rat neurons during epileptogenesis and 2 weeks after epileptogenic stimulus (Lukasiuk et al., 2003; Stefaniuk and Lukasiuk, 2010; Stefaniuk et al., 2010). Additionally, *in vivo* immunostaining revealed Ttyh1 immunoreactivity in reactive astrocytes after amygdala-induced status epilepticus in mice, further suggesting *ttyh1*’s role in epileptogenesis (Wiernasz et al., 2014).

Microarrays also demonstrate brain location-dependent *TTYH1* expression patterns in Alzheimer’s disease patients as the hippocampus and entorhinal cortex samples showed *TTYH1* downregulation while superior frontal gyrus and middle temporal gyrus samples revealed *TTYH1* (and *TTYH2*) upregulation (Xu et al., 2006; Liang et al., 2007; Papatheodorou et al., 2020). Studies have not been conducted to demonstrate a functional link between *tweety* and Alzheimer’s disease; therefore, functional mutagenesis studies should be conducted to elucidate the functional relevance of *tweety* in Alzheimer’s disease.

Additionally, mass-spectrometry demonstrated TTYH1 downregulation in the locus coeruleus of Parkinson’s disease patients when compared to controls (van Dijk et al., 2012). TTYH1, along with various other proteins associated with calcium homeostasis, were identified as having differential expression in Parkinson’s disease patients, suggesting a role of calcium homeostasis (and TTYH1’s association with it) in Parkinson’s disease pathogenesis (van Dijk et al., 2012).

*TTYH1* also showed differential expression in MN1 neurons expressing a TDP-43^A315T^ mutant when compared to MN1 neurons expressing human TDP-43 WT protein, suggesting *TTYH1* as a potential target of translation for the hTDP-43A315T protein. Dysfunctional regulation of RNA-binding protein TDP is implicated in amyotrophic lateral sclerosis development, and the TDP-43^A315T^ mutation is found in a subset of amyotrophic lateral sclerosis patients, suggesting a functional association between *TTYH1* and amyotrophic lateral sclerosis through mechanisms associated with TDP-43^A315T^ (Neelagandan et al., 2019).

Additional RNA-Seq and microarray experiments have demonstrated *TTYH3* downregulation in ataxia-telangiectasia, *TTYH2* upregulation in cerebral small vessel disease, and *TTYH2* downregulation in pick disease and progressive supranuclear palsy (Papatheodorou et al., 2020). Because of limited differential expression data, the *TTYH3* downregulation pattern present in association with cancers is not easily identifiable (if present) for neurological disorders.

### The Role of *tweety* Genes in Other Disorders

Although expression patterns of *tweety* genes under pathological conditions are more frequently studied in cancers and neurological disorders, their roles in other disorders such as hypertension, cystic fibrosis, and Turner syndrome have also been explored (Supplementary Table 6). For example, genome-wide association studies conducted on African Americans show an SNP in the intronic region of *TTYH2* is associated with systolic blood pressure levels and may impact hypertension incidence (Taylor et al., 2016). Additionally, *TTYH3*’s involvement in cystic fibrosis is suggested by strong downregulation of mTTYH3 in intestines of *cftr*^tumor microtubule1Cam^ mice, although mTTYH3 is not downregulated in *cftr*^TgH(neoim)Hgu^ mice with a weaker cystic fibrosis phenotype (Braun et al., 2010). Furthermore, high-resolution array-based comparative genomic hybridization performed on 17 Turner Syndrome patients found *TTYH3* (in addition to *AMZ1* and *GNA12*) as copy number variations at 7p22.3 (Li et al., 2019). Finally, genomic microarrays identified an overlapping 0.47 Mb microdeletion containing *TTYH3* at 7p22.3p22.2 in five patients exhibiting distinct facial features (e.g., broad nasal root and prominent forehead) and varying delays in development (Yu et al., 2017).

RNA-Seq and microarray experiments also demonstrate abnormal *tweety* expression in myriad other diseases. When comparing pathological to normal tissues and cells, *TTYH1* was upregulated in human atopic dermatitis skin samples and downregulated in association with actinic keratosis, chronic pancreatitis, down syndrome, familial hemophagocytic lymphohistiocytosis, Klinefelter syndrome, nevus sebaceous of Jadassohn, and psoriasis (Papatheodorou et al., 2020).

Microarray and RNA-Seq analyses also demonstrate *TTYH2* upregulation in actinic keratosis, non-dysplastic Barrett’s esophagus, juvenile idiopathic arthritis, metopic craniosynostosis, nevus sebaceous of Jadassohn, post-traumatic osteoarthritis, sagittal craniosynostosis, non-primary Sjogren syndrome, autism, and *Streptococcus pneumoniae* infection paired with chronic obstructive pulmonary disorder as well as downregulation in Klinefelter’s syndrome, Lyme disease, sepsis, and *Streptococcus pneumoniae* infection (Papatheodorou et al., 2020). Finally, microarray and RNA-Seq studies show* TTYH3* upregulation in actinic keratosis, Barrett’s esophagus, Crohn’s disease, nevus sebaceous of Jadassohn, pulmonary sarcoidosis, *Streptococcus pneumoniae* infection paired with chronic obstructive pulmonary disorder, and ulcerative colitis as well as downregulation in influenza and *Streptococcus pneumoniae* infection (Rhodes et al., 2004; Papatheodorou et al., 2020).

Overall, the disease-specific *tweety* expression patterns confer *tweety* as promising biomarker candidates for disease diagnosis or prognosis. Additionally, similar to the differential *tweety* expression patterns identified in cancers, the patterns of *tweety 1* downregulation, *tweety 2* upregulation, and *tweety 3* upregulation seem present among these diverse diseases and disorders. This may indicate *tweety* genes serve a fundamental function in maintaining physiological normal biological conditions. As more functional gene knockout studies, mutagenesis experiments, and binding assays elucidate the functions of *tweety* genes under physiological conditions, their role in pathological conditions may also become clearer.

## Discussion

Since the discovery of the first *tweety* gene family member several decades ago, ongoing research has deepened our understanding of not only the structure, function, phylogenetic distribution, and expression of this gene family, but also its importance in normal development and physiology as well as pathological conditions. However, despite significant progress, many questions about this relatively understudied family of genes remain. This review of the current literature points to several particularly important avenues for further research on the *tweety* gene family: comprehensive and phylogenetically diverse analyses of expression patterns, additional perturbation experiments to assess function, protein structure-function analyses using physical methods, identification of the position of *tweety* genes in signaling and gene networks, and the investigation of how *tweety* gene expression is regulated in both wild type and pathological conditions.

Evident from published work, there are relatively few comprehensive *in situ* analyses of *tweety* gene expression patterns during embryogenesis or in tissues of the mature organism for virtually any species. More detailed histological analysis to document cell and tissue-type specificity is required to complement functional studies. Based on discrepancies between reported mRNA and protein expression in some instances (Rae et al., 2001; Kim et al., 2014; Brown et al., 2015; Stelzer et al., 2016; Schmidt et al., 2018; Papatheodorou et al., 2020; Samaras et al., 2020), expression analyses at both these levels are essential to document translational control. In particular, *ttyh2* and *ttyh3* have received little attention. In addition to standard ISH and immunohistochemistry, employing techniques such as CLARITY, multiplexed immunohistochemistry techniques such as Immuno-SABER, or single-molecule fluorescent *in situ* hybridization (smFISH) will provide enhanced quantitative spatial specificity and co-localization with putative regulators, an approach productively employed to examine cardiac sodium and potassium currents (Raj et al., 2008; Chung et al., 2013; Saka et al., 2019).

The use of these techniques is especially important in pathological tissues to determine the cell types in which *tweety* genes are differentially and/or inappropriately expressed. Given the demonstrated interactions of *ttyh1* with the Notch signaling pathway to maintain stem cell-like properties, the interactions between *ttyh1* and the Notch signaling pathway in neural cancers cancer stem cells could uncover how *ttyh1* may regulate maintenance and metastasis of neural cancers (Vasiliu et al., 2015; Jung et al., 2017; Kim et al., 2018; Wu et al., 2019). Analysis of subcellular localization, particularly the dynamics of localization using *in vivo* imaging, could further shed light on the role of Tweety proteins in normal and pathological conditions.

While the detailed spatial and temporal investigation in a wide array of model and non-model organisms provides essential baseline information for functional and evolutionary analysis, a full complement of knockout and overexpression experiments—at the very least in select model organisms—is essential for determining the function of the *tweety* genes. These experiments are especially important given that even in mice, there is still conflicting evidence as to whether *ttyh1* is embryonic lethal (Kumada et al., 2010; Wu et al., 2019). Particularly low-hanging fruit for further investigation is the set of mutants already available for all three* tweety* genes in zebrafish (Ruzicka et al., 2019). Combining comparative genomics with CRISPR-based perturbation studies in a larger array of non-model organisms will uncover the level of functional conservation among *tweety* orthologs. A particularly intriguing avenue for future research is the role of *ttyh2* and *ttyh3* in immune cell activation, a function that may be conserved in non-mammalian vertebrates (Chen et al., 2010; Papatheodorou et al., 2020).

With respect to structure-function analysis, questions remain regarding the basic structure number of Tweety protein transmembrane domains. While bioinformatics techniques have been the primary source for Tweety protein structure prediction and analysis, additional technologies such as NMR, X-ray crystallography, and cryo-electron microscopy could corroborate current predicted structures, particularly when combined with targeted point mutation analysis to assess functional outcomes of mutations. For example, studies employing X-ray crystallography with cryo-EM have been extremely useful in determining structure-function relationships for other ion channels (Eichel et al., 2019). While evidence refutes the role of *tweety* genes as maxi anion channels (Suzuki and Mizuno, 2004; Suzuki, 2006; Sabirov and Okada, 2009; Han et al., 2019), questions still remain about the respective roles of the three Tweety proteins in generating volume regulated currents (VSOR and VRAC), particularly regarding whether they can activate VSOR current in a swelling-independent manner *via* reactive oxygen species or GPCR signaling. Integrating structural analysis with RNAi or CRISPR knockouts and electrophysiology data will further elucidate their biochemical function. The past decade has seen an exponential increase in ‘omics’ data for model and non-model organisms. Employing these data for comparative phylogenetic analysis will also provide a deeper understanding of structure-function relationships of the *tweety* gene family from an evolutionary perspective.

The position of the* tweety* family in gene and signaling networks remains an open but important question. *ttyh1* has already been shown to be involved in the Notch signaling pathway; however, whether *ttyh1* acts upstream or downstream (or both) of Notch ICD activation is unclear given that ICD overexpression upregulates* ttyh1* expression and that *ttyh1* activates ICD (Vasiliu et al., 2015; Kim et al., 2018; Wu et al., 2019). In a *Drosophila* study attempting to find novel regulators of the Notch signaling pathway through an RNAi screen, *tty* was not identified; this, however, does not preclude it being a downstream target of Notch signaling, or its association with Notch being a vertebrate-specific phenomenon (Mummery-Widmer et al., 2009). When, and precisely how, a *tweety* gene began interacting with the Notch pathway in evolutionary history is not fully understood. Nor is it known whether *ttyh2* and *ttyh3* also play a role in the Notch signaling pathway. Searching for differential expression of *tweety* genes in existing RNA-Seq datasets in which various genes were perturbed combined with performing RNA-Seq on embryos, tissues, or cell lines or performing RNA-Seq where *tweety* genes were knocked out or overexpressed will allow investigators to determine if *tweety* genes are associated with other signaling pathways and will provide the raw material for positioning *tweety* homologs in gene networks.

Finally, how *tweety* gene expression is regulated remains largely unknown. At the transcriptional level, the regulatory elements and associated binding proteins that regulate transcription have not been empirically identified in any organism, despite intriguing data from oncogenic gene fusions suggesting that *tweety* promoters are implicated in driving tumor development. Comparative genomics of putative upstream and intronic regulatory regions and systematic mining of databases such as ENCODE combined with functional analysis using transgenic approaches can be employed to identify regions that govern the appropriate spatial and temporal expression of the *tweety* genes in phylogenetically diverse organisms (Andersson and Sandelin, 2020). On the RNA processing level, the *tweety* genes, like many ion channels, have many exons (~15) and are known to have between two and five alternatively spliced variants; however, whether these variants are differentially expressed or functionally relevant is not known (Matthews et al., 2007; Stelzer et al., 2016). Finally, at the post-translational level, while all three Tweety proteins in humans undergo post-translational processing (Stelzer et al., 2016), comprehensive studies of interacting proteins are lacking. Pull-down assays and two-hybrid screens in combination with the bioinformatic information on putative protein interactors will allow investigators to flesh out this important level of regulation.

Overall, ongoing research has opened promising avenues of investigation for this intriguing but understudied gene family. More comprehensive characterization of expression, structure-function relationships, regulatory mechanisms, and perturbation experiments will elucidate the role of *tweety* genes in development and disease. Furthermore, the observed differential expression patterns between the three *tweety* genes demonstrated across diseases can inform functional assays that attempt to characterize potentially different biological roles possessed by each particular *tweety* gene. Finally, as more differential *tweety* expression data associated with diseases become increasingly available, evaluating the *tweety* genes for their capacity to act as biomarkers or even therapeutic targets becomes an increasingly promising avenue for future investigation.

## Author Contributions

Conceptualization: MS. Review of literature: AS, MS, MY, RN, and SM. Writing: AS, MY, RN, and SM. Review and editing: AS, MS, MY, RN, and SM. Creation of Tables and Figures: AS, MY, RN, and SM. Supervision and Funding: MS. All authors contributed to the article and approved the submitted version.

## Conflict of Interest

The authors declare that the research was conducted in the absence of any commercial or financial relationships that could be construed as a potential conflict of interest.

## Footnotes

^1^https://blast.ncbi.nlm.nih.gov

^2^https://www.ncbi.nlm.nih.gov/cdd

^3^https://www.orthodb.org

^4^https://www.ncbi.nlm.nih.gov/protein

^5^https://www.ensembl.org

^6^https://www.uniprot.org

^7^http://oncomine.org
