# Supplementary material for: The tweety Gene Family: From Embryo to Disease
Source: Front Mol Neurosci. 2021 Jun 28;14:672511. doi: 10.3389/fnmol.2021.672511 (PMC8273234; doi:10.3389/fnmol.2021.672511)
Supplement: Supplementary file 1 [file Table_1.docx]

**Supplementary Material**

**Supplementary Accession Numbers: *Danio rerio* Ttyh1, XP_009290420.2; *Xenopus laevis* Ttyh1a, NP_001087415.1; *X. laevis* Ttyh1b, NP_001086174.1; Mouse Ttyh1, AAZ06803.1; Human TTYH1, AAG02580.1; *D. rerio* Ttyh2, NP_001314737.1; *D. rerio* Ttyh2-like, NP_998421.1; *X. laevis* Ttyh2, NP_001080592.1; *Taeniopygia guttata* Ttyh2, XP_032607510.1; *Gallus gallus* Ttyh2, XP_015135417.1; Mouse Ttyh2, NP_444503.2; Human TTYH2, NP_116035.5; *D. rerio* Ttyh3a, NP_001032464.1; *D. rerio* Ttyh3b, XP_009297972.1; *X. laevis* Ttyh3s, NP_001085713.1; *X. laevis* Ttyh3l, NP001084779.1; *T. guttata* Ttyh3, XP_030140829.1; *G. gallus* Ttyh3, XP_015149839.1; Mouse Ttyh3, NP_780483.2; Human TTYH3, NP_079526.1; *Ciona intestinalis* Ttyh1-A, XP_009857972.1; *Branchiostoma floridae* Ttyh1-like, XP_035695080.1; *Drosophila melanogaster* CG3638, NP_726722.1; *D. melanogaster* Tty, NP_728415.4; *Caenorhabditis elegans* Ttyh1, NP_509903.1; *Caenorhabditis briggsae* CBG17353, XP_002643977.1**

**Supplementary Table 1.** Resulting phenotypes from *ttyh* gene expression perturbations. Exp. Atlas – Expression Atlas, Papatheodorou et al., 2020

| **Perturbation** | **Cell/Tissue Type** | **Phenotype** | **Method** | **Source** |
| --- | --- | --- | --- | --- |
| Knockdown of *tty* | *Drosophila* embryos | Viable embryos | UAS-driven inverted repeat RNAi screen induced by Gal4 expression | Mummery-Widmer et al., 2009 |
| Knockdown of *tty* | *Drosophila* embryos | Viable embryos | UAS-driven inverted repeat RNAi screen induced by Mef2-Gal4 expression | Schnorrer et al., 2010 |
| Knockdown of *tty* | *Drosophila* embryos | Viable embryos, no reduction in noxious heat avoidance | UAS-driven inverted repeat RNAi screen induced by eval-Gal4 expression | Neely et al., 2010 |
| Knockout of *CG3638* | *Drosophila* embryos | Embryonic lethal | P-element mutagenesis assay | Bourbon et al., 2002 |
| Knockout of *CG3638* | *Drosophila* embryos | Viable embryos | YFP-Rab1 insertion screen | Zhang et al., 2007 |
| Knockdown of *CG3638* | *Drosophila* embryos | Embryonic lethal before pupal stage | UAS-driven inverted repeat RNAi screen induced by Gal4 expression | Mummery-Widmer et al., 2009 |
| Knockdown of *CG3638* | *Drosophila* embryos | Viable embryos | UAS-driven inverted repeat RNAi screen induced by Mef2-Gal4 expression | Schnorrer et al., 2010 |
| Knockdown of CG3638 | *Drosophila* embryos | Viable embryos, no reduction in noxious heat avoidance | UAS-driven inverted repeat RNAi screen induced by eval-Gal4 expression | Neely et al., 2010 |
| Knockout of one *CG3638* allele (heterozygote) | *Drosophila* embryos | Reduced starvation resistance | P-element mutagenesis assay | Harbison et al., 2004 |
| dsRNA-mediated knockdown of *CG3638* | *Drosophila* plasmatocytes | Reduced phagocytosis in response to *C. albicans* cells | Fluorescent microscopy | Stroschein-Stevenson et al., 2005 |
| Knockout of *CG3638* | *Drosophila* embryos | Increased aggression | P-element mutagenesis assay | Edwards et al., 2009 |
| Overexpression of β-COP | LoVo cancer cells | Reduced surface expression | Confocal microscopy | Ryu et al., 2019 |
| Overexpression of β-COP | LoVo cancer cells | Reduced channel activity | Patch clamp electrophysiology | Ryu et al., 2019 |
| Knockout of exons 5 to 10 in *ttyh1* | Mouse embryos | Embryonic lethal phenotype | Engineered germline mutation replacing exons with a neomycin-resistant gene cassette | Kumada et al., 2010 |
| Knockout of exon 4 in *ttyh1* | Mouse embryos | Viable embryos, but decreased neural stem cell stemness | CRISPR-Cas9 system | Wu et al., 2019 |
| Overexpression of *ttyh1* with a retroviral vector | Mouse E14.5 primary neural progenitors | Increased neurosphere frequency | Light microscopy | Kim et al., 2018 |
| Overexpression of *ttyh1* with a retroviral vector | Mouse E14.5 primary neural progenitors | Increased expression of Notch downstream targets | qPCR | Kim et al., 2018 |
| Overexpression of *ttyh1* with a retroviral vector | Mouse E14.5 primary neural progenitors | Increased γ-secretase activity | Luciferase reporter assay | Kim et al., 2018 |
| Treatment with γ-secretase inhibitor | Mouse neurospheres | Downregulation of *ttyh1* | qRT-PCR and western blot | Wu et al., 2019 |
| Treatment with Notch ICD | Stage 18 *Xenopus laevis* embryos | Upregulation of *ttyh1* | Microarray | Vasiliu et al., 2015 |
| Spinal cord injury | Mouse sensory neurons | Downregulation of *ttyh1* | RNA-Seq | Yasko et al., 2019 |
| Knockout of *ttyh1* with CRISPR-Cas9 | Mouse | Reduction in pain response and nociception | Mechanical stimulation | Han et al., 2020 |
| shRNA-mediated knockdown of *ttyh1* | Mouse nociceptors | Reduction in nociception and pain hypersensitivity | Mechanical stimulation | Han et al., 2020 |
| Axotomy | Mouse sciatic nerve neurons | No change in expression of *ttyh2* or *ttyh3* | RT-PCR | Al-Jumaily et al., 2007 |
| Overexpression of *ttyh1* with a plasmid vector | Rat hippocampal neuron culture | Enhanced filopodia formation | Confocal microscopy | Stefaniuk et al., 2010 |
| siRNA-mediated knockdown of *ttyh1* | Rat hippocampal neuron culture | Increased neurite growth | Confocal microscopy | Stefaniuk et al., 2010 |
| siRNA-mediated knockdown of *ttyh2* | Mouse axotomized sensory neuron culture | No change in CaCC amplitude | Whole-cell patch clamp recording | Boudes et al., 2009 |
| siRNA-mediated knockdown of *ttyh3* | Mouse axotomized sensory neuron culture | No change in CaCC amplitude | Whole-cell patch clamp recording | Boudes et al., 2009 |
| shRNA-mediated knockdown of *ttyh1* | Mouse primary hippocampal astrocyte culture | Elimination of regulated volume decrease | Intrinsic optical signal imaging | Han et al., 2019; Woo et al., 2020 |
| shRNA-mediated knockdown of *ttyh2* | Mouse primary hippocampal astrocyte culture | Elimination of regulated volume decrease | Intrinsic optical signal imaging | Han et al., 2019; Woo et al., 2020 |
| shRNA-mediated knockdown of *ttyh3* | Mouse primary hippocampal astrocyte culture | Elimination of regulated volume decrease | Intrinsic optical signal imaging | Han et al., 2019; Woo et al., 2020 |
| shRNA-mediated knockdown of *ttyh1* | Mouse primary hippocampal astrocyte culture | Reduced VRAC currents | Whole cell patch clamp recording | Han et al., 2019 |
| shRNA-mediated knockdown of *ttyh2* | Mouse primary hippocampal astrocyte culture | Reduced VRAC currents | Whole cell patch clamp recording | Han et al., 2019 |
| shRNA-mediated knockdown of *ttyh3* | Mouse primary hippocampal astrocyte culture | Reduced VRAC currents | Whole cell patch clamp recording | Han et al., 2019 |
| Overexpression of *ttyh2* | Retinal pigment epithelium cell culture | Increased membrane extensions | Confocal microscopy | Hori et al., 2019 |
| Infection with lymphocytic choriomengitis mammarenavirus | Mice splenic natural killer cells | Downregulation of *ttyh2* and *ttyh3* | RNA-Seq | Exp. Atlas |
| Treatment with Poly I:C | Human monocyte-derived dendritic cells | Upregulation of *ttyh2* | RNA-Seq | Exp. Atlas |
| Treatment with heat-killed *E. coli* | Human macrophages cultured from patients with ulcerative colitis | Upregulation of *ttyh2* | RNA-Seq | Exp. Atlas |
| Pharmacological inhibition of TTYH3 via DIDS | Human polymorphonuclear neutrophil culture | Decreased ATP release | ATP bioluminescence assay | Chen et al., 2010 |
| siRNA-mediated knockdown of TTYH3 | Human polymorphonuclear neutrophil culture | Decreased ATP release | ATP bioluminescence assay | Chen et al., 2010 |
| Lipoteichoic acid treatment | Human neutrophil culture | Upregulation of *ttyh3* | RNA-Seq | Yen et al., 2019 |
| Overexpression of *ttyh1* | HEK293T Cells | VRAC currents similar to those of native astrocytes | Whole cell patch clamp recording | Han et al., 2019 |
| Overexpression of *ttyh2* | HEK293T Cells | VRAC currents similar to those of native astrocytes | Whole cell patch clamp recording | Han et al., 2019 |
| Overexpression of *ttyh3* | HEK293T Cells | VRAC currents similar to those of native astrocytes | Whole cell patch clamp recording | Han et al., 2019 |
| Overexpression of *ttyh1* | CHO-K1 Cells | VRAC currents similar to those of native astrocytes | Whole cell patch clamp recording | Han et al., 2019 |
| Overexpression of *ttyh2* | CHO-K1 Cells | VRAC currents similar to those of native astrocytes | Whole cell patch clamp recording | Han et al., 2019 |
| Overexpression of *ttyh3* | CHO-K1 Cells | VRAC currents similar to those of native astrocytes | Whole cell patch clamp recording | Han et al., 2019 |
| Vitamin D3 treatment | Caco-2 cells | Downregulation of *ttyh1* | RNA-Seq | Claro da Silva et al 2016 |

**Supplementary Table 2.** Expression of *ttyh* during development. RGD – Rat Genome Database, Steen et al., 1999; MGI – Mouse Genome Informatics, Bult et al., 2019; Developing Mouse Brain Atlas – Allen Developing Mouse Brain Atlas, 2008, https://developingmouse.brain-map.org/; Exp. Atlas – Expression Atlas, Papatheodorou et al., 2020;  Xenbase – Karimi et al., 2018; FlyBase – Thurmond et al., 2019; Fly-FISH – Lécuyer et al., 2007, Wilk et al., 2016; WormBase – Harris et al., 2019; NCBI – Brown et al., 2015;, Echinobase – Cary et al., 2018; Human Proteome Map – Kim et al., 2014; GeneCards – Stelzer et al., 2016; CNS – Central Nervous System; ISH – In situ hybridization; TPM – Transcripts Per Million; TPE – Transcripts Per Embryo; RPKM – Reads Per Kilobase Million; Low Expression Values – ≤ 10 TPM; Medium Expression Values – 11-1000 TPM; Note: range endpoints are rounded to encapsulate highest and lowest values. The paper following “Data from” provided raw data from which the proceeding database generated expression profiles/datasets. The databases list these papers as being sources for original data, so we have included them for complete transparency.

| **Stage** | **Organism** | **Expression** | **Cell/Tissue Type** | **Method** | **Source** |
| --- | --- | --- | --- | --- | --- |
| Pre-fertilization | *X. laevis* | *ttyh1* (Low)  *ttyh2*, (Low)  *ttyh3* (Low) | Oocyte | qRT-PCR | Halleran et al., 2015 |
| Pre-fertilization | *X. laevis* | *ttyh1.s* expressed (<10 TPM)  *ttyh3.s* (<10 TPM)  *ttyh3.l* (<10 TPM) | Oocyte | RNA-seq | Xenbase; Data from  Sessions et al., 2016 |
| Pre-fertilization | *M. musculus* | *ttyh1*, (0.4-0.6 TPM)  *ttyh2*, (2-3 TPM  *ttyh3* (4-5 TPM) | Oocyte | RNA-seq | Exp. Atlas; Data from Pfeiffer et al., 2014 |
| Pre-fertilization | *M. musculus* | *ttyh2* (Low expression values)  *ttyh3* (Medium expression values) | Oocyte | RNA-seq | MGI, Data from Huang et al., 2017 |
| Zygote/1 cell stage | *X. laevis* | *ttyh1*.s (7.46 TPM)  *ttyh3.s* (11.44 TPM)  *ttyh3.l* (27.13 TPM) | Zygote | RNA-seq | Xenbase; Data from Sessions et al., 2016 |
| Zygote/1 cell stage | *X. tropicalis* | *ttyh1* (35868TPE)  *ttyh2* (620 TPE)  *ttyh3 (*334183 TPE*)* | Fertilized oocyte | RNA-seq | Xenbase; Data from Owens et al., 2016 |
| Zygote/1 cell stage | *D. rerio*  (zebrafish) | *ttyh2*, (~9-10 TPM)  *ttyh2l* (92-108 TPM)  *ttyh3a (11-14 TPM)*  *ttyh3b (Below cutoff-<10 TPM)* | Fertilized oocyte | RNA-seq | White et al., 2017 |
| Zygote/1 cell stage | *M. musculus* | *ttyh1* (<0.5 TPM)  *ttyh2 (low TPM values)*  *ttyh3 (medium TPM values)* | N/A | RNA-seq | MGI; Data from Huang et al., 2017 |
| 2 cell stage | *D. rerio* | *ttyh1* (<0.5 TPM)  *ttyh2* (10-14 TPM)  *ttyh2l* (58 - 67 TPM)  *ttyh3*a (15-20 TPM)  *ttyh3*b (Below cutoff - <10 TPM) | N/A | RNA-seq | White et al., 2017 |
| 2 cell stage | *M. musculus* | *ttyh*1 (low TPM)  *ttyh*2 (moderate TPM values)  *ttyh*3 (moderate TPM values) | N/A | RNA-seq | MGI; Data from Huang et al., 2017 |
| Blastula stages | *X. laevis* | *ttyh*1 (Medium at stage 7, low stages 8-9)  *ttyh*2 (Low)  *ttyh*3 (Medium) | N/A | qRT-PCR | Halleran et al., 2015 |
| Blastula stages  (Mid-blastula; Stage 8) | *X. laevis* | *ttyh1.s* (4.04 TPM)  *ttyh2* (<1 TPM)  *ttyh3.s* (13.14 TPM)  *ttyh3.l*(51.56 TPM) | N/A | RNA-seq | Xenbase; Data from Sessions et al., 2016 |
| Blastula stage  (Stage 8) | *X. tropicalis* | *ttyh1 (28618. 8 TPE)*  *ttyh2 (2269.06 TPE)*  *ttyh3* expressed (853986 TPE) | N/A | RNA-seq | Xenbase, Data from Owens et al., 2016 |
| Blastula stage through gastrulation | *S. purpuratus* | *ttyh2 (*<10 TPM) | N/A | RNA-seq | Echinobase; Data from Tu et al., 2014 |
| Blastula stages | *D. rerio* | *ttyh2 (<10 - 19 TPM)*  *ttyh2l* (10-29 TPM)  *ttyh3a* (16-21 TPM)  *ttyh3b* (<10 TPM) | N/A | RNA-seq | White et al., 2017 |
| Gastrulation  (stage 10, 12) | *X. laevis* | *ttyh1.s* (<10 TPM)  *ttyh2* (<10 TPM)  *ttyh3.s (10.42, <10 TPM)*  *ttyh3.l* (39.22, 18.56 TPM) | N/A | RNA-seq | Xenbase; Data from Sessions et al., 2016 |
| Gastrulation  (stage 10-12) | *X. laevis* | *ttyh1* (low)  *ttyh2* (low)  *ttyh3* | N/A | qRT-PCR | Halleran et al., 2015 |
| Gastrulation  (stage 10, 12) | *X. tropicalis* | *ttyh1* (37885.9, 35422.5 TPE)  *ttyh2 (5592.26, 67862 TPE)*  *ttyh3* (716488, 559352 TPE) | N/A | RNA-seq | Xenbase; Data from Owens et al., 2016 |
| Gastrulation | *D. rerio* | *ttyh1* expressed (<10 TPM)  *ttyh2 (<10 TPM)*  (Drops to around 1 TPM during gastrulation)  *ttyh2l* (<10 TPM)  sub-stage)  *ttyh3*a (<10 - 11 TPM)  *ttyh*3b (<10 TPM) | N/A | RNA-seq | White et al., 2017 |
| Late gastrulation/late gastrula stages | *M. musculus* | *ttyh1* broadly expressed | Broad expression | Immunohistochemistry | Kumada et al., 2010 |
| Neurula stages  (stage 15, 20) | *X. laevis* | *ttyh1.s* (<0.5 TPM, <10 TPM)  *ttyh1.l (<0.5 TPM, <10 TPM)*  *ttyh2 (37.83, 34.65 TPM)*  *ttyh3.s (<10 TPM)*  *ttyh3.l (10.66, 16.51 TPM)* | N/A | RNA-Seq | Xenbase; Data from Sessions et al., 2016 |
| Neurula stages  (stage 14-19) | *X. laevis* | *ttyh*1    *ttyh*3 | Expression throughout CNS in early neurula stages, midbrain expression especially prominent at late neurula stages  Anterior nervous system with strong signal in forebrain and eyes, weak signal in somites | ISH | Halleran et al., 2015 |
| Neurula stages  (stage 14) | *X. laevis* | *ttyh1*  *ttyh*2    *ttyh*3 | 100x greater expression than pre-mid blastula)  100x greater expression that pre-gastrula fold increase in expression levels from midblastula) | qRT-PCR | Halleran et al., 2015 |
| Neurula stage  (stage 14) | *X. tropicalis* | *ttyh1 (16348 TPE)*  *ttyh2 (587825 TPE)*  *ttyh3 (1027550 TPE)* | N/A | RNA-Seq | Xenbase; Owen et al., 2016 |
| Segmentation  (1-4 somites, 10-13 somites) | *D. rerio* | *ttyh2l* | Adaxial cells | ISH | Zfin; Data from Thisse and Thisse, 2004 |
| Segmentation  (14-19 somite stage) | *D. rerio* | *ttyh*2  *ttyh2l* | CNS  Adaxial cells, neural tube, neuron, trigeminal placode | ISH | Zfin; Data from Thisse and Thisse, 2004 |
| Segmentation  (1-4 somites, 14-19 somites, 20-25 somites) | *D. rerio* | *ttyh1* (<10 TPM)  *ttyh2* (<10 TPM)  *ttyh2l* (<10 TPM)  *ttyh3a* (12-24 TPM)  *ttyh3b* (10-19 TPM) | N/A | RNA-Seq | White et al., 2017 |
| Tailbud stage  (Stage 25, 29/30, 40) | *X. laevis* | *ttyh1.s (<10, 12.68, <10 TPM))*  *ttyh1.l (<10, 10.54, <10 TPM)*  *ttyh2 (34.65, 13.69, <10 TPM)*  *ttyh3.s (<10 TPM)*  *ttyh3.l (16.51, <10 TPM)* | N/A | *RNA-Seq* | Xenbase; Sessions et al., 2016 |
| Pharyngula stage | *D. rerio* | *ttyh2*    *ttyh2l* | CNS, retinal inner nuclear layer, ganglion cell layer  Cranial ganglion cell layer, retina, (spinal cord during Prim 15 to Prim 25) | ISH | Zfin; Data from Thisse and Thisse, 2004 |
| Pharyngula  stage | *D. rerio* | *ttyh1* (<10 TPM)  *ttyh2* (<10 - 15 TPM)  *ttyh2l* (<10 TPM)  *ttyh3a* (<10 - 17 TPM)  *ttyh3b* (11 - 28 TPM) | N/A | RNA-Seq | White et al., 2017 |
| Hatching stage | *X. laevis* | *ttyh*1    *ttyh*3 | CNS, strongest signal in anterior region, expression in somites and otic vesicles as well  Anterior nervous system; somite signal from neurula stages disappears | ISH | Halleran et al., 2015 |
| Hatching stage | *X. tropicalis* | *ttyh1 (240577 - 1792440 TPE)*  *ttyh2 (889121 - 1455400 TPE)*  *ttyh3 (1412320 - 2878350 TPE)* | N/A | RNA-Seq | Xenbase; Owens et al., 2016 |
| Hatching stage | *X. laevis* | *ttyh1*  *ttyh2*  *ttyh3* | Continued expression in ventricular regions of developing CNS  First detection in cranial ganglia  Continues in anterior nervous system, increases in spinal cord, now present in pharyngeal arches | RNA *in situ* Hybridization | Halleran et al., 2015 |
| Hatching stage | *D. rerio* | *ttyh2*  *ttyh2l* | CNS, retinal inner nuclear layer, retinal ganglion cell layer  Cranial ganglia, retina | *In Situ* Hybridization | Zfin; Data from Thisse and Thisse, 2004 |
| Hatching stage | *D. rerio* | *ttyh1 (<10 - 15 TPM)*  *ttyh2 (<10 - 11 TPM)*  *ttyh2l (<10 - 13 TPM)*  *ttyh3a (11 - 14 TPM)*  *ttyh3b (35 - 40 TPM)* | N/A | RNA-Seq | White et al., 2017 |
| Swimming tadpole stage | *X. laevis* | *ttyh1*    *ttyh2*    *ttyh3* | Expression appears in ganglia VII and IX, retina, expression in confined neural tube to ventricular region  Prominently expressed in cranial ganglia V, VII, IX, X and outer layer of eye, Weak signal appears in whole neural tube, strong signal in the roofplate of the posterior neural tube  Expression mainly in lateral regions of neural tube, expression in ganglia V, VII, IX, and X | *In Situ* hybridization | Halleran et al., 2015 |
| Swimming tadpole stage | *X. laevis* | *ttyh1*  *ttyh2*  *ttyh3* | 100x greater expression than pre-mid blastula  100x greater expression that pre-gastrula | qRT-PCR | Halleran et al., 2015 |
| Organogenesis | *M. musculus* | *ttyh1* | Ventricular region of developing brain | ISH | Abramova et al., 2005 |
| Organogenesis | *M. musculus* | *ttyh1* | Ventricular region of developing brain | ISH | Kawaguchi et al., 2008 |
| Organogenesis  (stage 14.5) | *M. musculus* | *ttyh*1 | Strong signals in pancreas, ear, nervous system; weak signals in respiratory system, urinary system | ISH | MGI; Data from Visel et al., 2004; Visel et al., 2007 |
| Organogenesis  (stage 16.5) | *M. musculus* | *ttyh2* | Brain blood vessel | ISH | MGI; Data from Hupe et al., 2017 |
| Organogenesis  (stages 11.5, 13.5, 15.5) | *M. musculus* | *ttyh*1 | Brain | ISH | MGI; Data from Thompson et al., 2014 |
| Organogenesis  (stage 14.5) | *M. musculus* | *ttyh1*  *ttyh2* | Strong signals across CNS  Weak to moderate signals in brain and spinal cord | ISH | MGI; Data from Diez-Roux et al., 2011 |
| Organogenesis | *M. musculus* | *ttyh1 (<1 - 40 RPKM)*  *ttyh2 (1 - 7 RPKM)*  *ttyh3 (3 - 57* RPKM*)* | Relatively highest in whole brain  Relatively highest in whole brain  Present in brain, limbs, liver | RNA-seq | NCBI, Data from Yue et al., 2014 |
| Organogenesis | *M. musculus* | *ttyh1* | Brain | ISH | Developing Mouse Brain |
| Organogenesis into fetal period (stage 10.5 through stage 18.5) | *M. musculus* | *ttyh1 <10 - 216 TPM)*  *ttyh2* (<10 - 77 TPM)  *ttyh3 (16 -439) TPM* | Medium values in brain and testis, low values elsewhere  Low to medium values in heart and liver, moderate values in testis and ovaries  Medium values in ovary, testis, brain, kidney, liver, heart | RNA-Seq | Exp. Atlas |
| Organogenesis  (mid-late organogenesis) | *R. norvegicus* | *ttyh1 (<10 - 285* TPM*)*  *ttyh2 (<10 - 34* TPM*)*  *ttyh3*  (15 –348 TPM) | Medium in brain, low to medium in testis, low in heart, kidney, ovaries  Low values in heart, kidney, liver; low to medium values in the testis, brain, ovaries  Medium values in brain, heart, kidney, liver, ovaries, testis | RNA-Seq | Exp. Atlas |
| Organogenesis  (Carnegie stage 13 - stage 23) | *H. sapiens* | *ttyh1 (<10 - 183* TPM*)*  *ttyh2 (<10 - 32 TPM)*  *ttyh3 (93 - 248* TPM*)* | Primarily medium values in spinal cord and brain  Comparatively lower values across brain and spinal cord relative to *ttyh1* and *ttyh3*  Medium values across all sampled brain + spinal cord tissues | RNA-seq | Exp. Atlas |
| Larval Stage  (protruding Mouth, 5 days post fertilization) | *D. rerio* | *ttyh1 25 - 34* TPM*)*  *ttyh2 (<10* TPM*)*  *ttyh2l (14-21* TPM*)*  *ttyh3a (<10 - 14* TPM*)*  *ttyh3b (47 - 63* TPM*)* | N/A | RNA-Seq | White et al., 2017 |
| Fetal Period  (Stage 34, 35) | *R. norvegicus* | *ttyh1 (<10* TPM *- 285* TPM)  *ttyh2 (<10 - 29* TPM*)*  *ttyh3 (22 - 319* TPM | Values for expression highest in the brain  Highest individual value for expression in brain  Highest values in brain, lowest value in liver | RNA-Seq | Exp. Atlas |
| Fetal period  (Stage 19) | *R. norvegicus* | *ttyh1* | Hippocampal neuron cultures | immuno-cytochemistry | Stefaniuk et al., 2010 |
| Fetal period  (Stage 18.5) | *M. musculus* | *ttyh1* | Brain  (developingmouse.brain-map.org/gene/show/37048) | ISH | Developing Mouse Brain |
| Fetal period  (10 weeks through 20 weeks) | *H. sapiens* | *ttyh1 (<3* RPKM*)*  *ttyh2 (<3* RPKM*)*  *ttyh3 (<3* RPKM*)* | Samples taken from liver, heart, lungs, stomach, intestines, and adrenal gland; expression present in all tissues but values are all < 3 RPKM | RNA-seq | NCBI; Data from Szabo et al., 2015 |
| Fetal period  (9 - 20 weeks post-conception) | *H. sapiens* | *ttyh1 (<10 - 155 TPM)*  *ttyh2 (<10 - 43)*  *ttyh3 (21 -  252 TPM)* | Expression of all three in developing fetal brain and spinal cord | RNA-Seq | Exp. Atlas |
| Fetal period | *H. sapiens* | TTYH1  TTYH3 | Expressed in brain  Expressed in brain, testis, ovary, liver, heart, gut | Mass spectrometry | Human Proteome Map |
| Fetal period | *H. sapiens* | TTYH1  TTYH2  TTYH3 | Expressed in fetal brain  Expressed in fetal gut  Expressed in fetal heart, brain, gut, testis, and ovaries | Mass spectrometry | GeneCards |
| Post-natal period  (day 0 through day 28) | *M. musculus* | *ttyh1* (<10 TPM - 352 TPM)  *ttyh2* (<10 - 120 TPM)  *ttyh3* (<10 - 374 TPM) | Medium values in brain and testes, low values in all other sampled tissues  Medium values in the brain, testes, heart, kidney, and ovary, low to medium values in liver  Medium values in brain, testis, kidney, and ovary; low to medium values in the heart and liver | RNA-seq | Exp. Atlas |
| Post-natal period  (day 0 through day 10) | *M. musculus* | *ttyh1* | Parts of retina (retina outer nuclear layer, retina inner nuclear layer, muller glial cells) and CNS (external granule cell layer of cerebellum and ventricular layer of brain) | ISH | MGI; Data from Blackshaw et al., 2004 |
| Post-natal period  (day 4, 18, 28) | *M. musculus* | *ttyh1* | Brain | ISH | MGI; Data from Thompson et al., 2014 |
| Post-natal period  (day 4, 14, 28) | *M. musculus* | *ttyh1* | Brain  (developingmouse.brain-map.org/gene/show/37048) | ISH | Developing Mouse Brain |
| Post-natal period  (day 0 - Day 42) | *R. norgevicus* | *ttyh1 (< 10 - 599 TPM)*  *ttyh2 (<10 - 265 TPM)*  *ttyh3 (<10 - 263 TPM)* | Comparatively highest, overall moderate values largely confined to the CNS and testis.  Moderate values across several tissues (brain, ovaries, liver, etc).  Moderate values in all sampled tissues | RNA-Seq | Exp. Atlas |
| Post-natal period  (7 days to 1 year) | *C. sabaeus* | *ttyh1(<10 - 374 TPM)*  *ttyh2* (<10 - 40 TPM)  *ttyh3* (<10 - 173 TPM) | Relative highest values in the brain followed by pituitary gland, low or absent in other sampled tissues.  Relative highest levels of expression in the CNS  Relative highest levels of expression in fibroblasts | RNA-Seq | Exp. Atlas |
| Post-natal Period | *R. norgevicus* | *ttyh1* | Astrocytes and microglia cultured from rat pups | Western blots | Wiernasz et al., 2014 |
| Post-natal Period  (cell cultures from 1 day old rat pups) | *R. norgevicus* | *ttyh1* | Astrocytes, microglia, and oligodendrocyte cultures | ICC | Wiernasz et al., 2014 |
| Throughout development | *C. elegans* | *ttyh1* | N/A | RNA-seq | WormBase; Data from Levin et al., 2012 |
| Throughout development | *D. melanogaster* | *tty*  *CG3638* | Expressed in developing larval cns, pupal fat bodies.  Expressed in developing larval cns/digestive system, pupal fat bodies | RNA-seq | FlyBase; Data from Gravely et al., 2011 |
| Organogenesis, larva L1-L3, pupa Day 3 | *D. melanogaster* | tty | N/A | Mass spectrometry | FlyBase |
| Throughout development  (with exception of larva L3 wandering stage) | *D. melanogaster* | CG3638 | N/A | Mass spectrometry | FlyBase |
| Throughout development | *D. melanogaster* | *tty* | Absent from all developmental stages | FISH | Fly-FISH |

**Supplementary Table 3:** Adult *ttyh* expression across several species. RGD – Rat Genome Database, Steen et al., 1999; MGI – Mouse Genome Informatics, Bult et al., 2019; Allen Mouse Brain – Lein et al., 2007, https://mouse.brain-map.org/search/index; Mouse Spinal Cord – Allen Spinal Cord Atlas, 2008, https://mousespinal.brain-map.org/; Exp. Atlas – Expression Atlas, Papatheodorou et al., 2020; Xenbase – Karimi et al., 2018; FlyBase – Thurmond et al., 2019; WormBase – Harris et al., 2019; NCBI – NCBI Resource Coordinators, 2018; ProteomicsDB – Schmidt et al., 2017, Samaras et al., 2020; Human Proteome Map – Kim et al., 2014; GeneCards – Stelzer et al., 2016; GTEx Consortium – GTEx Consortium, 2015; ISH – In situ hybridization; Mass Spec. – Mass spectrometry; TPM – Transcripts Per Million; RPKM – Reads Per Kilobase Million; sFPKM – Significant Fragments Per Kilobase Million; Note: specific values are provided for RNA-Seq data, RNA-Seq data range endpoints are rounded to include lowest and highest values). The paper following “Data from” provided raw data from which the proceeding database generated expression profiles/datasets. The databases list these papers as being sources for original data, so we have included them for complete transparency.

| **Species** | **Tissue/ Organ/**  **Cell Type** | **Gene** | **Expression Notes/Values** | **Method** | **Source** |
| --- | --- | --- | --- | --- | --- |
| *R. norvegicus,*  *F344/Nctr* | Brain at 4 months and 1 year | *ttyh1*  *ttyh2*  *ttyh3* | 147-185 TPM  20-21 TPM  14-19 TPM | RNA-Seq | RGD; Data from Yu et al., 2014 |
| *R. norvegicus,* F344 strain | Visual cortex | *ttyh1*  *ttyh2*  *ttyh3* | 460 TPM  64 TPM  117 TPM | RNA-Seq | RGD; Data from Merkin et al., 2012 |
| *R. norvegicus,* BN/SsNHsd strain | Visual cortex | *ttyh1*  *ttyh2*  *ttyh3* | 523 TPM  32 TPM  87 TPM | RNA-Seq | RGD; Data from Merkin et al., 2012 |
| *R. norvegicus,* SD strain | Visual cortex | *ttyh1*  *ttyh2*  *ttyh3* | 609 TPM  45 TPM  103 TPM | RNA-Seq | RGD; Data from Merkin et al., 2012 |
| *R. norvegicus* | Brain | *ttyh1*  *ttyh2*  *ttyh3* | 447 - 547 TPM  13 - 24 TPM  64 - 132 TPM | RNA-Seq | RGD; Data from Naqvi et al., 2019 |
| *R. norvegicus* | Neurons | *ttyh1,* ttyh1 | N/A | ISH and Immunohistochemistry | Stefaniuk and Lukasiuk, 2010 |
| *R. norvegicus* | Hippocampus and hippocampal neurons | ttyh1 | N/A | Immunohistochemistry | Stefaniuk et al., 2010 |
| *R. norvegicus* | Brain stem, cerebral cortex, cerebellum | *ttyh1* | N/A | RT-PCR | Morciano et al., 2009 |
| *R. norvegicus* | Olfactory Bulb, cortex and cerebellum | *ttyh1* | N/A | RNA *in situ* | Morciano et al., 2009 |
| *R. norvegicus* | SOC, striatum, hippocampus | *ttyh1* | 33 (hippocampus) to 72 (SOC) tag counts | SAGE | Nothwang et al., 2006 |
| *R. norvegicus* | SOC, striatum | *ttyh2* | 5 (striatum) to 23 (SOC) tag counts | SAGE | Nothwang et al., 2006 |
| *R. norvegicus;* | Testis at 4 months and 1 year | *ttyh1*  *ttyh2*  *ttyh3* | 32, 23 TPM  8, 14 TPM  1, 10 TPM | RNA-Seq | RGD; Data from Yu et al., 2014 |
| *R. norvegicus;*  F344 | Testis | *ttyh1*  *ttyh2*  *ttyh3* | 28 TPM  25 TPM  <10 TPM | RNA-Seq | RGD; Data from Merkin et al., 2012 |
| *R. norvegicus;*  BN/SsNHsd | Testis | *ttyh1*  *ttyh2*  *ttyh3* | 16 TPM  <10 TPM  <10 TPM | RNA-Seq | RGD; Data from Merkin et al., 2012 |
| *R. norvegicus;*  SD | Testis | *ttyh1*  *ttyh2*  *ttyh3* | 29 TPM  22 TPM  <10 TPM | RNA-Seq | RGD; Data from Merkin et al., 2012 |
| *R. norvegicus* | Testis | *ttyh1 ttyh2*  *ttyh3* | 24- 29 TPM  <10 TPM  <10 TPM | RNA-Seq | RGD; Data from Naqvi et al., 2019 |
| *R. norvegicus* | Gastrocnemius at 4 months and 1 year | *ttyh1 ttyh2*  *ttyh3* | <10 TPM  <10 TPM  <10 TPM | RNA-Seq | RGD; Data from Yu et al., 2014 |
| *R. norvegicus,* F344 strain | Colon | *ttyh1*  *ttyh2*  *ttyh3* | <10 TPM  <10 TPM  32 TPM | RNA-Seq | RGD; Data from Merking et al., 2012 |
| *R. norvegicus;*  BN/SsNHsd | Colon | *ttyh1*  *ttyh2*  *ttyh3* | <10 TPM  13 TPM  43 TPM | RNA-Seq | RGD; Data from Merkin et al., 2012 |
| *R. norvegicus;*  SD | Colon | *ttyh1*  *ttyh2*  *ttyh3* | <10 TPM  <10 TPM  <10 TPM | RNA-Seq | RGD; Data from Merkin et al., 2012 |
| *R. norvegicus* | Colon | *ttyh1*  *ttyh2*  *ttyh3* | <10 TPM  <10 TPM  10 -  24 TPM | RNA-Seq | RGD; Data from Naqvi et al., 2019 |
| *R. norvegicus* | Heart at 4 months and 1 year | *ttyh1*  *ttyh2*  *ttyh3* | <10 TPM  <10 TPM  <10 TPM | RNA-Seq | RGD; Data from Yu et al., 2014 |
| *R. norvegicus* | Heart | *ttyh1*  *ttyh2*  *ttyh3* | <10 TPM  <10 TPM  <10 TPM | RNA-Seq | RGD; Data from Naqvi et al., 2019 |
| *R. norvegicus* | Liver at 4 months and 1 year | *ttyh1*  *ttyh2*  *ttyh3* | <10 TPM  24-29 TPM  <10 TPM | RNA-Seq | RGD; Data from Yu et al., 2014 |
| *R. norvegicus;*  F344 | Liver | *ttyh1*  *ttyh2*  *ttyh3* | Below 0.5 TPM cutoff  49 TPM  13 TPM | RNA-Seq | RGD; Data from Merkin et al., 2012 |
| *R. norvegicus;*  BN/SsNHsd | Liver | *ttyh1*  *ttyh2*  *ttyh3* | Below 0.5 TPM cutoff  36 TPM  <10 TPM | RNA-Seq | RGD; Data from Merkin et al., 2012 |
| *R. norvegicus;* SD | Liver | *ttyh1*  *ttyh2*  *ttyh3* | <10 TPM  24 TPM  <10 TPM | RNA-Seq | RGD; Data from Merkin et al., 2012 |
| *R. norvegicus* | Liver | *ttyh1*  *ttyh2*  *ttyh3* | Below 0.5 TPM cutoff  15  - 30 TPM  <10 TPM | RNA-Seq | RGD; Data from Naqvi et al., 2019 |
| *R. norvegicus* | Kidney at 4 months and 1 year | *ttyh1*  *ttyh2*  *ttyh3* | <10TPM  6-9 TPM  8-13 TPM | RNA-Seq | RGD; Data from Yu et al., 2014 |
| *R. norvegicus;*  F344 | Kidney | *ttyh1*  *ttyh2*  *ttyh3* | <10 TPM  <10 TPM  21 TPM | RNA-Seq | RGD; Data from Merkin et al., 2012 |
| *R. norvegicus;*  BN/SsNHsd | Kidney | *ttyh1*  *ttyh2*  *ttyh3* | <10 TPM  14 TPM  31 TPM | RNA-Seq | RGD; Data from Merkin et al., 2012 |
| *R. norvegicus;*  SD | Kidney | *ttyh1*  *ttyh2*  *ttyh3* | <10 TPM  22 TPM  31 TPM | RNA-Seq | RGD; Data from Merkin et al., 2012 |
| *R. norvegicus* | Adrenal gland at 4 months and 1 year | *ttyh1*  *ttyh2*  *ttyh3* | <10 TPM  17-31 TPM  <10 TPM | RNA-Seq | RGD; Data  from Yu et al., 2014 |
| *R. norvegicus* | Adrenal gland | *ttyh1*  *ttyh2*  *ttyh3* | <10 TPM  19  -  30 TPM  <10 - 22 TPM | RNA-Seq | RGD; Data from Naqvi et al., 2019 |
| *R. norvegicus* | Thyroid gland | *ttyh1*  *ttyh2*  *ttyh3* | <10 TPM  <10 TPM  8 - 28 TPM | RNA-Seq | RGD; Data from Naqvi et al., 2019 |
| *R. norvegicus* | Pituitary gland | *ttyh1*  *ttyh2*  *ttyh3* | 23  - 51 TPM  <10 TPM  20 - 42 TPM | RNA-Seq | RGD; Data from Naqvi et al., 2019 |
| *R. norvegicus* | Spleen at 4 months and 1 year | *ttyh1*  *ttyh2*  *ttyh3* | <10 TPM  <10 TPM  24-42 TPM | RNA-Seq | RGD; Data from Yu et al., 2014 |
| *R. norvegicus;*  F344 | Spleen | *ttyh1*  *ttyh2*  *ttyh3* | <10 TPM  <10 TPM  85 TPM | RNA-Seq | RGD; Data from Merkin et al., 2012 |
| *R. norvegicus;*  BN/SsNHsd | Spleen | *ttyh1*  *ttyh2*  *ttyh3* | <10 TPM  <10 TPM  107 TPM | RNA-Seq | RGD; Data from Merkin et al., 2012 |
| *R. norvegicus;*  SD | Spleen | *ttyh1*  *ttyh2*  *ttyh3* | <10 TPM  <10 TPM  42 TPM | RNA-Seq | RGD; Data from Merkin et al., 2012 |
| *R. norvegicus* | Spleen | *ttyh1*  *ttyh2*  *ttyh3* | <10 TPM  <10 TPM  22  -  55 TPM | RNA-Seq | RGD; Data from Naqvi et al., 2019 |
| *R. norvegicus* | Uterus at 4 months and 1 year | *ttyh1*  *ttyh2*  *ttyh3* | <10 TPM  <10 TPM  28, 18 TPM | RNA-Seq | RGD; Data from Yu et al., 2014 |
| *R. norvegicus* | Thymus at 4 months and 1 year | *ttyh1*  *ttyh2*  *ttyh3* | <10 TPM  <10 TPM  12-15 TPM | RNA-Seq | RGD; Data from Yu et al., 2014 |
| *R. norvegicus* | Lungs at 4 months and 1 year | *ttyh1*  *ttyh2*  *ttyh3* | <10 TPM  <10 TPM  25-29 TPM | RNA-Seq | RGD; Data from Yu et al., 2014 |
| *R. norvegicus;*  F344 | Lung | *ttyh1*  *ttyh2*  *ttyh3* | <10 TPM  <10 TPM  56 TPM | RNA-Seq | RGD; Data from Merkin et al., 2012 |
| *R. norvegicus;*  BN/SsNHsd | Lung | *ttyh1*  *ttyh2*  *ttyh3* | <10 TPM  11 TPM  61 TPM | RNA-Seq | RGD; Data from Merkin et al., 2012 |
| *R. norvegicus;*  SD | Lung | *ttyh1*  *ttyh2*  *ttyh3* | <10 TPM  14 TPM  82 TPM | RNA-Seq | RGD; Data from Merkin et al., 2012 |
| *R. norvegicus* | Lung | *ttyh1*  *ttyh2*  *ttyh3* | <10 TPM  <10 - 13 TPM  17 - 82 TPM | RNA-Seq | RGD; Data from Naqvi et al., 2019 |
| *R. norvegicus;*  F344 | Skeletal muscle of quadriceps femoris | *ttyh1*  *ttyh2*  *ttyh3* | <10 TPM  12 TPM  <10 TPM | RNA-Seq | RGD; Data from Merkin et al., 2012 |
| *R. norvegicus;*  BN/SsNHsd | Skeletal muscle of quadriceps femoris | *ttyh1*  *ttyh2*  *ttyh3* | Below 0.5 TPM cutoff  10 TPM  <10 TPM | RNA-Seq | RGD; Data from Merkin et al., 2012 |
| *R. norvegicus;*  SD | Skeletal muscle of quadriceps femoris | *ttyh1*  *ttyh2*  *ttyh3* | Below 0.5 TPM cutoff  16 TPM  <10 TPM | RNA-Seq | RGD; Data from Merkin et al., 2012 |
| *R. norvegicus* | Skeletal muscle | *ttyh1*  *ttyh2*  *ttyh3* | Below 0.5 TPM cutoff  <10 - 11 TPM  <10 TPM | RNA-Seq | RGD; Data from Naqvi et al., 2019 |
| *R. norvegicus* | Zone of skin | *ttyh1*  *ttyh2 ttyh3* | <10 TPM  <10 TPM  11 - 30 TPM | RNA-Seq | RGD; Data from Naqvi et al., 2019 |
| *R. norvegicus* | Visceral fat | *ttyh1 ttyh2*  *ttyh3* | <10 TPM  <10 - 16 TPM  18 - 28 TPM | RNA-Seq | RGD; Data from Naqvi et al., 2019 |
| *M. musculus* | Nervous system (cerebellum, cortex, and frontal lobe | *ttyh1*  *ttyh2*  *ttyh3* | 91 - 117 RPKM  4 - 22  RPKM  18 - 30 RPKM | RNA-seq | NCBI; Data from Yue et al, 2014 |
| *M. musculus* | Brain  (without cerebellum) | *ttyh1*  *ttyh2*  *ttyh3* | 279 TPM  46 TPM  204 TPM | RNA-Seq | Exp. Atlas; Data from Brawand et al., 2011 |
| *M. musculus* | Brain (cerebellum) | *ttyh1*  *ttyh2*  *ttyh3* | 225 TPM  108 TPM  167 TPM | RNA-Seq | Exp. Atlas; Data from Brawand et al., 2011 |
| M. musculus | Brain | *ttyh1*  *ttyh2*  *ttyh3* | 293 TPM  109 TPM  290 TPM | RNA-Seq | Exp. Atlas; Data from Barbosa-Morais et al., 2012 |
| *M. musculus*  (Strain C57BL/6) | Brain | *ttyh1*  *ttyh2*  *ttyh3* | 284 TPM  36 TPM  151 TPM | RNA-Seq | Exp. Atlas |
| *M. musculus*  (CD1) | Brain | *ttyh1*  *ttyh2*  *ttyh3* | 344 TPM  33 TPM  171 TPM | RNA-Seq | Exp. Atlas |
| *M. musculus*  (DBA/2j) | Brain | *ttyh1*  *ttyh2*  *ttyh3* | 272 TPM  68 TPM  118 TPM | RNA-Seq | Exp. Atlas |
| *M. musculus* | Brain (hippocampal formation) | *ttyh1*  *ttyh2*  *ttyh3* | 227 TPM  43 TPM  151 TPM | RNA-Seq | Exp. Atlas; Data from Keane et al., 2011 |
| M. musculus | Dorsal root ganglion | *ttyh1*  *ttyh2*  *ttyh3* | N/A | RT-PCR | Al-jumaily et al., 2007 |
| *M. musculus* | Brain | *ttyh1*  *ttyh3* | N/A | Mass spectrometry | Exp. Atlas; Data from Huttin et al.,2010 |
| *M. musculus* | Brain | *ttyh1*  *ttyh2*  *ttyh3* | Strongest signal in isocortex  (mouse.brain-map.org/experimn/show/70919830)    Strongest expression in midbrain  (mouse.brain-map.org/experiment/show/70946147)    Strongest expression in Isocortex  (mouse.brain-map.org/experiment/show/69863296)  (mouse.brain-map.org/gene/show/54179) | ISH | Allen Mouse Brain Institute |
| *M. musculus* | Spinal Cord | *ttyh1*  *ttyh2* | Noted expression in white matter  (mousespinal.brain-map.org/imageseries/detail/100004890.html)  Noted expression in white matter and “vascular like in gray and white matter”  (mousespinal.brain-map.org/imageseeries/detail/100016098.html) | ISH | Mouse Spinal Cord |
| *M. musculus* | Adult Myelinating Oligodendrocyte | *ttyh2* | N/A | Microarray | Life Map Discovery |
| *M. musculus* | Testis | *ttyh1*  *ttyh2*  *ttyh3* | 11.776 RPKM  6.12 RPKM  7.086 RPKM | RNA-Seq | NCBI; Data from Yue et al., 2014 |
| *M. musculus* | Testis | *ttyh1*  *ttyh2*  *ttyh3* | 20 TPM  20 TPM  18 TPM | RNA-Seq | Exp. Atlas; Data from Brawand et al., 2011 |
| *M. musculus*  (Strain C57BL/6) | Testis | *ttyh1*  *ttyh2*  *ttyh3* | 31 TPM  18 TPM  22 TPM | RNA-Seq | Exp. Atlas |
| *M. musculus*  (CD1) | Testis | *ttyh1*  *ttyh2*  *ttyh3* | 26 TPM  27 TPM  17 TPM | RNA-Seq | Exp. Atlas |
| *M. musculus*  (DBA/2j) | Testis | *ttyh1*  *ttyh2*  *ttyh3* | 24 TPM  23 TPM  20 TPM | RNA-Seq | Exp. Atlas |
| *M. musculus* | Testis | *ttyh1*  *ttyh3* | N/A | Mass spectrometry | Exp. Atlas; Data from s et al.,2010 |
| *M. musculus* | Digestive system | *ttyh1*  *ttyh2*  *ttyh3* | 2.046-3.878 RPKM  6.238-41.608 RPKM  7.074-23.103 RPKM | RNA-Seq | NCBI; Data from Yue et al., 2014 |
| *M. musculus*  (Strain C57BL/6) | Colon | *ttyh1*  *ttyh2*  *ttyh3* | <10 TPM  56 TPM  19 TPM | RNA-Seq | Exp. Atlas |
| *M. musculus*  (CD1) | Colon | *ttyh1*  *ttyh2*  *ttyh3* | <10 TPM  63 TPM  31 TPM | RNA-Seq | Exp. Atlas |
| *M. musculus*  (DBA/2j) | Colon | *ttyh1*  *ttyh2*  *ttyh3* | <10 TPM  101 TPM  43 TPM | RNA-Seq | Exp. Atlas |
| *M. musculus* | Heart | *ttyh1*  *ttyh2*  *ttyh3* | 0.445 RPKM  2.301 RPKM  4.377 RPKM | RNA-Seq | NCBI; Data from Yue et al., 2014 |
| *M. musculus* | Heart | *ttyh1*  *ttyh2*  *ttyh3* | < 10 TPM  <10 TPM  10 TPM | RNA-Seq | Exp. Atlas; Data from Brawand et al., 2011 |
| *M. musculus* | Heart | *ttyh1*  *ttyh2*  *ttyh3* | <10 TPM  <10 TPM  14 TPM | RNA-Seq | Exp. Atlas; Data from Barbosa-Morais et al., 2012 |
| *M. musculus* | Heart | *ttyh1*  *ttyh2*  *ttyh3* | <10 TPM  <10 TPM  11 TPM | RNA-Seq | Exp. Atlas; Data from Keane et al., 2011 |
| *M. musculus*  (CD1) | Heart | *ttyh1*  *ttyh2*  *ttyh3* | <10 TPM  14 TPM  <10 TPM | RNA-Seq | Exp. Atlas |
| *M. musculus*  (DBA/2j) | Heart | *ttyh1*  *ttyh2*  *ttyh3* | <10 TPM | RNA-Seq | Exp. Atlas |
| *M. musculus* | Liver | *ttyh2*  *ttyh3* | 26.392 RPKM  3.125 RPKM | RNA-Seq | NCBI; Data from Yue et al., 2014 |
| *M. musculus* | Liver | *ttyh2*  *ttyh3* | 52 TPM  <10 TPM | RNA-Seq | Exp. Atlas; Data from Brawand et al., 2011 |
| *M. musculus* | Liver | *ttyh1*  *ttyh2*  *ttyh3* | <10 TPM  86 TPM  <10 TPM | RNA-Seq | Exp. Atlas; Raw data originates from Barbosa-Morais et al., 2012 |
| *M. musculus* | Liver | *ttyh2*  *ttyh3* | 64 TPM  <10 TPM | RNA-Seq | Exp. Atlas; Data from Keane et al., 2011 |
| *M. musculus*  (Strain C57BL/6) | Liver | *ttyh1*  *ttyh2*  *ttyh3* | <10 TPM  71 TPM  <10 TPM | RNA-Seq | Exp. Atlas |
| *M. musculus*  (CD1) | Liver | *ttyh1*  *ttyh2*  *ttyh3* | <10 TPM  88 TPM  <10 TPM | RNA-Seq | Exp. Atlas |
| *M. musculus*  (DBA/2j) | Liver | *ttyh1*  *ttyh2*  *ttyh3* | <10 TPM  42 TPM  <10 TPM | RNA-Seq | Exp. Atlas |
| *M. musculus* | Kidney | *ttyh1*  *ttyh2*  *ttyh3* | 0.627 RPKM  14.305 RPKM  12.787 RPKM | RNA-Seq | NCBI; Data from Yue et al., 2014 |
| *M. musculus* | Kidney | *ttyh1*  *ttyh2*  *ttyh3* | <10 TPM  40 TPM  29 TPM | RNA-Seq | Exp. Atlas; Data from Brawand et al., 2011 |
| *M. musculus* | Kidney | *ttyh1*  *ttyh2*  *ttyh3* | <10 TPM  41 TPM  39 TPM | RNA-Seq | Exp. Atlas; Data from Barbosa-Morais et al., 2012 |
| *M. musculus*  (Strain C57BL/6) | Kidney | *ttyh1*  *ttyh2*  *ttyh3* | <10 TPM  53 TPM  17 TPM | RNA-Seq | Exp. Atlas |
| *M. musculus*  (CD1) | Kidney | *ttyh1*  *ttyh2*  *ttyh3* | <10 TPM  88 TPM  32 TPM | RNA-Seq | Exp. Atlas |
| *M. musculus*  (DBA/2j) | Kidney | *ttyh1*  *ttyh2*  *ttyh3* | Below 0.5 cutoff  64 TPM  24 TPM | RNA-Seq | Exp. Atlas |
| *M. musculus* | Adrenal gland | *ttyh1*  *ttyh2*  *ttyh3* | 1.888 RPKM  72.087 RPKM  79.764 RPKM | RNA-Seq | NCBI; Data from Yue et al., 2014 |
| *M. musculus* | Bladder | *ttyh1*  *ttyh2*  *ttyh3* | 1.368 RPKM  1.556 RPKM  6.08 RPKM | RNA-Seq | NCBI; Data  from Yue et al., 2014 |
| *M. musculus* | Spleen | *ttyh1*  *ttyh2*  *ttyh3* | 0.652 RPKM  8.115 RPKM  45.981 RPKM | RNA-Seq | NCBI, 2018; Data from Yue et al., 2014 |
| *M. musculus* | Spleen | *ttyh1*  *ttyh2*  *ttyh3* | <10 TPM  28 TPM  101 TPM | RNA-Seq | Exp. Atlas; Raw data originates from Keane et al., 2011 |
| *M. musculus* | Spleen | *ttyh3* | N/A | Mass spectrometry | Exp. Atlas; Data from Huttlin et al.,2010 |
| *M. musculus*  (Strain C57BL/6) | Spleen | *ttyh1*  *ttyh2*  *ttyh3* | <10 TPM  22 TPM  131 TPM | RNA-Seq | Exp. Atlas |
| *M. musculus*  (CD1) | Spleen | *ttyh1*  *ttyh2*  *ttyh3* | <10 TPM  32 TPM  126 TPM | RNA-Seq | Exp. Atlas |
| *M. musculus*  (DBA/2j) | Spleen | *ttyh1*  *ttyh2*  *ttyh3* | <10 TPM  18 TPM  103 TPM | RNA-Seq | Exp. Atlas |
| *M. musculus* | Ovary | *ttyh1*  *ttyh2*  *ttyh3* | 1.38 RPKM  39.099 RPKM  50.276 RPKM | RNA-Seq | NCBI; Data from Yue et al., 2014 |
| *M. musculus* | Placenta | *ttyh1*  *ttyh2*  *ttyh3* | 0.565 RPKM  3.235 RPKM  5.325 RPKM | RNA-Seq | NCBI; Data from Yue et al., 2014 |
| *M. musculus* | Mammary gland | *ttyh1*  *ttyh2*  *ttyh3* | 1.174 RPKM  32.439 RPKM  30.742 RPKM | RNA-Seq | NCBI; Data from Yue et al., 2014 |
| *M. musculus* | Subcutaneous fat pad | *ttyh1*  *ttyh2*  *ttyh3* | 0.391 RPKM  24.008 RPKM  15.665 RPKM | RNA-Seq | NCBI; Data from Yue et al., 2014 |
| *M. musculus* | Genital fat pad | *ttyh1*  *ttyh2*  *ttyh3* | 6.573 RPKM  12.63 RPKM  24.302 RPKM | RNA-Seq | NCBI; Data from Yue et al., 2014 |
| *M. musculus* | Thymus | *ttyh2*  *ttyh3* | 5.404 RPKM  45.757 RPKM | RNA-Seq | NCBI; Data from Yue et al., 2014 |
| *M. musculus* | Thymus | *ttyh1*  *ttyh2*  *ttyh3* | <10 TPM  15 TPM  86 TPM | RNA-Seq | Exp. Atlas; Data from Keane et al., 2011 |
| *M. musculus* | Lungs | *ttyh1*  *ttyh2*  *ttyh3* | 0.335 RPKM  8.661 RPKM  48.746 RPKM | RNA-Seq | NCBI; Data from Yue et al., 2014 |
| *M. musculus* | Lungs | *ttyh1*  *ttyh2*  *ttyh3* | <10 TPM  13 TPM  81 TPM | RNA-Seq | Exp. Atlas; Data from Keane et al., 2011 |
| *M. musculus*  (Strain C57BL/6) | Lungs | *ttyh1*  *ttyh2*  *ttyh3* | <10 TPM  11 TPM  46 TPM | RNA-Seq | Exp. Atlas |
| *M. musculus*  (CD1) | Lungs | *ttyh1*  *ttyh2*  *ttyh3* | <10 TPM  35 TPM  140 TPM | RNA-Seq | Exp. Atlas |
| *M. musculus*  (DBA/2j) | Lungs | *ttyh1*  *ttyh2*  *ttyh3* | <10 TPM  15 TPM  56 TPM | RNA-Seq | Exp. Atlas |
| *M. musculus* | Lungs | *ttyh3* | N/A | Mass spectrometry | Exp. Atlas; Data from Huttlin et al., 2010 |
| *M. musculus* | Skeletal muscle tissue | *ttyh1*  *ttyh2*  *ttyh3* | <10 TPM  15 TPM  <10 TPM | RNA-Seq | Exp. Atlas; Raw data from Barbosa-Morais et al., 2012 |
| *M. musculus*  (Strain C57BL/6) | Skeletal muscle tissue | *ttyh1*  *ttyh2*  *ttyh3* | <10 TPM  13 TPM  <10 TPM | RNA-Seq | Exp. Atlas |
| *M. musculus*  (CD1) | Skeletal muscle tissue | *ttyh1*  *ttyh2*  *ttyh3* | <10 TPM  29 TPM  <10 TPM | RNA-Seq | Exp. Atlas |
| *M. musculus*  (DBA/2j) | Skeletal muscle tissue | *ttyh1*  *ttyh2*  *ttyh3* | <10 TPM  26 TPM  <10 TPM | RNA-Seq | Exp. Atlas |
| *M. musculus* | Brain, prefrontal cortex | Ttyh1  (Q9D3A9)  Ttyh1  (Q9D3A9-4)  Ttyh1  (Q9D3A9-5)  Ttyh1  (A0A0U1RPU8)  Ttyh1  (D6RG66)  Ttyh1  (A0A0U1RPY1) | Highest in brain across all isoforms | Mass Spec. | ProteomicsDB |
| *M. musculus* | Brain | Ttyh2  (Q3TH73)  Ttyh2  (Q3TH73-2) | N/A | Mass Spec. | ProteomicsDB |
| *M. musculus* | Brain, prefrontal cortex, lung, spleen | Ttyh3  (Q6P5F7)  Ttyh3  (Q6P5F7-2) | Highest in brain, lowest in spleen for both isoforms | Mass Spec. | ProteomicsDB |
| *M. musculus* | Cortical neurons | Ttyh3 fragment  (A0A0G2JGJ4) | N/A | Mass Spec. | ProteomicsDB |
| *H. sapiens* | Brain | *TTYH2* | High expression | Northern Blot | Rae et al., 2001 |
| *H. sapiens* | Nervous System | *TTYH1*  *TTYH2*  *TTYH3* | ~71. 734 RPKM  ~43.84 RPKM  ~20.454 RPKM | RNA-Seq | NCBI; Data from Fagerberg et al., 2014 |
| *H. sapiens* | Nervous System | *TTYH1*  *TTYH2*  *TTYH3* | 42-126 TPM  <10 – 98 TPM  19 – 131 TPM | RNA-Seq | NCBI; Data from GTEx Consortium, 2015 |
| *H. sapiens* | Brain | *TTYH1*  *TTYH2*  *TTYH3* | 26.353 RPKM  28.206 RPKM  4.189 RPKM | RNA-Seq | NCBI; Data from Illumina bodymap2 transcriptome |
| *H. sapiens* | CNS (frontal cortex and spinal cord) | *TTYH1*  *TTYH3* | High expression in brain, medium expression  Medium expression in frontal cortex, no recorded expression in spinal cord | RNA-Seq | Human Proteome Map; Kim et al., 2014 |
| *H. sapiens* | Retina | TTYH1 | Medium levels of expression | Mass spectrometry | Human Proteome Map; Kim et al., 2014 |
| *H. sapiens* | Testis | *TTYH1*  *TTYH2*  *TTYH3* | ~20.024 RPKM  ~8.81 RPKM  ~7.738 RPKM | RNA-Seq | NCBI, 2018; Data from Fagerberg et al., 2014 |
| *H. sapiens* | Testis | *TTYH1*  *TTYH2*  *TTYH3* | 39 TPM  24 TPM  27 TPM | RNA-Seq | Exp. Atlas; Data from GTEx Consortium, 2015 |
| *H. sapiens* | Testis | *TTYH1*  *TTYH2*  *TTYH3* | 4.336 RPKM  2.965 RPKM  3.66 RPKM | RNA-Seq | NCBI; Data from Illumina bodymap2 transcriptome |
| *H. sapiens* | Testis | *TTYH2* | High expression | Northern Blot | Rae et al., 2001 |
| *H. sapiens* | Testis | TTYH1 | Low expression | Mass spectrometry | Human Proteome Map; Kim et al., 2014 |
| *H. sapiens* | Prostrate | *TTYH1*  *TTYH2*  *TTYH3* | 2 TPM  6 TPM  35 TPM | RNA-Seq | Exp. Atlas; Data from GTEx Consortium, 2015 |
| *H. sapiens* | Prostrate | *TTYH1*  *TTYH2*  *TTYH3* | <1 RPKM  2.294 RPKM  2.748 RPKM | RNA-Seq | NCBI; Data from Illumina bodymap2 transcriptome |
| *H. sapiens* | Ovary | *TTYH1*  *TTYH2*  *TTYH3* | 1 TPM  27 TPM  28 TPM | RNA-Seq | Exp. Atlas; Data from GTEx Consortium, 2015 |
| *H. sapiens* | Ovary | *TTYH1*  *TTYH2*  *TTYH3* | 1.186 RPKM  5.399 RPKM  3.399 RPKM | RNA-Seq | NCBI; Data from Illumina bodymap2 transcriptome |
| *H. sapiens* | Ovary | TTYH2  TTYH3 | N/R | Mass spectrometry | Human Proteome Map; Kim et al., 2014 |
| *H. sapiens* | Ovary | *TTYH2* | Levels reported as being very low | Northern blot | Rae et al., 2001 |
| *H. sapiens* | Fallopian tube | *TTYH1*  *TTYH2*  *TTYH3* | 4 TPM  11 TPM  29 TPM | RNA-Seq | Exp. Atlas; Data from GTEx Consortium, 2015 |
| *H. sapiens* | Uterus | *TTYH1*  *TTYH2*  *TTYH3* | 1 TPM  12 TPM  35 TPM | RNA-Seq | Exp. Atlas; Data from GTEx Consortium, 2015 |
| *H. sapiens* | Vagina | *TTYH1*  *TTYH2*  *TTYH3* | 2 TPM  6 TPM  19 TPM | RNA-Seq | Exp. Atlas; Data from GTEx Consortium, 2015 |
| *H. sapiens* | Breast | *TTYH1*  *TTYH2*  *TTYH3* | 3.332 RPKM  <1 RPKM  2.819 RPKM | RNA-Seq | NCBI; Data from Illumina bodymap2 transcriptome |
| *H. sapiens* | Spleen | *TTYH1*  *TTYH2*  *TTYH3* | ~0.513 RPKM  ~6.059 RPKM  ~15.094 RPKM | RNA-Seq | NCBI; data from Fagerberg et al., 2014 |
| *H. sapiens* | Spleen | *TTYH1*  *TTYH2*  *TTYH3* | 1 TPM   13 TPM   71 TPM | RNA-Seq | Exp. Atlas; Data from GTEx Consortium, 2015 |
| *H. sapiens* | Spleen | *TTYH2* | Levels/signal reported as being very low | RNA-Seq | Rae et al., 2001 |
| *H. sapiens* | Lymph node | *TTYH2*  *TTYH3* | ~3.453 RPKM  ~8.284 RPKM | RNA-Seq | NCBI; Data from Fagerberg et al., 2014 |
| *H. sapiens* | Lymph node | *TTYH1*  *TTYH2*  *TTYH3* | <1 RPKM  1.235 RPKM  6.289 RPKM | RNA-Seq | NCBI; Data from Illumina bodymap2 transcriptome |
| *H. sapiens* | White blood cells | *TTYH2*  *TTYH3* | 4.509 RPKM  15.641 RPKM | RNA-Seq | NCBI; Data from Illumina bodymap2 transcriptome |
| *H. sapiens* | Monocytes | TTYH3 | Medium-low expression | Mass spectrometry | Human Proteome Map; Kim et al., 2014 |
| *H. sapiens* | Blood leukocytes | *TTYH2* | Levels/signal reported as being very low | Northern Blot | Rae et al., 2001 |
| *H. sapiens* | Digestive system | *TTYH1*  *TTYH2*  *TTYH3* | <1 RPKM  ~0.935 -2.227 RPKM  ~1.892 - 10.453 RPKM | RNA-Seq | NCBI; Data from Fagerberg et al., 2014 |
| *H. sapiens* | Digestive system | *TTYH1*  *TTYH2*  *TTYH3* | 0.5-5 TPM  2-9 TPM  10- 41 TPM | RNA-Seq | Exp. Atlas; Data from GTEx Consortium, 2015 |
| *H. sapiens* | Colon | *TTYH1*  *TTYH2*  *TTYH3* | <1 RPKM  1.141 RPKM  1.237 RPKM | RNA-Seq | NCBI; Data from Illumina bodymap2 transcriptome |
| *H. sapiens* | Appendix | *TTYH1*  *TTYH2*  *TTYH3* | <1 RPKM  ~4.01 RPKM  ~14.84 RPKM | RNA-Seq | NCBI; Data from Fagerberg et al., 2014 |
| *H. sapiens* | Adrenal | *TTYH1*  *TTYH2*  *TTYH3* | ~ <1 RPKM  ~ 2.3 RPKM  ~6.955 RPKM | RNA-Seq | NCBI; Data from Fagerber et al., 2014 |
| *H. sapiens* | Adrenal | *TTYH2*  *TTYH3* | 4 TPM   33 TPM | RNA-Seq | Exp. Atlas; Data from GTEx Consortium, 2015 |
| *H. sapiens* | Adrenal | *TTYH1*  *TTYH2*  *TTYH3* | <1 RPKM  1.585 RPKM  6.015 RPKM | RNA-Seq | NCBI; Data from Illumina bodymap2 transcriptome |
| *H. sapiens* | Adrenal gland | TTYH3 | Medium levels of expression | Mass spectrometry | Human Proteome Map; Kim et al., 2014 |
| *H. sapiens* | Skeletal muscle | *TTYH1*  *TTYH2*  *TTYH3* | <1 RPKM  2.063 RPKM  <1 RPKM | RNA-Seq | NCBI; Data from Illumina bodymap2 transcriptome |
| *H. sapiens* | Skeletal muscle | *TTYH2* | Levels reported as being very low | Northern Blot | Rae et al., 2001 |
| *H. sapiens* | Bone marrow | *TTYH1*  *TTYH2*  *TTYH3* | ~ <1 RPKM  ~ 1.092 RPKM  ~ 3.359 RPKM | RNA-Seq | NCBI; Data from Fagerberg et al., 2014 |
| *H. sapiens* | Heart | *TTYH1*  *TTYH2*  *TTYH3* | ~ <1 RPKM  ~ 5.169 RPKM  ~ 1.654 RPKM | RNA-Seq | NCBI; Data from Fagerber et al., 2014 |
| *H. sapiens* | Heart | *TTYH1*  *TTYH2*  *TTYH3* | <1 RPKM  4.143 RPKM  1.646 RPKM | RNA-Seq | NCBI; Data from Illumina bodymap2 transcriptome |
| *H. sapiens* | Heart | *TTYH2* | Expression levels lower than those seen in brain or testis | Northern Blot | Rae et al., 2001 |
| *H. sapiens* | Sampled regions of the heart | *TTYH1*  *TTYH2*  *TTYH3* | 1 TPM, 0.6 TPM  10 TPM, 8 TPM   9 TPM, 5 TPM | RNA-Seq | Exp. Atlas; Data from GTEx Consortium, 2015 |
| *H. sapiens* | Aorta | *TTYH2*  *TTYH3* | 7 TPM  41 TPM | RNA-Seq | Exp. Atlas; Data from GTEx Consortium, 2015 |
| *H. sapiens* | Coronary artery | *TTYH1*  *TTYH2*  *TTYH3* | 1 TPM  7 TPM  35 TPM | RNA-Seq | Exp. Atlas; Data from GTEx Consortium, 2015 |
| *H. sapiens* | Tibial artery | *TTYH2*  *TTYH3* | 6 TPM  18 TPM | RNA-Seq | Exp. Atlas; Data from GTEx Consortium, 2015 |
| *H. sapiens* | Blood | *TTYH2*  *TTYH3* | 3 TPM  14 TPM | RNA-Seq | Exp. Atlas; Data from GTEx Consortium, 2015 |
| *H. sapiens* | Platelets | TTYH3 | High expression | Mass spectrometry | Human Proteome Map; Kim et al., 2014 |
| *H. sapiens* | Kidney | *TTYH2*  *TTYH3* | ~ <1 RPKM  ~15.984 RPKM | RNA-Seq | NCBI; Data from Fagerberg et al., 2014 |
| *H. sapiens* | Kidney | *TTYH1*  *TTYH2*  *TTYH3* | <1 RPKM  <1 RPKM  5.642 RPKM | RNA-Seq | NCBI; Data from Illumina bodymap2 transcriptome |
| *H. sapiens* | Liver | *TTYH2 TTYH3* | <1 RPKM  ~3.086 RPKM | RNA-Seq | NCBI; Fagerberg et al., 2014 |
| *H. sapiens* | Liver | *TTYH2*  *TTYH3* | <10 TPM  19 TPM | RNA-Seq | Exp. Atlas; Data from GTEx Consortium, 2015 |
| *H. sapiens* | Liver | *TTYH1*  *TTYH2*  *TTYH3* | <1 RPKM  <1 RPKM  4.259 RPKM | RNA-Seq | NCBI; Data from Illumina bodymap2 transcriptome |
| *H. sapiens* | Lung | *TTYH2*  *TTYH3* | ~ 2.891 RPKM  ~ 5.779 RPKM | RNA-Seq | NCBI; Data from Fagerberg et al., 2014 |
| *H. sapiens* | Lung | *TTYH2*  *TTYH3* | 8 TPM  44 TPM | RNA-Seq | Exp. Atlas; Data from GTEx Consortium, 2015 |
| *H. sapiens* | Lung | *TTYH1*  *TTYH2*  *TTYH3* | <1 RPKM  1.5 RPKM  3.57 RPKM | RNA-Seq | NCBI; Data from Illumina bodymap2 transcriptome |
| *H. sapiens* | Pancreas | *TTYH1*  *TTYH2*  *TTYH3* | ~2.706 RPKM  ~ <1 RPKM  ~ <1 RPKM | RNA-Seq | NCBI; Data from Fagerberg et al., 2014 |
| *H. sapiens* | Pancreas | *TTYH1*  *TTYH2*  *TTYH3* | 7 TPM  5 TPM  6 TPM | RNA-Seq | Exp. Atlas; Data from GTEx Consortium, 2015 |
| *H. sapiens* | Pancreas | TTYH3 | N/R | Mass spectrometry | Human Proteome Map; Kim et al., 2014 |
| *H. sapiens* | Placenta | *TTYH2*  *TTYH3* | ~ 5.466 RPKM  ~ 14.685 RPKM | RNA-Seq | NCBI; Data from Fagerberg et al., 2014 |
| *H. sapiens* | Skin | *TTYH1*  *TTYH2*  *TTYH3* | ~ <1 RPKM  ~ 2.049 RPKM  ~ 4.275 RPKM | RNA-Seq | NCBI; Data from Fagerberg et al., 2014 |
| *H. sapiens* | Testis | *TTYH1*  *TTYH2*  *TTYH3* | 4 TPM  6 TPM  19 TPM | RNA-Seq | Exp. Atlas; Data from GTEx Consortium, 2015 |
| *H. sapiens* | Transformed skin fibroblast | *TTYH2*  *TTYH3* | 4 TPM  72 TPM | RNA-Seq | Exp. Atlas; Data from GTEx Consortium, 2015 |
| *H. sapiens* | Thyroid | *TTYH2*  *TTYH3* | ~ 6.089 RPKM  ~ 2.956 RPKM | RNA-Seq | NCBI; Data from Fagerberg et al., 2014 |
| *H. sapiens* | Thyroid gland | *TTYH2*  *TTYH3* | 15 TPM  41 TPM | RNA-Seq | Exp. Atlas; Data from GTEx Consortium, 2015 |
| *H. sapiens* | Thyroid gland | *TTYH2*  *TTYH3* | 1.864 RPKM  3.304 RPKM | RNA-Seq | NCBI; Data from Illumina bodymap2 transcriptome |
| *H. sapiens* | Fat | *TTYH1*  *TTYH2*  *TTYH3* | ~ <1 RPKM  ~ 2.15 RPKM  ~ 4.111 RPKM | RNA-Seq | NCBI; Data from Fagerberg et al., 2014 |
| *H. sapiens* | Adipose | *TTYH2*  *TTYH3* | <1 RPKM  3.662 RPKM | RNA-Seq | NCBI; Data from Illumina bodymap2 transcriptome |
| *H. sapiens* | Various tissues/organs from CNS, reproductive system, digestive system, circulatory system, integumentary system, urinary system, endocrine system, immune system, muscular system, | (TTYH1)  376530.7  376531.3  489425.5  467939.1  492920.5  301194.8  425969.5  487134.5  423529.5  391739.7  445095.5  462769.1  462757.5  476863.1  461302.5  478036.1  476757.5 | 0 (several tissues) – 170 (nucleus accumbens) read counts  0 (several tissues) - 48.9 (brain, cortex) read counts  0 (several tissues) – 16.2 (nucleus accumbens)  0 – 20.3 (substantia nigra)  0 – 41.3 (spinal cord, c-1)  0 – 22.4 (caudate nucleus)  Values are well below 10 read counts regardless of sampled tissue, often resting around 0 even in the CNS | RNA-Seq | GTEx Consortium, 2015 |
| *H. sapiens* | Various tissues/organs from CNS, reproductive system, digestive system, circulatory system, integumentary system, urinary system, endocrine system, immune system, muscular system, | (*TTYH2*)  441391.6  269346.8  578825.5  534346.5  528128.2  529107.5  526858.1  534039.1  528152.5 | 0.185 (liver) – 17.1 (spinal cord) read counts  0.215 (liver) – 60.5 (spinal cord) read counts    Values all other splicing variants are incredibly low; typically, being outright absent in non-CNS tissues are rarely above 1 read count even within the CNS | RNA-Seq | GTEx Consortium, 2015 |
| *H. sapiens* | Various tissues/organs from CNS, reproductive system, digestive system, circulatory system, integumentary system, urinary system, endocrine system, immune system, muscular system, | 407643.5  403167.5  258796.11  429448.1  477439.1  498454.1  400376.2 | 0.36 (skeletal muscle) – 16.5 (cerebellum) read counts  0.58 (skeletal muscle) – 32.2 (cerebellum)  0.500 (skeletal muscle) – 39.4 (cerebellum); high expression in fibroblasts relative to other splicing variants  0 (several tissues) – 12.9 (cerebellum)  Read counts well below ten regardless of tissue | RNA-Seq | GTEx Consortium, 2015 |
| *H. sapiens* | Various Tissues/Organs from Nervous System | TTYH1  TTYH2  TTYH3 | 42-126 TPM depending on specific region sampled  7-98 TPM depending on specific region sampled  19-131 TPM depending on specific region | RNA-Seq | Exp. Atlas; Data from GTEx Consortium, 2015 |
| *H. sapiens* | Prefrontal cortex, brain, spinal cord, retina, testis, adrenal gland, | TTYH1  (Q9H313)  TTYH1  (Q9H313-2)  TTYH1  (Q9H313-3)  TTYH1  (Q9H313-4)  TTYH1  (Q9H313-4)  TTYH1  (Q9H313-5) | Highest in prefrontal cortex; lowest in adrenal gland for all isoforms | Mass-Spectrometry | ProteomicsDB; |
| *H. sapiens* | Brain, retina, prefrontal cortex | TTYH1 Fragment (E7ET67)  TTYH1  (F8WBE6)  TTYH1  (G8JLI0) | Highest expression in brain; lowest in prefrontal cortex  Expression only in brain  Highest expression in brain; lowest prefrontal cortex | Mass-spectrometry | ProteomicsDB; |
| *H. sapiens* | Breast, colon muscle, brain, colon, gut, prefrontal cortex, testis, adrenal gland, salivary gland, blood platelet, spleen | TTYH2  (Q9BSA4)  TTYH2  (E7ET67) | Highest in breast; lowest in spleen for both | Mass-spectrometry | ProteomicsDB |
| *H. sapiens* | Brain, prefrontal cortex, adrenal gland, spleen | TTYH2  (Q9BSA4-2) | Highest in brain; lowest in spleen | Mass-spectrometry | ProteomicsDB: |
| *H. sapiens* | Bone, prostate gland, prefrontal cortex, gut, adrenal gland, brain, breast, rectum, colon, spleen, gall bladder, kidney, pancreas, placenta, ovary, blood platelet, lung, monocyte, testis, heart, tonsil, lymph node, thyroid gland, salivary gland, esophagus, urinary bladder | TTYH3  TTYH3 (isoform 2)  TTYH3  (Isoform 3)  TTYH3  (Isoform 4) | Highest expression in bone for all isoforms | Mass-spectrometry | ProteomicsDB |
| *H. sapiens* | Blood platelet,  adrenal gland, brain, prefrontal cortex, placenta, pancreas, ovary, lung, testis, rectum, gall bladder, thyroid gland, tonsil, kidney, liver, spleen | TTYH3  (protein fragment) | Highest expression in blood platelet | Mass spectrometry | ProteomicsDB |
| *H. sapiens* | Adrenal, bone marrow, brain, colon, duodenum, esophagus, fallopian tube, gallbladder, kidney, lung, lymph node, pancreas, pituitary gland, placenta, prostrate, rectum, salivary gland, small intestine, spleen, stomach, testis, tonsil | TTYH2  TTYH3 | N/A | Mass spectrometry | Exp. Atlas; Data from Wang et al., 2019 |
| *H. sapiens* | Heart, smooth muscle | TTYH2 | N/A | Mass spectrometry | Exp. Atlas; Wang et al., 2019 |
| *H. sapiens* | Adipose tissue, liver, ovary, urinary bladder, vermiform appendix | *TTYH3* | N/A | Mass spectrometry | Exp. Atlas; Wang et al., 2019 |
| *H. sapiens* | Frontal cortex, spinal cord, retina, cervix, testis | TTYH1 | Highest expression in frontal cortex; lowest in testis | Mass spectrometry | GeneCards; |
| *H. sapiens* | Frontal cortex, colon muscle, breast, platelet | TTYH2 | Highest in the breast | Mass spectrometry | GeneCards; |
| *H. sapiens* | Frontal cortex, monocytes, peripheral blood mononuclear cell, platelet, bone, adrenal, breast, pancreas, placenta | TTYH3 | Highest in the peripheral blood mononuclear cells | Mass-spec. | GeneCards; |
| *P. troglodytes*  (chimpanzee) | Brain, cerebellum, ovary, testes, heart, colon, lymph node, whole blood, pituitary gland | *ttyh1*  *ttyh2*  *ttyh3* | Values range from 0.63 sFPKM (skeletal muscle) to 65.5 (brain)  Values range from 0.36 sFPKM (pituitary Gland) to 32.8 (cerebellum)  Values range from 2.05 (pituitary) to 65.5 (brain) | RNA-Seq | NCBI AceView; Data from Pipes et al., 2013 |
| *P. troglodytes* | Kidney, liver, skeletal muscle, lung, whole blood | *ttyh2*  *ttyh3* | Values range from 0.36 sFPKM (liver) to 7.64 sFPKM (whole blood)  Values range from 0.63 sFPKM (skeletal muscle)  to 18.8 (kidney) | RNA-seq | NCBI AceView; Data from Pipes et al., 2013 |
| *M. nemestrina* | Thymus, lymph node, spleen, bone marrow, colon, lung, skeletal muscle, heart, liver, kidney, pituitary gland, brain, cerebellum | *ttyh1*  *ttyh2*  *ttyh3* | Values range from 0.06 sFPKM (liver) to 35.1 (brain)  Values range from 0.16 sFPKM (liver) 17.6 (brain)  Values range from 0.42 sFPKM (skeletal muscle) to 12.4 sFPKM (bone marrow) | RNA-Seq | NCBI AceView; Data from Pipes et al., 2013 |
| *M. nemestrina* | Whole blood | *ttyh2*  *ttyh3* | 0.83 sFPKM  0.63 sFPKM | RNA-Seq | NCBI AceView; Data from Pipes et al., 2013 |
| *M. fuscata* | Cerebellum, brain, pituitary, liver, heart, skeletal muscle, lung, thymus | *ttyh1*    *ttyh2*  *ttyh3* | Values range from 0.05 sFPKM (skeletal muscle) to 46.3 sFPKM (cerebellum)  Values range from 0.18 sFPKM (liver) to 30.6 sFPKM (cerebellum)  Values range from 0.51 sFPKM (liver) 8.78 (brain) | RNA-Seq | NCBI AceView; Data from Pipes et al., 2013 |
| *M. fuscata* | Kidney, bone marrow, lymph nodes, whole blood | *ttyh2*  *ttyh3* | Values range from 0.19 sFPKM (whole blood) to 2.19 (bone marrow)  Values range from 0.42 sFPKM (whole blood) to 2.70 sFPKM (kidney) | RNA-Seq | NCBI AceView; Data from Pipes et al., 2013 |
| *M. mulatta* | Cerebellum, brain, pituitary, ovary, testis, kidney, liver, heart, skeletal muscle, lung, thymus | *ttyh1*    *ttyh2*  *ttyh3* | Values range from 0.10 sFPKM (kidney, liver, skeletal muscle) to 28.5 sFPKM (brain)  Values range from 0.12 sFPKM (liver) to 9.41 sFPKM (heart)  Values range 0.24 sFPKM (skeletal muscle) to 4.39 sFPKM (cerebellum) | RNA-Seq | NCBI AceView; Data from Pipes et al., 2013 |
| *M. mulatta* | Brain | *ttyh1*  *ttyh2*  *ttyh3* | 219 TPM  104 TPM  84 TPM | RNA-Seq | Exp. Atlas;  Data from Merkin et al., 2012 |
| *M. mulatta* | Brain | *ttyh1*  *ttyh2*  *ttyh3* | 123 TPM  94 TPM  111 TPM | RNA-Seq | Exp. Atlas;  Data from Brawand et al., 2011 |
| *M. mulatta* | Cerebellum | *ttyh1*  *ttyh2*  *ttyh3* | 109 TPM  68 TPM  217 TPM | RNA-Seq | Exp. Atlas;  Data from Brawand et al., 2011 |
| *M. mulatta* | Testis | *ttyh1*  *ttyh2*  *ttyh3* | 135 TPM  61 TPM  69 TPM | RNA-Seq | Exp. Atlas;  Data from Merkin et al., 2012 |
| *M. mulatta* | Testis | *ttyh1*  *ttyh2*  *ttyh3* | 32 TPM  17 TPM  20 TPM | RNA-Seq | Exp. Atlas;  Data from Brawand et al., 2011 |
| *M. mulatta* | Colon | *ttyh1*  *ttyh2*  *ttyh3* | <10 TPM  <10 TPM  29 TPM | RNA-Seq | Exp. Atlas;  Data from Merkin et al., 2012 |
| *M. mulatta* | Kidney | *ttyh2*  *ttyh3* | <10 TPM  49 TPM | RNA-Seq | Exp. Atlas;  Data from Merkin et al., 2012 |
| *M. mulatta* | Kidney | *ttyh1*  *ttyh2*  *ttyh3* | Below 0.5 cutoff  <10 TPM  34 TPM | RNA-Seq | Exp. Atlas;  Data from Brawand et al., 2011 |
| *M. mulatta* | Heart | *ttyh1*  *ttyh2*  *ttyh3* | Below 0.5 cutoff  <10 TPM  62 TPM | RNA-Seq | Exp. Atlas;  Data from Merkin et al., 2012 |
| *M. mulatta* | Heart | *ttyh1*  *ttyh2*  *ttyh3* | <10 TPM  19 TPM  12 TPM | RNA-Seq | Exp. Atlas;  Data from Brawand et al., 2011 |
| *M. mulatta* | Lung | *ttyh1*  *ttyh2*  *ttyh3* | Below 0.5 cutoff  <10 TPM  28 TPM | RNA-Seq | Exp. Atlas;  Data from Merkin et al., 2012 |
| *M. mulatta* | Skeletal Muscle | *ttyh1*  *ttyh2*  *ttyh3* | Below 0.5 cutoff  32 TPM  <10 TPM | RNA-Seq | Exp. Atlas;  Data from Merkin et al., 2012 |
| *M. mulatta*  (Rhesus macaque, Indian) | Whole blood, spleen, bone marrow | *ttyh2*  *ttyh3* | Values range from 0.34 sFPKM (whole blood) to 2.90 sFPKM (spleen)  Values range from 0.59 sFPKM (whole blood) to 3.82 sFPKM (bone marrow) | RNA-Seq | NCBI AceView; Data from Pipes et al., 2013 |
| *M. mulatta* | Spleen | *ttyh1*  *ttyh2*  *ttyh3* | Below 0.5 cutoff  20 TPM  60 TPM | RNA-Seq | Exp. Atlas;  Data from Merkin et al., 2012 |
| *M. mulatta*  (Rhesus macaque, Chinese) | Liver | *ttyh1*  *ttyh2*  *ttyh3* | 0.18 sFPKM  1.18 sFPKM  8.78 sFPKM | RNA-Seq | NCBI AceView; Pipes et al., 2013 |
| *M. mulatta* | Liver | *ttyh1*  *ttyh2*  *ttyh3* | Below 0.5 cutoff  <10 TPM  13 TPM | RNA-Seq | Exp. Atlas;  Data from Merkin et al., 2012 |
| *M. mulatta* | Liver | *ttyh2*  *ttyh3* | <10 TPM  27 TPM | RNA-Seq | Exp. Atlas;  Data from Brawand et al., 2011 |
| *M. mulatta*  (Rhesus macaque, chines) | Whole blood | *ttyh2*  *ttyh3* | 0.51 sFPKM  1.66 sFPKM | RNA-Seq | NCBI AceView; Pipes et al., 2013 |
| *M. fascicularis*  (Cynomolgus macaque Mauritian) | Cerebellum, brain, liver, heart, pituitary gland, lung, colon, lymph node, spleen, thymus | *ttyh1*  *ttyh2*  *ttyh3* | Values range from 0.10 sFPKM (lung) to 49.7 sFPKM (brain)  Values range from 0.48 sFPKM (liver) to 20.2 sFPKM (brain)  Values range from1.55 sFPKM (heart) to 30.6 sFPKM (cerebellum) | RNA-Seq | NCBI AceView; Pipes et al., 2013 |
| *M. fascicularis*  (Cynomolgus macaque Mauritian) | Kidney, skeletal muscle, whole blood | *ttyh2*    *ttyh3* | Values range from 0.36 sFPKM (kidney) to 2.70 sFPKM (skeletal muscle)  Values range from 0.27 sFPKM (skeletal muscle) to 3.82 sFPKM (liver) | RNA-Seq | NCBI AceView; Pipes et al., 2013 |
| *M. fascicularis* | Cerebellum, brain, pituitary, kidney, liver, heart, lung, colon, bone marrow, lymph node, thymus | *ttyh1*    *ttyh2*  *ttyh3* | Values range from 0.08 sFPKM (bone marrow, lymph node) to 46.3 sFPKM (brain)  Values range from 0.55 sFPKM (lung) to 49.7 sFPKM (brain)  Values range from 0.96 sFPKM (heart) to 8.78 (liver) | RNA-Seq | NCBI AceView; Pipes et al., 2013 |
| *M. fascicularis* | Spleen, whole blood, skeletal muscle | *ttyh2*    *ttyh3* | Values range from 0.78 sFPKM (whole blood) to 4.10 sFPKM (skeletal muscle)  Values range from 0.26 (skeletal muscle) to 4.10 (spleen) | RNA-Seq | NCBI AceView; Pipes et al., 2013 |
| *P. anubis* | Cerebellum, brain, kidney, liver, pituitary, heart, liver, colon, lung, lymph node, thymus, bone marrow | *ttyh1*  *ttyh2*  *ttyh3* | Values range from 0.08 sFPKM (spleen) to 57.1 sFPKM (brain)  Values range from 0.16 sFPKM (liver) to 12.4 sFPKM (brain)  Values range from 1.66 sFPKM (heart) to 75.3 sFPKM (bone marrow) | RNA-Seq | NCBI AceView; Pipes et al., 2013 |
| *P. anubis* | Skeletal muscle, whole blood | *ttyh2*  *ttyh3* | Values range from 0.26 sFPKM (whole blood), 6.65 (skeletal muscle)  Values range from 0.42 sFPKM (skeletal muscle) to 0.96 sFPKM (whole blood) | RNA-Seq | NCBI AceView; Pipes et al., 2013 |
| *C. atys* | Brain, cerebellum, pituitary gland, kidney, liver, heart, skeletal muscle, lung, colon, spleen, lymph node, thymus | *ttyh1*    *ttyh2*  *ttyh3* | Values range from 0.07 sFPKM (heart) to 37.6 sFPKM (brain, cerebellum)  Values range from 0.96 sFPKM (lung) to 12.4 sFPKM (thymus)  Values range from 2.70 sFPKM (liver) to 10.1 sFPKM (spleen) | RNA-Seq | NCBI AceView; Pipes et al., 2013 |
| *C. atys* | Bone marrow, whole blood | *ttyh2*  *ttyh3* | Values range from 0.26 sFPKM (whole blood, 1.78 sFPKM (bone marrow)  Values range from 3.10 sFPKM (bone marrow), 0.96 sFPKM (whole blood) | RNA-Seq | NCBI AceView; Pipes et al., 2013 |
| *C. jacchus* | Brain, pituitary gland, heart, skeletal muscle, colon, lung, bone marrow, spleen | *ttyh1*  *ttyh2*  *ttyh3* | Values range from 0.08 sFPKM (skeletal muscle) to 40.3 sFPKM (brain)  Values range from 0.17 sFPKM (colon) to 5.79 sFPKM (brain)  Values range from 1.02 sFPKM (colon) to 5.40 sFPKM (brain) | RNA-Seq | NCBI AceView; Pipes et al., 2013 |
| *C. jacchus* | Lymph node, liver, kidney | *ttyh2*  *ttyh3* | Values range from 0.22 sFPKM (liver) to 1.45 sFPKM (lymph node)  Values range from 0.26 sFPKM (liver) to 2.19 (kidney) | RNA-Seq | NCBI AceView; Pipes et al., 2013 |
| *S. sciureus* | Cerebellum, brain, kidney, heart, skeletal muscle, colon, lymph node, bone marrow | *ttyh1*  *ttyh2*  *ttyh3* | Values range from 0.10 sFPKM (bone marrow) to 37.6 sFPKM (Brain)  Values range from 0.32 sFPKM (colon) to 5.04 sFPKM (brain)  Values range from 0.17 sFPKM (skeletal muscle) to 17.6 sFPKM (cerebellum) | RNA-Seq | NCBI AceView; Pipes et al., 2013 |
| *S. sciureus* | Spleen, liver, lung | *ttyh2*  *ttyh3* | Values range from 0.11 sFPKM (liver) to 2.35 sFPKM (spleen)  Values range from 0.55 sFPKM (liver) to 4.39 sFPKM (spleen) | RNA-Seq | NCBI AceView; Pipes et al., 2013 |
| *S. sciureus* | Whole blood | *ttyh3* | 0.72 sFPKM | RNA-Seq | NCBI AceView; Pipes et al., 2013 |
| *Microbeus*  (Mouse lemur, species unspecified) | Brain, cerebellum, kidney, lung, colon, spleen | *ttyh1*  *ttyh2*  *ttyh3* | Values range from 0.11 sFPKM (lung) to 49.7 sFPKM (brain)  Values range from 0.11 sFPKM (lung) to 1.45 sFPKM (spleen)  Values range from 2.35 sFPKM (colon) to 18.8 sFPKM (kidney) | RNA-Seq | NCBI AceView; Pipes et al., 2013 |
| *Microbeus*  (Mouse lemur, species unspecified) | Liver | *ttyh1*  *ttyh3* | 0.12 sFPKM  1.18 sFPKM | RNA-Seq | NCBI AceView; Pipes et al., 2013 |
| *Microbeus*  (Mouse lemur, species unspecified) | Skeletal muscle | *ttyh2*  *ttyh3* | 0.42 sFPKM  0.26 sFPKM | RNA-Seq | NCBI AceView; Pipes et al., 2013 |
| *Aotus*  (Owl Monkey, species unspecified) | Whole blood | *ttyh3* | 1.10 sFPKM | RNA-Seq | NCBI AceView; Pipes et al., 2013 |
| *L. catta* | Whole blood | *ttyh3* | 0.96 sFPKM | RNA-Seq | NCBI AceView; Pipes et al., 2013 |
| *C. lupus* *familiaris* | Brain | *ttyh1*  *ttyh2*  *ttyh3* | 250 - 354 TPM  10 - 48 TPM  101 - 231 TPM | RNA-seq | RGD;  Data from Naqvi et al., 2019 |
| *C. lupus* *familiaris* | Testis | *ttyh1*  *ttyh2*  *ttyh3* | 62 - 76 TPM  <10 TPM  <10 TPM | RNA-seq | RGD;  Data from Naqvi et al., 2019 |
| *C. lupus* *familiaris* | Spleen | *ttyh1*  *ttyh2*  *ttyh3* | <10 - 17 TPM  <10 - 11 TPM  75 - 264 TPM | RNA-seq | RGD;  Data from Naqvi et al., 2019 |
| *C. lupus* *familiaris* | Transverse colon | *ttyh1*  *ttyh2*  *ttyh3* | <10 TPM  <10 TPM  14 - 28 TPM | RNA-seq | RGD;  Data from Naqvi et al., 2019 |
| *C. lupus* *familiaris* | Heart left ventricle | *ttyh2*  *ttyh3* | <10 TPM  15 - 34 TPM | RNA-seq | RGD;  Data from Naqvi et al., 2019 |
| *C. lupus* *familiaris* | Liver | *ttyh2*  *ttyh3* | <10 TPM  31 - 57 TPM | RNA-seq | RGD;  Raw data from Naqvi et al., 2019 |
| *C. lupus* *familiaris* | Lung | *ttyh1*  *ttyh2*  *ttyh3* | <10 TPM  <10 TPM  59 - 103 TPM | RNA-seq | RGD;  Raw data from Naqvi et al., 2019 |
| *C. lupus* *familiaris* | Adrenal gland | *ttyh1*  *ttyh2*  *ttyh3* | Below 0.5 cutoff - <10 TPM  Below 0.5 cutoff - <10 TPM  19 -38 TPM | RNA-seq | RGD;  Raw data from Naqvi et al., 2019 |
| *C. lupus* *familiaris* | Pituitary gland | *ttyh1*  *ttyh2*  *ttyh3* | Below 0.5 cutoff - 14 TPM  <10 TPM  27 - 47 TPM | RNA-seq | RGD;  Raw data from Naqvi et al., 2019 |
| *C. lupus*  *familiaris* | Thyroid gland | *ttyh1*  *ttyh2*  *ttyh3* | Below 0.5 cutoff - <10 TPM  < 10 TPM  25 - 30 TPM | RNA-seq | RGD;  Raw data from Naqvi et al., 2019 |
| *C. lupus* *familiaris* | Zone of skin | *ttyh1*  *ttyh2*    *ttyh3* | Below 0.5 cutoff - <10 TPM  <10 TPM  33 - 54 TPM | RNA-seq | RGD;  Data from Naqvi et al., 2019 |
| *C. lupus* *familiaris* | Skeletal muscle tissue | *ttyh2*  *ttyh3* | <10 TPM  <10 - 13 TPM | RNA-seq | RGD;  Data from Naqvi et al., 2019 |
| *C. lupus* *familiaris* | Visceral fat | *ttyh1*  *ttyh2*  *ttyh3* | Below 0.5 cutoff - <10 TPM  <10 TPM  31 - 98 TPM | RNA-seq | RGD;  Data from Naqvi et al., 2019 |
| *B. taurus* | Brain | *ttyh1*  *ttyh2*  *ttyh3* | 215 TPM  120 TPM  52 TPM | RNA-Seq | Exp. Atlas; Data from Merkin et al., 2012 |
| *B. taurus* | Hypothalamus | *ttyh1*  *ttyh2*  *ttyh3* | 272 TPM  328 TPM  25 TPM | RNA-Seq | Exp. Atlas; Data from Liao et al., 2014 |
| *B. taurus* | Testis | *ttyh1*  *ttyh2*  *ttyh3* | 155 TPM  15 TPM  35 TPM | RNA-Seq | Exp. Atlas; Data from Merkin et al., 2012 |
| *B. taurus* | Spleen | *ttyh1*  *ttyh2*  *ttyh3* | <10 TPM  <10 TPM  11 TPM | RNA-Seq | Exp. Atlas; Data from Merkin et al., 2012 |
| *B. taurus* | Colon | *ttyh1*  *ttyh2*  *ttyh3* | <10 TPM  26 TPM  <10 TPM | RNA-Seq | Exp. Atlas; Data from Merkin et al., 2012 |
| *B. taurus* | Duodenum | *ttyh1*  *ttyh2*  *ttyh3* | <10 TPM | RNA-Seq | Exp. Atlas; Data from Liao et al., 2014 |
| *B. taurus* | Heart | *ttyh1*  *ttyh2*  *ttyh3* | <10 TPM  <10 TPM  16 TPM | RNA-Seq | Exp. Atlas; Data from Merkin et al., 2012 |
| *B. taurus* | Liver | *ttyh1*  *ttyh2*  *ttyh3* | <10 TPM  67 TPM  31 TPM | RNA-Seq | Exp. Atlas; Data from Merkin et al., 2012 |
| *B. taurus* | Liver | *ttyh1*  *ttyh2*  *ttyh3* | <10 TPM  55 TPM  28 TPM | RNA-Seq | Exp. Atlas; Data from Liao et al., 2014 |
| *B. taurus* | Kidney | *ttyh1*  *ttyh2*  *ttyh3* | <10 TPM  21 TPM  52 TPM | RNA-Seq | Exp. Atlas; Data from Merkin et al., 2012 |
| *B. taurus* | Kidney | *ttyh1*  *ttyh2*  *ttyh3* | <10 TPM  18 TPM  40 TPM | RNA-Seq | Exp. Atlas; Data from Liao et al., 2014 |
| *B. taurus* | Lung | *ttyh1*  *ttyh2*  *ttyh3* | <10 TPM  19 TPM  82 TPM | RNA-Seq | Exp. Atlas; Data from Merkin et al., 2012 |
| *B. taurus* | Lung | *ttyh1*  *ttyh2*  *ttyh3* | <10 TPM  13 TPM  107 TPM | RNA-Seq | Exp. Atlas; Data from Liao et al., 2014 |
| *B. taurus* | Skeletal muscle Tissue | *ttyh1*  *ttyh2*  *ttyh3* | <10 TPM  <10 TPM  19 TPM | RNA-Seq | Exp. Atlas; Data from Merkin et al, 2012 |
| *B. taurus* | Muscle tissue | *ttyh1*  *ttyh2*  *ttyh3* | <10 TPM  30 TPM  13 TPM | RNA-Seq | Exp. Atlas; Data from Liao et al, 2014 |
| *B. taurus* | Adipose tissue | *ttyh1*  *ttyh2*  *ttyh3* | <10 TPM  <10 TPM  23 TPM | RNA-Seq | Exp. Atlas; Data from Liao et al., 2014 |
| *O. aries* | Brain | *ttyh1*  *ttyh2*  *ttyh3* | 28-61 TPM  5-31 TPM  <10 - 17 TPM | RNA-Seq | Exp. Atlas; Data from Jiang et al., 2014 |
| *O. aries* | Testis | *ttyh1*  *ttyh2*  *ttyh3* | 31 TPM  17 TPM  <10 TPM | RNA-Seq | Exp. Atlas; Data from Jiang et al., 2014 |
| *O. aries* | Uterus | *ttyh1*  *ttyh2*  *ttyh3* | <10 TPM  <10 TPM  12 TPM | RNA-Seq | Exp. Atlas; Data from Jiang et al., 2014 |
| *O. aries* | Uterine cervix | *ttyh1*  *ttyh3* | <10 TPM  11 TPM | RNA-Seq | Exp. Atlas; Data from Jiang et al., 2014 |
| *O. aries* | Mesenteric lymph node | *ttyh1*  *ttyh2*  *ttyh3* | <10 TPM  <10 TPM  12 TPM | RNA-Seq | Exp. Atlas; Data from Jiang et al., 2014 |
| *O. aries* | Thyroid gland | *ttyh1*  *ttyh2*  *ttyh3* | <10 TPM  <10 TPM  <10 TPM (male), 12 TPM (female) | RNA-Seq | Exp. Atlas; Data from Jiang et al., 2014 |
| *O. aries* | Spleen, abomasum, colon, liver, cecum, duodenum, omentum, lung, mammary gland, cortex of kidney, ovary, pvery’s patch, skin from side, inner lining mucosa of the abomasum, pre capsular lymph node, renal medulla, corpus luteum | *ttyh1*  *ttyh2*  *ttyh3* | <10 TPM | RNA-seq | Exp. Atlas; Data from Jiang et al., 2014 |
| *O. aries* | Adrenal gland, alveolar macrophage, biceps, rumen, epididymis, longissimus thoracis muscle, pituitary gland, skin of back, ventricle, thyroid gland | *ttyh1*  *ttyh3* | <10 TPM | RNA-Seq | Exp. Atlas; Data from Jiang et al., 2014 |
| *O. aries* | Tonsil, cardiac ventricle, placenta | *ttyh2*  *ttyh3* | <10 TPM | RNA-Seq | Exp. Atlas; Jiang et al., 2014 |
| *E. caballus* | Cerebellum | *ttyh1*  *ttyh2*  *ttyh3* | 175 TPM  52 TPM  37 TPM | RNA-Seq | Exp. Atlas; Data from Coleman et al., 2013 |
| *E. caballus* | Testis | *ttyh1*  *ttyh2*  *ttyh3* | 31 TPM  <10 TPM  <10 TPM | RNA-Seq | Exp. Atlas; Data from Coleman et al., 2013 |
| *E. caballus* | Placental villous | *ttyh2*  *ttyh3* | <10 TPM  18 TPM | RNA-Seq | Exp. Atlas; Data from Coleman et al., 2013 |
| *M. domestica*  (Opossum) | Central nervous system; samples from the brain (excluding cerebellum) and cerebellum | *ttyh1*  *ttyh2*  *ttyh3* | 559 TPM (brain), 463 TPM (cerebellum)    42 TPM (brain), 153 TPM (cerebellum)  77 TPM (brain), 88 TPM (cerebellum) | RNA-Seq | Exp. Atlas, 2020; Raw Data from Brawand et al., 2011 |
| *M. domestica* | Testis | *ttyh1*  *ttyh2*  *ttyh3* | <10 TPM  25 TPM  26 TPM | RNA-Seq | Exp. Atlas; Raw Data from Brawand et al., 2011 |
| *M. domestica* | Liver | *ttyh2*  *ttyh3* | <10 TPM  11 TPM | RNA-Seq | Exp. Atlas; Raw Data from Brawand et al.,2011 |
| *M. domestica* | Kidney | *ttyh2*  *ttyh3* | 24 TPM  66 TPM | RNA-Seq | Exp. Atlas; Data from Brawand et al., 2011 |
| *M. domestica* | Heart | *ttyh1*  *ttyh2*  *ttyh3* | <10 TPM | RNA-Seq | Exp. Atlas;  Data from Brawand et al., 2011 |
| *S. scrofa* | Sampled regions of the brain | *ttyh1*  *ttyh2*  *ttyh3* | 5-19 TPM  <10 - 94 TPM  <10 - 13 TPM | RNA-Seq | Exp. Atlas |
| *S. scrofa* | Spleen | *ttyh1*  *ttyh2*  *ttyh3* | <10 TPM  10 (male), 17 (female) TPM  <10 TPM (male), 10 TPM (female) | RNA-Seq | Exp. Atlas |
| *S. scrofa* | Uterus | *ttyh2*  *ttyh3* | <10 TPM | RNA-Seq | Exp. Atlas |
| *S. scrofa* | Digestive system | *ttyh1*  *ttyh2*  *ttyh3* | <10 TPM, absent from several sampled organs  <10 - 13 TPM  <10 TPM | RNA-Seq | Exp. Atlas |
| *S. scrofa* | Pituitary gland | *ttyh1*  *ttyh2*  *ttyh3* | <10 TPM | RNA-Seq | Exp. Atlas |
| *S. scrofa* | Cortex of kidney | *ttyh2*  *ttyh3* | 22 (male), 25 (female) TPM  <10 TPM | RNA-Seq | Exp. Atlas |
| *S. scrofa* | Alveolus of the lung | *ttyh1*  *ttyh2*  *ttyh3* | <10 TPM | RNA-Seq | Exp. Atlas |
| *S. scrofa* | Skeletal muscle tissue | *ttyh2*  *ttyh3* | <10 TPM | RNA-Seq | Exp. Atlas |
| *G. gallus* | Brain | *ttyh2*  *ttyh3* | 15 TPM  92 TPM | RNA-Seq | Exp. Atlas; Data from Merkin et al., 2012 |
| *G. gallus* | Brain | *ttyh2*  *ttyh3* | 17 TPM  199 TPM | RNA-Seq | Exp. Atlas; Data from Liao Barbosa-Morais et al, 2012 |
| *G. gallus* | Testis | *ttyh2*  *ttyh3* | <10 TPM  20 TPM | RNA-Seq | Exp. Atlas; Data from Merkin et al., 2012 |
| *G. gallus* | Spleen | *ttyh2*  *ttyh3* | 30 TPM  139 TPM | RNA-Seq | Exp. Atlas; Data from Merkin et al., 2012 |
| *G. gallus* | Liver | *ttyh2*  *ttyh3* | 11 TPM  <10 TPM | RNA-Seq | Exp. Atlas; Data from Merkin et al., 2012 |
| *G. gallus* | Liver | *ttyh2*  *ttyh3* | <10 TPM  <10 TPM | RNA-Seq | Exp. Atlas; Data from Barbosa-Morais et al, 2012 |
| *G. gallus* | Kidney | *ttyh2*  *ttyh3* | <10 TPM  33 TPM | RNA-Seq | Exp. Atlas; Data from Merkin et al., 2012 |
| *G. gallus* | Kidney | *ttyh2*  *ttyh3* | <10 TPM  21 TPM | RNA-Seq | Exp. Atlas; Data from Barbosa-Morais et al, 2012 |
| *G. gallus* | Lung | *ttyh2*  *ttyh3* | <10 TPM  52 TPM | RNA-Seq | Exp. Atlas; Data from Merkin et al., 2012 |
| *G. gallus* | Heart | *ttyh2*  *ttyh3* | <10 TPM  <10 TPM | RNA-Seq | Exp. Atlas; Data from Merkin et al., 2012 |
| *G. gallus* | Heart | *ttyh2*  *ttyh3* | <10 TPM  14 TPM | RNA-Seq | Exp. Atlas; Data from Barbosa-Morais et al, 2012 |
| *G. gallus* | Colon | *ttyh2*  *ttyh3* | 11 TPM  43 TPM | RNA-Seq | Exp. Atlas; Data from Merkin et al., 2012 |
| *G. gallus* | Skeletal Muscle Tissue | *ttyh3* | <10 TPM | RNA-Seq | Exp. Atlas; Data from Merkin et al., 2012 |
| *G. gallus* | Skeletal Muscle Tissue | *ttyh2*  *ttyh3* | <10 TPM  <10 TPM | RNA-Seq | Exp. Atlas; Data from Barbosa-Morais et al, 2012 |
| *A. Carolinensis* | Brain | *ttyh2* | 50 TPM | RNA-Seq | Exp. Atlas; Data from Barbosa-Morais et al., 2012 |
| *A. Carolinensis* | Heart | *ttyh2* | <10 TPM | RNA-Seq | Exp. Atlas; Data from Barbosa-Morais et al., 2012 |
| *A. Carolinensis* | Kidney | *ttyh2* | <10 TPM | RNA-Seq | Exp. Atlas; Data from Barbosa-Morais et al., 2012 |
| *X. laevis* | Brain | *ttyh1.s*  *ttyh1.l*  *ttyh2*  *ttyh3.s*  *ttyh3.l* | 174.74 TPM  108.37 TPM  13.95 TPM  41.16 TPM  55.96 TPM | RNA-Seq | Xenbase; Data from Sessions et al., 2016 |
| *X. laevis* | Eyes | *ttyh1.s*  *ttyh1.l*  *ttyh2*  *ttyh3.s*  *ttyh3.l* | 58 TPM  31.86 TPM  26.42 TPM  15.71 TPM  18.67 TPM | RNA-Seq | Xenbase; Data from Sessions et al., 2016 |
| *X. laevis* | Heart | *ttyh1.s*  *ttyh1.l*  *ttyh2*  *ttyh3.s*  *ttyh3.l* | <10 TPM  Below 0.5 TPM cutoff  <10 TPM  12 TPM  <10 TPM | RNA-Seq | Xenbase; Data from Sessions et al., 2016 |
| *X. laevis* | Intestine | *ttyh1.s*  *ttyh1.l*  *ttyh2*  *ttyh3.s*  *ttyh3.l* | <10 TPM  Below 0.5 TPM cutoff  <10TPM  <10 TPM  <10 TPM | RNA-Seq | Xenbase;  Data from Sessions et al., 2016 |
| *X. laevis* | Kidney | *ttyh1.s*  *ttyh1.l*  *ttyh2*  *ttyh3.s*  *ttyh3.l* | <10 TPM | RNA-Seq | Xenbase; Data from Sessions et al., 2016 |
| *X. laevis* | Liver | *ttyh1.s*  *ttyh1.l*  *ttyh2*  *ttyh3.s*  *ttyh3.l* | <10 TPM | RNA-Seq | Xenbase; Data from Sessions et al., 2016 |
| *X. laevis* | Lung | *ttyh1.s*  *ttyh1.l*  *ttyh2*  *ttyh3.s*  *ttyh3.l* | <10 TPM | RNA-Seq | Xenbase; Data from Sessions et al., 2016 |
| *X. laevis* | Muscle | *ttyh1.s*  *ttyh1.l*  *ttyh2*  *ttyh3.s*  *ttyh3.l* | <10 TPM  Below a 0.5 TPM cutoff  Below a 0.5 TPM cutoff  <10 TPM  <10 TPM | RNA-Seq | Xenbase; Data from Sessions et al., 2016 |
| *X. laevis* | Ovary | *ttyh1.s*  *ttyh1.l*  *ttyh2*  *ttyh3.s*  *ttyh3.l* | 0.8 TPM  Below a 0.5 TPM  cutoff  Below a 0.5 TPM cutoff  13.93 TPM  9.64 TPM | RNA-Seq | Xenbase; Data from Sessions et al., 2016 |
| *X. laevis* | Pancreas | *ttyh1.s*  *ttyh1.l*  *ttyh2*  *ttyh3.s*  *ttyh3.l* | 0.54 TPM  0.59 TPM  Below a 0.5 TPM  0.8 TPM  0.95 TPM | RNA-Seq | Xenbase; Data from Sessions et al., 2016 |
| *X. laevis* | Skin | *ttyh1.s*  *ttyh1.l*  *ttyh2*  *ttyh3.s*  *ttyh3.l* | Below a 0.5 TPM cutoff  Below a 0.5 TPM cutoff  2.19 TPM  8.52 TPM  7.04 TPM | RNA-Seq | Xenbase; Data from Sessions et al., 2016 |
| *X. laevis* | Stomach | *ttyh1.s*  *ttyh1.l*  *ttyh2*  *ttyh3.s*  *ttyh3.l* | 1.01 TPM  Below a 0.5 TPM cutoff  0.83 TPM  1.87 TPM  7.62 TPM | RNA-Seq | Xenbase; Data from Sessions et al., 2016 |
| *X. laevis* | Spleen | *ttyh1.s*  *ttyh1.l*  *ttyh2*  *ttyh3.s*  *ttyh3.l* | 4.9 TPM  Below a 0.5 TPM cutoff  5.22 TPM  36.89 TPM  49.83 TPM | RNA-Seq | Xenbase; Data from Sessions et al., 2016 |
| *X. laevis* | Testis | *ttyh1.s*  *ttyh1.l*  *ttyh2*  *ttyh3.s*  *ttyh3.l* | 1.37 TPM  3.45 TPM  4.88 TPM  7.59 TPM  17.25 TPM | RNA-Seq | Xenbase; Data from Sessions et al., 2016 |
| X. tropicalis | Brain | *ttyh1*  *ttyh2* | 149 TPM  44 TPM | RNA-Seq | Exp. Atlas; Data from Barbosa-Morais et al., 2012 |
| *X. tropicalis* | Liver | *ttyh2* | 18 TPM | RNA-Seq | Exp. Atlas; Data from Barbosa-Morais et al., 2012 |
| *X. tropicalis* | Heart | *ttyh2* | <10 TPM; | RNA-Seq | Exp. Atlas; Data from Barbosa-Morais et al., 2012 |
| *X. tropicalis* | Kidney | *ttyh1*  *ttyh2* | <10 TPM  <10 TPM | RNA-Seq | Exp. Atlas; Data from Barbosa-Morais et al., 2012 |
| *X. tropicalis* | Skeletal muscle tissue | *ttyh2* | <10 TPM | RNA-Seq | Exp. Atlas; Data from Barbosa-Morais et al., 2012 |
| *C. elegans* | Broad expression, with expression in parts of respiratory system, excretory system, digestive system, reproductive system, and muscular system | ttyh1 | N/A | GFP-  constructs | WormBase; Description of expression uses data from McKay et al, 2003 and Hunt-Newbury et al, 2007 |
| *D. melanogaster* | Heads, accessory glands, ovaries | *tty* | Values vary depending on exonic region, consult FlyBase for further information | RNA-Seq | FlyBase |
| *D. melanogaster* | Ovaries, digestive system, head, accessory organs | *CG3638* | Values vary depending on exonic region, consult FlyBase for further information | RNA-Seq | FlyBase; Data from Brown et al., 2014 and Graveley et al., 2011.4.13 |

**Supplementary Table 4.** Differential *tweety* expression comparing pathological to nonpathological cells and tissues (unless otherwise specified) in cancers and precancerous conditions. *Exp. Atlas* – Expression Atlas, Papatheodorou et al., 2020; ONCOMINE – http://oncomine.org. The paper following “Data from” provided raw data from which the proceeding database generated expression profiles/datasets. The databases list these papers as being sources for original data, so we have included them for complete transparency.

| **Pathology** | **Cell/Tissue Type** | ***tweety* Expression** | **Method** | **Source** |
| --- | --- | --- | --- | --- |
| Atypical teratoid rhabdoid tumors | Human atypical teratoid rhabdoid tumors | *TTYH2*  downregulation | Microarray | Exp. Atlas; Data from Griesinger et al., 2015 |
| Atypical teratoid rhabdoid tumors | Human atypical teratoid rhabdoid tumors | *TTYH3*  upregulation | Microarray | Exp. Atlas; Data from Birks et al., 2013 |
| Breast cancer | Human mucinous breast carcinoma samples | *TTYH1* downregulation | Microarray | ONCOMINE; Data from Curtis et al., 2012 |
| Breast cancer | Human tubular breast carcinoma samples | *TTYH1* downregulation | Microarray | ONCOMINE; Data from Curtis et al., 2012 |
| Breast cancer | Human invasive lobular breast carcinoma samples | *TTYH1* downregulation | Microarray | ONCOMINE; Data from Curtis et al., 2012 |
| Breast cancer | Human invasive ductal and invasive lobular breast carcinoma samples | *TTYH1* downregulation | Microarray | ONCOMINE; Data from Curtis et al., 2012 |
| Breast cancer | Human invasive ductal breast carcinoma samples | *TTYH1* downregulation | Microarray | ONCOMINE; Data from Curtis et al., 2012 |
| Breast cancer | Human non-triple-negative breast cancer tissue | *TTYH1* downregulation | RNA-Seq | Exp. Atlas; Data from Eswaran et al., 2012 |
| Breast cancer | Human HER2-positive breast cancer tissue | *TTYH1* downregulation | RNA-Seq | Exp. Atlas; Data from Eswaran et al., 2012 |
| Breast cancer | Human breast carcinoma tissue | *TTYH1* downregulation | Microarray | Exp. Atlas; Data from Tahiri et al., 2013 |
| Breast cancer | Human microdissected non-inflammatory breast cancer tumor | *TTYH1* downregulation | Microarray | Exp. Atlas; Data from Woodward et al., 2013 |
| Breast cancer | Human breast cancer mammary gland tumor sample | *TTYH1* downregulation | Microarray | Exp. Atlas; Data from Tan et al., 2014 |
| Breast cancer | Human blood platelets of individuals with breast cancer | *TTYH1* downregulation | RNA-Seq | Exp. Atlas; Data from Best et al., 2015 |
| Pre-malignant breast cancer | Human pure epithelial cell dissections | *TTYH1* downregulation | Microarray | Exp. Atlas; Data from Lee et al., 2007 |
| Breast cancer | Human non-triple-negative breast tumor samples | *TTYH2* downregulation | RNA-Seq | Exp. Atlas; Data from Eswaran et al., 2012 |
| Breast cancer | Human blood platelets of individuals with breast cancer | *TTYH2* downregulation | RNA-Seq | Exp. Atlas; Data from Best et al., 2015 |
| Breast cancer | Human medullary breast carcinoma samples | *TTYH3* upregulation | Microarray | ONCOMINE; Data from Curtis et al., 2012 |
| Breast cancer | Human triple-negative breast cancer mammary tissue | *TTYH3* upregulation | Microarray | Exp. Atlas; Data from Komatsu et al., 2012 |
| Breast cancer | Human blood platelets of individuals with breast cancer | *TTYH3* upregulation | RNA-Seq | Exp. Atlas; Data from Best et al., 2015 |
| Clear cell sarcoma | Mouse clear cell sarcoma model tumor induced by TAT-Cre | *ttyh1* upregulation | RNA-Seq | Exp. Atlas; Data from Straessler et al., 2013 |
| Clear cell sarcoma | Mouse clear cell sarcoma model tumor induced by *Rosa26CreER* | *ttyh1* upregulation | RNA-Seq | Exp. Atlas; Data from Straessler et al., 2013 |
| Clear cell sarcoma | Mouse clear cell sarcoma model tumor induced by TAT-Cre | *ttyh2* upregulation | RNA-Seq | Exp. Atlas; Data from Straessler et al., 2013 |
| Clear cell sarcoma | Mouse clear cell sarcoma model tumor induced by TAT-Cre | *ttyh3* upregulation | RNA-Seq | Exp. Atlas; Data from Straessler et al., 2013 |
| Clear cell sarcoma | Mouse clear cell sarcoma model tumor induced by *Rosa26CreER* | *ttyh3* upregulation | RNA-Seq | Exp. Atlas; Data from Straessler et al., 2013 |
| Colorectal cancer | Human blood platelets of individuals with colorectal cancer | *TTYH1* downregulation | RNA-Seq | Exp. Atlas; Data from Best et al., 2015 |
| Colon cancer | Human colon cancer tissue | *TTYH2* upregulation | RT-PCR | Toiyama et al., 2007 |
| Colon cancer | Caco-2, LoVo, and DLD-1 (colon cancer cell lines) | *TTYH2* upregulation | RT-PCR | Toiyama et al., 2007 |
| Colorectal cancer | Human blood platelets of individuals with colorectal cancer | *TTYH2* downregulation | RNA-Seq | Exp. Atlas; Data from Best et al. 2015 |
| Colon cancer | LoVo cells overexpressing β-COP | Decreased *TTYH2* surface expression | Immunocytochemistry | Ryu et al., 2019 |
| Colon adenoma | Human colon adenoma samples | *TTYH3* upregulation | Microarray | ONCOMINE; Data from Sabates-Bellver et al., 2007 |
| Colon adenoma | Human colon biopsy | *TTYH3* upregulation | Microarray | Exp. Atlas; Data from Galamb et al., 2007 |
| Rectal adenocarcinoma | Human rectal adenocarcinoma | *TTYH3* upregulation | Microarray | ONCOMINE; Data from Gaedcke et al., 2010 |
| Colorectal cancer | Human blood platelets of patients with colorectal cancer | *TTYH3* upregulation | RNA-Seq | Exp. Atlas; Data from Best et al., 2015 |
| Colon adenocarcinoma | Human sessile serrated colon adenoma/polyps | *TTYH3* upregulation | RNA-Seq | Exp. Atlas; Data from Kanth et al., 2016 |
| Colorectal cancer | Human primary colorectal cancer tumor | *TTYH3* upregulation | RNA-Seq | Exp. Atlas; Data from Hanley et al., 2017 |
| Esophageal squamous cell carcinoma | Human esophageal squamous cell carcinoma tumor | *TTYH1* downregulation | RNA-Seq | Exp. Atlas; Data from Tong et al., 2012 |
| Esophageal adenocarcinoma | Human esophageal adenocarcinoma tissue | *TTYH1* downregulation | RNA-Seq | Exp. Atlas; Data from Maag et al., 2017 |
| Esophageal squamous cell carcinoma | Human esophageal squamous cell carcinoma tumor | *TTYH2* upregulation | RNA-Seq | Exp. Atlas; Data from Tong et al., 2012 |
| Esophageal adenocarcinoma | Human esophageal adenocarcinoma tissue | *TTYH2* upregulation | RNA-Seq | Exp. Atlas; Data from Maag et al., 2017 |
| Esophageal adenocarcinoma | Human esophageal adenocarcinoma samples | *TTYH3* upregulation | Microarray | ONCOMINE; Data from Kim et al., 2010 |
| Esophageal squamous cell carcinoma | Human esophageal squamous cell carcinoma tumor | *TTYH3*  upregulation | RNA-Seq | Exp. Atlas; Data from Tong et al., 2012 |
| Esophageal adenocarcinoma | Human esophageal adenocarcinoma tissue | *TTYH3* upregulation | RNA-Seq | Exp. Atlas; Data from Maag et al., 2017 |
| Gastric cancer | Human gastric intestinal type adenocarcinoma samples | *TTYH3* upregulation | Microarray | ONCOMINE; Data from Chen et al., 2003 |
| Gastric cancer | Human gastric mixed adenocarcinoma | *TTYH3* upregulation | Microarray | ONCOMINE; Data from Chen et al., 2003 |
| Gastric cancer | Human gastric intestinal type adenocarcinoma samples | *TTYH3* upregulation | Microarray | ONCOMINE; Data from D’Errico et al., 2009 |
| Gastric cancer | Human gastric intestinal type adenocarcinoma samples | *TTYH3* upregulation | Microarray | ONCOMINE; Data from Cho et al., 2011 |
| Gastric cancer | Human diffuse gastric adenocarcinoma samples | *TTYH3* upregulation | Microarray | ONCOMINE; Data from Cho et al., 2011 |
| Gastric cancer | Human gastric cancer tissue | *TTYH3* upregulation associated with gastric cancer tissue | Immunocytochemistry | Saha et al., 2019 |
| Gastric cancer | Human gastric cancer tissue | *TTYH3* upregulation associated with decreased survival | Kaplan-Meier analysis | Saha et al., 2019 |
| Glioma | Human blood platelets of individuals with glioma | *TTYH1* downregulation | RNA-Seq | Exp. Atlas; Data from Best et al., 2015 |
| Glioma | Mouse glioblastoma multiforme microglial cells | *ttyh1* downregulation | Microarray | Exp. Atlas |
| Glioma | Mice brains implanted with primary culture glioblastoma cells that had been grown under non-differentiating, stem-like conditions compared to mice brains implanted with primary culture glioblastoma cells that had been grown under differentiating, serum-containing conditions | *ttyh1* downregulation | Microarray | Jung et al., 2017 |
| Glioma | Human 1p/19q non-codeleted/*IDH* mutant gliomas compared with 1p/19q codeleted/*IDH* mutant gliomas | *TTYH1* upregulation | RNA-Seq | Osswald et al., 2015 |
| Glioma | Human infiltrating compared to neoplastic cells in glioblastoma patients | *TTYH1* upregulation | Single cell RNA-Seq | Darmanis et al., 2017 |
| Glioma | Mouse brain glioma-associated macrophages | *ttyh2* upregulation | Microarray | Exp. Atlas; Data from Huang et al., 2014 |
| Glioma | Human contrast-enhancing core brain glioma sample | *TTYH2* downregulation | RNA-Seq | Exp. Atlas; Data from Gill et al., 2014 |
| Glioma | Human 1p/19q non-codeleted/*IDH* mutant gliomas compared with 1p/19q codeleted/*IDH* mutant gliomas | *TTYH2* upregulation | RNA-Seq | Osswald et al., 2015 |
| Glioma | Human grade 4 pediatric glioblastoma tumors | *TTYH3* upregulation | Microarray | Exp. Atlas; Data from Birks et al., 2013 |
| Glioma | Human contrast-enhancing core brain glioma sample | *TTYH3* upregulation | RNA-Seq | Exp. Atlas; Data from Gill et al., 2014 |
| Glioma | Human 1p/19q non-codeleted/*IDH* mutant gliomas compared with 1p/19q codeleted/*IDH* mutant gliomas | *TTYH3* downregulation | RNA-Seq | Osswald et al., 2015 |
| Hepatobiliary carcinoma | Human blood platelets of individuals with hepatobiliary carcinoma | *TTYH1* downregulation | RNA-Seq | Exp. Atlas; Data from Best et al., 2015 |
| Hepatobiliary carcinoma | Human blood platelets of individuals with hepatobiliary carcinoma | *TTYH3* upregulation | RNA-Seq | Exp. Atlas; Data from Best et al., 2015 |
| Kidney cancer | Mouse secondary tumors arising from Dox-withdrawn kidney tumor cells subcutaneously transplanted into immunocompromised mice | *ttyh1* upregulation | Microarray | Exp. Atlas; Data from Ohnishi et al., 2014 |
| Kidney cancer | Mouse secondary tumors arising from Dox-withdrawn kidney tumor cells subcutaneously transplanted into immunocompromised mice | *ttyh2* downregulation | Microarray | Exp. Atlas; Data from Ohnishi et al., 2014 |
| Kidney cancer | Mouse kidney tumor | *ttyh2* downregulation | Microarray | Exp. Atlas; Data from Ohnishi et al., 2014 |
| Kidney cancer | Mouse secondary tumors arising from Dox-withdrawn kidney tumor cells subcutaneously transplanted into immunocompromised mice | *ttyh3* upregulation | Microarray | Exp. Atlas; Data from Ohnishi et al., 2014 |
| Liver cancer | Mouse liver tumor | *ttyh3* upregulation | Microarray | Exp. Atlas; Data from Ohnishi et al., 2014 |
| Lung carcinoma | Mouse Ep-CAM positive, CD11b negative, CD11c negative epithelial cells | *ttyh1* upregulation | RNA-Seq | Exp. Atlas; Data from Choi et al., 2015 |
| Lung carcinoma | Human blood platelets of individuals with non-small cell lung carcinoma | *TTYH1* downregulation | RNA-Seq | Exp. Atlas; Data from Best et al., 2015 |
| Lung carcinoma | Mouse Ep-CAM positive, CD11b negative, CD11c negative epithelial cells | *ttyh2* upregulation | RNA-Seq | Exp. Atlas; Data from Choi et al., 2015 |
| Lung carcinoma | Mouse CD11b positive, Ly6G positive neutrophils | *ttyh2* upregulation | RNA-Seq | Exp. Atlas; Data from Choi et al., 2015 |
| Lung carcinoma | Mouse lung tissue | *ttyh2* upregulation | RNA-Seq | Exp. Atlas; Data from Choi et al., 2015 |
| Lung carcinoma | Human blood platelets of individuals with non-small cell lung carcinoma | *TTYH2* downregulation | RNA-Seq | Exp. Atlas; Data from Best et al., 2015 |
| Lung adenocarcinoma | Human lung adenocarcinoma samples | *TTYH3* upregulation | Microarray | ONCOMINE; Data from Selamat et al., 2012 |
| Lung carcinoma | Human blood platelets of individuals with non-small cell lung carcinoma | *TTYH3* upregulation | RNA-Seq | Exp. Atlas; Data from Best et al., 2015 |
| Lymphoma | Human Hodgkin’s lymphoma samples | *TTYH2* upregulation | Microarray | ONCOMINE; Data from Brune et al., 2008 |
| Lymphoma | Human follicular lymphoma samples | *TTYH2 upregulation* | Microarray | ONCOMINE; Data from Compagno et al., 2009 |
| Lymphoma | Human diffuse large B-cell lymphoma samples | *TTYH2 upregulation* | Microarray | ONCOMINE; Data from Compagno et al., 2009 |
| Medulloblastoma | Human grade 4 pediatric medulloblastoma tumors | *TTYH1* downregulation | Microarray | Exp. Atlas; Data from Birks et al., 2013 |
| Medulloblastoma | Human medulloblastoma tumors from epilepsy surgery | *TTYH1* downregulation | Microarray | Exp. Atlas; Data from Griesinger et al., 2013 |
| Medulloblastoma | Human group 3 medulloblastoma tumors | *TTYH1* downregulation | Microarray | Exp. Atlas; Data from Griesinger et al., 2015 |
| Medulloblastoma | Human group 4 medulloblastoma tumors | *TTYH1* downregulation | Microarray | Exp. Atlas; Data from Griesinger et al., 2015 |
| Sonic Hedgehog Medulloblastoma | Human Sonic Hedgehog medulloblastoma tumors | *TTYH1* downregulation | Microarray | Exp. Atlas; Data from Griesinger et al., 2015 |
| Medulloblastoma | Human grade 4 pediatric medulloblastoma tumors | *TTYH3* upregulation | Microarray | Exp. Atlas; Data from Birks et al., 2013 |
| Melanoma | Human melanoma samples | *TTYH3* upregulation | Microarray | ONCOMINE; Data from Haqq et al., 2005 |
| Myxosarcoma | Human atrial myxoma-derived cells | *TTYH3* downregulation | RNA-Seq | Exp. Atlas; Data from Scalise et al., 2020 |
| Osteosarcoma | Mouse osteosarcoma samples | *ttyh1* downregulation | RNA-Seq | Exp. Atlas; Data from Straessler et al., 2013 |
| Osteosarcoma | Mouse osteosarcoma samples | *ttyh2* upregulation | RNA-Seq | Exp. Atlas; Data from Straessler et al., 2013 |
| Osteosarcoma | U2OS and MG-63 when compared to Fob11.9 and SaOS2 (osteosarcoma-derived cell lines) | *TTYH2* upregulation | RT-PCR | Moon et al., 2019 |
| Osteosarcoma | Human bone cancer tissue | *TTYH3* upregulation | Microarray | Exp. Atlas; Data from Jones et al., 2012 |
| Osteosarcoma | Mouse osteosarcoma samples | *ttyh3* upregulation | RNA-Seq | Exp. Atlas; Data from Straessler et al., 2013 |
| Ovarian cancer | Human ovarian epithelial tissue | *TTYH2* downregulation | Microarray | Exp. Atlas; Data from Yeung et al., 2013 |
| Pancreatic adenocarcinoma | Human laser-microdissected intraductal papillary-mucinous adenoma (IPMA) pancreas epithelial tissue | *TTYH1* downregulation | Microarray | Exp. Atlas; Data from Hiraoka et al., 2010 |
| Pancreatic adenocarcinoma | Human blood platelets of individuals with pancreatic adenocarcinoma | *TTYH1* downregulation | RNA-Seq | Exp. Atlas; Data from Best et al., 2015 |
| Pancreatic adenocarcinoma | Human pancreatic adenocarcinoma samples | *TTYH2* downregulation | Microarray | ONCOMINE; Data from Logsdon et al., 2003 |
| Pancreatic adenocarcinoma | Human laser-microdissected intraductal papillary-mucinous adenoma (IPMA) pancreas epithelial tissue | *TTYH2* downregulation | Microarray | Exp. Atlas; Data from Hiraoka et al., 2010 |
| Pancreatic carcinoma | Human laser-microdissected intraductal papillary-mucinous adenoma (IPMA) pancreas epithelial tissue | *TTYH2* downregulation | Microarray | Exp. Atlas; Data from Hiraoka et al., 2010 |
| Pancreatic neoplasm | Human laser-microdissected intraductal papillary-mucinous adenoma (IPMA) pancreas epithelial tissue | *TTYH2* downregulation | Microarray | Exp. Atlas; Data from Hiraoka et al., 2010 |
| Pancreatic adenocarcinoma | Human blood platelets of individuals with pancreatic adenocarcinoma | *TTYH2* downregulation | RNA-Seq | Exp. Atlas; Data from Best et al., 2015 |
| Pancreatic cancer | Human pancreatic adenocarcinoma lesions | *TTYH3* upregulation | Microarray | Exp. Atlas; Data from Crnogorac-Jurcevic et al., 2013 |
| Renal cell carcinoma | Human renal cell carcinoma samples | *TTYH2* upregulated | DD-PCR and RT-PCR | Rae et al., 2001 |
| Renal cell carcinoma | Caki I and SN12K1 (RCC metastases-derived cell lines) | *TTYH2* expression present | RT-PCR | Rae et al., 2001 |
| Squamous cell carcinoma | Human squamous cell carcinoma skin samples | *TTYH1* downregulation | RNA-Seq | Exp. Atlas |
| Squamous cell carcinoma | Human squamous cell carcinoma skin samples | *TTYH2* upregulation | RNA-Seq | Exp. Atlas |
| Squamous cell carcinoma | Human squamous cell carcinoma skin samples | *TTYH3* upregulation | RNA-Seq | Exp. Atlas |
| Subependymal giant cell astrocytoma | Human subependymal giant cell astrocytoma with tuberous sclerosis complex samples | *TTYH1* downregulation | Microarray | Exp. Atlas; Data from Tyburczy et al., 2010 |
| Subependymal giant cell astrocytoma | Human subependymal giant cell astrocytoma with tuberous sclerosis complex samples | *TTYH2* downregulation | Microarray | Exp. Atlas; Data from Tyburczy et al., 2010 |
| Synovial sarcoma | Mouse synovial sarcoma model tumors compared with control hindlimb, intercostal, and forelimb skeletal muscle | *ttyh3* upregulation | Microarray | Exp. Atlas; Data from Haldar et al., 2007 |
| Synovial sarcoma | Mouse synovial sarcoma samples | *ttyh3* upregulation | RNA-Seq | Exp. Atlas; Data from Straessler et al., 2013 |
| Tongue squamous cell carcinoma | Human tongue squamous cell carcinoma cells | *TTYH1* upregulation | RNA-Seq | Exp. Atlas; Data from Marcinkiewicz and Gudas., 2013 |
| Uterine leiomyosarcoma | Human uterine leiomyosarcoma cells from myometrium | *TTYH3* upregulation | RNA-Seq | Exp. Atlas |

**Supplementary Table 5.** Differential *tweety* expression comparing pathological to nonpathological cells and tissues (unless otherwise specified) in neurological disorders (excluding cancers). *Exp. Atlas* – Expression Atlas, Papatheodorou et al., 2020. The paper following “Data from” provided raw data from which the proceeding database generated expression profiles/datasets. The databases list these papers as being sources for original data, so we have included them for complete transparency.

| **Pathology** | **Cell/Tissue Type** | ***tweety* Expression** | **Method** | **Source** |
| --- | --- | --- | --- | --- |
| Alzheimer’s disease | Human hippocampus samples | *TTYH1* downregulation | Microarray | Xu et al., 2006 |
| Alzheimer’s disease | Laser capture microdissection of entorhinal cortex neurons from human with Alzheimer’s disease | *TTYH1* downregulation | Microarray | Exp. Atlas; Data from Liang et al., 2007 |
| Alzheimer’s disease | Laser capture microdissection of superior frontal gyrus neurons from human with Alzheimer’s disease | *TTYH1* upregulation | Microarray | Exp. Atlas; Data from Liang et al., 2007 |
| Alzheimer’s disease | Laser capture microdissection of middle temporal gyrus neurons from human with Alzheimer’s disease | *TTYH1* upregulation | Microarray | Exp. Atlas; Data from Liang et al., 2007 |
| Alzheimer’s disease | Laser capture microdissection of middle temporal gyrus neurons of human with Alzheimer’s disease | *TTYH2* upregulation | Microarray | Exp. Atlas; Data from Liang et al., 2007 |
| Alzheimer’s disease | Laser capture microdissection of superior frontal gyrus neurons of human with Alzheimer’s disease | *TTYH2* upregulation | Microarray | Exp. Atlas; Data from Liang et al., 2007 |
| Amyotrophic lateral sclerosis | MN1 neurons expressing human TDP-43^A315T^ mutant (compared to WT) | *TTYH1* differential expression (unspecified) | Total mRNA analysis | Neelagandan et al., 2019 |
| Cerebral small vessel disease | Human brain frontal lobe cortex | *TTYH2* upregulation | Microarray | Exp. Atlas |
| Parkinson’s disease | Human locus coeruleus | *TTYH1* downregulation | Quantitative analysis of mass-spectrometry data | van Dijk et al., 2012 |
| Pick disease | Human medial temporal lobe | *TTYH2* downregulation | Microarray | Exp. Atlas; Data from Bronner et al., 2009 |
| Progressive supranuclear palsy | Human medial temporal lobe | *TTYH2* downregulation | Microarray | Exp. Atlas; Data from Bronner et al., 2009 |
| *Status epilepticus* | Rat neurons during epileptogenesis | *ttyh1* upregulation | Microarray | Lukasiuk et al., 2003; Stefaniuk and Lukasiuk, 2010 |
| *Status epilepticus* | Rat hippocampus two weeks after epileptogenic stimulus | *ttyh1* upregulation, particularly in the dentate gyrus inner molecular layer | Immunohistochemistry | Stefaniuk et al., 2010 |
| *Status epilepticus* | Reactive astrocytes in mice after amygdala-induced *Status epilepticus* | *ttyh1* upregulation | Immunohistochemistry | Wiernasz et al., 2014 |

**Supplementary Table 6.** Differential *tweety* expression comparing pathological to nonpathological cells and tissues (unless otherwise specified) in diseases other than cancers and neurological disorders. *Exp. Atlas* – Expression Atlas, Papatheodorou et al., 2020; ONCOMINE – http://oncomine.org. The paper following “Data from” provided raw data from which the proceeding database generated expression profiles/datasets. The databases list these papers as being sources for original data, so we have included them for complete transparency.

| **Pathology** | **Cell/Tissue Type** | ***tweety* Expression** | **Method** | **Source** |
| --- | --- | --- | --- | --- |
| Actinic keratosis | Human actinic keratosis skin sample | *TTYH1* downregulation | RNA-Seq | Exp. Atlas |
| Actinic keratosis | Human actinic keratosis skin sample | *TTYH2* upregulation | RNA-Seq | Exp. Atlas |
| Actinic keratosis | Human actinic keratosis skin sample | *TTYH3* upregulation | RNA-Seq | Exp. Atlas |
| Atopic dermatitis | Human atopic dermatitis skin samples | *TTYH1* upregulation | Microarray | Exp. Atlas; Data from Gittler et al., 2012 |
| Autism | Human autism samples of temporal and frontal cortex | *TTYH2* upregulation | RNA-Seq | Exp. Atlas; Data from Voineagu et al., 2011 |
| Non-dysplastic Barrett’s esophagus | Human non-dysplastic Barrett’s esophagus tissue | *TTYH2* upregulation | RNA-Seq | Exp. Atlas; Data from Maag et al., 2017 |
| Barrett’s esophagus | Human Barrett’s esophagus samples | *TTYH3* upregulation | Microarray | ONCOMINE; Data from Kim et al., 2010 |
| Non-dysplastic Barrett’s esophagus | Human non-dysplastic Barrett’s esophagus | *TTYH3* upregulation | RNA-Seq | Exp. Atlas; Data from Maag et al., 2017 |
| Chronic pancreatitis | Mouse cerulein-induced chronic pancreatitis | *ttyh1* downregulation | Microarray | Exp. Atlas; Data from Ulmasov et al., 2013 |
| Crohn’s disease | Intestinal sigmoid colon epithelial cells from children with Crohn’s disease | *TTYH3* upregulation | RNA-Seq | Exp. Atlas; Data from Howell et al., 2017 |
| Cystic fibrosis | Small and large intestines of *cftr*^tm1Cam^ mice (strong cystic fibrosis phenotype) | *ttyh3* downregulation | RT-qPCR | Braun et al., 2010 |
| Down syndrome | Human induced pluripotent stem cells from fetal fibroblast of monozygotic twins with down syndrome | *TTYH1* downregulation | RNA-Seq | Exp. Atlas; Data from Hibaoui et al., 2013 |
| Familial hemophagocytic lymphohistiocytosis | Human familial hemophagocytic lymphohistiocytosis type 5 blood mononuclear cells | *TTYH1* downregulation | RNA-Seq | Exp. Atlas |
| Influenza | Mouse lymph node B-cells | *ttyh3* downregulation | Microarray | Exp. Atlas; Data from Chang et al., 2007 |
| Juvenile idiopathic arthritis | Human autoinflammatory site-derived T cells from synovial fluid | *TTYH2* upregulation | RNA-Seq | Exp. Atlas; Data from Peeters et al., 2015 |
| Klinefelter’s syndrome | Human induced pluripotent stem cells from fibroblasts of foreskin of patient with Klinefelter’s syndrome | *TTYH1* downregulation | Microarray | Exp. Atlas; Data from Ma et al., 2012 |
| Klinefelter’s syndrome | Human induced pluripotent stem cells from fibroblasts of foreskin of patient with Klinefelter’s syndrome | *TTYH2* downregulation | Microarray | Exp. Atlas; Data from Ma et al., 2012 |
| Lyme disease | Human peripheral mononuclear blood cells during infection’s acute phase and 3 weeks after infection | *TTYH2* downregulation | RNA-Seq | Exp. Atlas |
| Metopic craniosynostosis | Human osteoblast cultures from craniosynostosis bone | *TTYH2* upregulation | RNA-Seq | Exp. Atlas; Data from Rojas-Peña et al., 2014 |
| Nevus sebaceous of Jadassohn | Human nevus sebaceous of Jadassohn skin sample | *TTYH1* downregulation | RNA-Seq | Exp. Atlas |
| Nevus sebaceous of Jadassohn | Human nevus sebaceous of Jadassohn skin sample | *TTYH2* upregulation | RNA-Seq | Exp. Atlas |
| Nevus sebaceous of Jadassohn | Human nevus sebaceous of Jadassohn skin sample | *TTYH3* upregulation | RNA-Seq | Exp. Atlas |
| Post-traumatic osteoarthritis | Synovial fluid of *Sus scrofa* at days 1, 5, 9, and 14 post-unilateral anterior cruciate ligament transections | *ttyh2* upregulation | RNA-Seq | Exp. Atlas |
| Psoriasis | Human psoriasis skin samples | *TTYH1* downregulation | Microarray | Exp. Atlas; Data from Jabbari et al., 2013 |
| Psoriasis | Human psoriasis skin samples | *TTYH1* downregulation | RNA-Seq | Exp. Atlas; Data from Li et al., 2014 |
| Pulmonary sarcoidosis | Human regulatory T cells from blood | *TTYH3* upregulation | RNA-Seq | Exp. Atlas |
| Sagittal craniosynostosis | Human osteoblast cultures from craniosynostosis bone | *TTYH2* upregulation | RNA-Seq | Exp. Atlas; Data from Rojas-Peña et al., 2014 |
| Sepsis | Human neutrophils | *TTYH2* downregulation | RNA-Seq | Exp. Atlas; Data from Linsley et al., 2014 |
| Meningococcal sepsis | Human blood and monocytes from peripheral blood | *TTYH2* downregulation | Microarray | Exp. Atlas |
| Non-primary Sjogren syndrome | Human parotid gland tissues | *TTYH2* upregulation | Microarray | Exp. Atlas; Data from Horvath et al., 2012 |
| *Streptococcus pneumoniae* (strain CGSP14) infection | Human bronchoalveolar fluid macrophages after *Streptococcus pneumoniae* infection | *TTYH2* downregulation | Microarray | Exp. Atlas |
| *Streptococcus pneumoniae* (strain CGSP14) infection and chronic obstructive pulmonary disorder | Human with chronic obstructive pulmonary disorder’s bronchoalveolar fluid macrophages after *Streptococcus pneumoniae* infection | *TTYH2* upregulation | Microarray | Exp. Atlas |
| *Streptococcus pneumoniae* (strain CGSP14) | Human bronchoalveolar fluid macrophages after *Streptococcus pneumoniae* infection | *TTYH3* downregulation | Microarray | Exp. Atlas |
| *Streptococcus pneumoniae* (strain CGSP14) infection and chronic obstructive pulmonary disorder | Human with chronic obstructive pulmonary disorder’s bronchoalveolar fluid macrophages after *Streptococcus pneumoniae* infection | *TTYH3* upregulation | Microarray | Exp. Atlas |
| Ulcerative colitis | Colon of human with ulcerative colitis | *TTYH3* upregulation | RNA-Seq | Exp. Atlas |
| Ulcerative colitis | Human intestinal epithelium | *TTYH3* upregulation | Microarray | Exp. Atlas; Data from Funke et al., 2007 |
| Ulcerative colitis | Biopsy of ascending colon and sigmoid colon of human with ulcerative colitis | *TTYH3* upregulation | RNA-Seq | Exp. Atlas; Data from Howell et al., 2017 |

**References**

Barbosa-Morais, N. L., Irimia, M., Pan, Q., Xiong, H. Y., Gueroussov, S., Lee, L. J., et al. (2012). The evolutionary landscape of alternative splicing in vertebrate species. *Science* 338, 1587–1593. doi: 10.1126/science.1230612

Best, M. G., Sol, N., Kooi, I., Tannous, J., Westerman, B. A., Rustenburg, F., et al. (2015). RNA-seq of tumor-educated platelets enables blood-based pan-cancer, multiclass, and molecular pathway cancer diagnostics. *Cancer Cell* 28, 666–676. doi: 10.1016/j.ccell.2015.09.018

Birks, D. K., Donson, A. M., Patel, P. R., Sufit, A., Algar, E. M., Dunham, C., et al. (2013). Pediatric rhabdoid tumors of kidney and brain show many differences in gene expression but share dysregulation of cell cycle and epigenetic effector genes. *Pediatr. Blood Cancer*  60, 1095–1102. doi: 10.1002/pbc.24481

Brawand, D., Soumillon, M., Necsulea, A., Julien, P., Csárdi, G., Harrigan, P., et al. (2011). The evolution of gene expression levels in mammalian organs. *Nature* 478, 343–348. doi: 10.1038/nature10532

Bronner, I. F., Bochdanovits, Z., Rizzu, P., Kamphorst, W., Ravid, R., van Swieten, J. C., et al. (2009). Comprehensive mRNA expression profiling distinguishes tauopathies and identifies shared molecular pathways. *PLoS One* 4:e6826. doi: 10.1371/journal.pone.0006826

Brown, J. B., Boley, N., Eisman, R., May, G. E., Stoiber, M. H., Duff, M. O., et al. (2014). Diversity and dynamics of the Drosophila transcriptome. *Nature* 512, 393–399. doi: 10.1038/nature12962

Brune, V., Tiacci, E., Pfeil, I., Döring, C., Eckerle, S., van Noesel, C. J., et al. (2008). Origin and pathogenesis of nodular lymphocyte-predominant Hodgkin lymphoma as revealed by global gene expression analysis. *J. Exp. Med.* 205, 2251–2268. doi: 10.1084/jem.20080809

Chang, W. L. W., Coro, E. S., Rau, F. C., Xiao, Y., Erle, D. J., and Baumgarth, N. (2007). Influenza virus infection causes global respiratory tract B cell response modulation *via* innate immune signals. *J. Immunol.* 178, 1457–1467. doi: 10.4049/jimmunol.178.3.1457

Chen, X., Leung, S. Y., Yuen, S. T., Chu, K.-M., Ji, J., Li, R., et al. (2003). Variation in gene expression patterns in human gastric cancers. *Mol. Biol. Cell* 14, 3208–3215. doi: 10.1091/mbc.e02-12-0833

Cho, J. Y., Lim, J. Y., Cheong, J. H., Park, Y.-Y., Yoon, S.-L., Kim, S. M., et al. (2011). Gene expression signature-based prognostic risk score in gastric cancer. *Clin. Cancer Res.* 17, 1850–1857. doi: 10.1158/1078-0432.CCR-10-2180

Choi, H., Sheng, J., Gao, D., Li, F., Durrans, A., Ryu, S., et al. (2015). Transcriptome analysis of individual stromal cell populations identifies stroma-tumor crosstalk in mouse lung cancer model. *Cell Rep.* 10, 1187–1201. doi: 10.1016/j.celrep.2015.01.040

Coleman, S. J., Zeng, Z., Hestand, M. S., Liu, J., and Macleod, J. N. (2013). Analysis of unannotated equine transcripts identified by mRNA sequencing. *PLoS One* 8:e70125. doi: 10.1371/journal.pone.0070125

Compagno, M., Lim, W. K., Grunn, A., Nandula, S. V., Brahmachary, M., Shen, Q., et al. (2009). Mutations of multiple genes cause deregulation of NF-kappaB in diffuse large B- cell lymphoma. *Nature* 459, 717–721. doi: 10.1038/nature 07968

Crnogorac-Jurcevic, T., Chelala, C., Barry, S., Harada, T., Bhakta, V., Lattimore, S., et al. (2013). Molecular analysis of precursor lesions in familial pancreatic cancer. *PLoS One* 8:e54830. doi: 10.1371/journal.pone.0054830

Curtis, C., Shah, S. P., Chin, S.-F., Turashvili, G., Rueda, O. M., Dunning, M. J., et al. (2012). The genomic and transcriptomic architecture of 2,000 breast tumours reveals novel subgroups. *Nature* 486, 346–352. doi: 10.1038/nature10983

D’Errico, M., de Rinaldis, E., Blasi, M. F., Viti, V., Falchetti, M., Calcagnile, A., et al. (2009). Genome-wide expression profile of sporadic gastric cancers with microsatellite instability. *Eur. J. Cancer* 45, 461–469. doi: 10.1016/j.ejca.2008. 10.032

Eswaran, J., Cyanam, D., Mudvari, P., Reddy, S. D., Pakala, S. B., Nair, S. S., et al. (2012). Transcriptomic landscape of breast cancers through mRNA sequencing. *Sci. Rep*. 2:264. doi: 10.1038/srep00264

Fagerberg, L., Hallström, B. M., Oksvold, P., Kampf, C., Djureinovic, D., Odeberg, J., et al. (2014). Analysis of the human tissue-specific expression by genome-wide integration of transcriptomics and antibody-based proteomics. *Mol. Cell Proteomics* 13, 397–406. doi: 10.1074/mcp.M113.035600

Funke, B., Lasitschka, F., Roth, W., Penzel, R., Meuer, S., Saile, M., et al. (2011). Selective downregulation of retinoic acid-inducible gene I within the intestinal epithelial compartment in Crohn’s disease. *Inflamm. Bowel Dis.* 17, 1943–1954. doi: 10.1002/ibd.21572

Gaedcke, J., Grade, M., Jung, K., Camps, J., Jo, P., Emons, G., et al. (2010). Mutated KRAS results in overexpression of DUSP4, a MAP-kinase phosphatase and SMYD3, a histone methyltransferase, in rectal carcinomas. *Genes Chromosomes Cancer* 49, 1024–1034. doi: 10.1002/gcc.20811

Galamb, O., Györffy, B., Sipos, F., Spisák, S., Németh, A. M., Miheller, P., et al. (2008). Inflammation, adenoma and cancer: objective classification of colon biopsy specimens with gene expression signature. *Dis. Markers* 25, 1–16. doi: 10.1155/2008/586721

Gill, B. J., Pisapia, D. J., Malone, H. R., Goldstein, H., Lei, L., Sonabend, A., et al. (2014). MRI- localized biopsies reveal subtype-specific differences in molecular and cellular composition at the margins of glioblastoma. *Proc. Natl. Acad. Sci.* *U S A* 111, 12550– 12555. doi: 10.1073/pnas.1405839111

Gittler, J. K., Shemer, A., Suárez-Fariñas, M., Fuentes-Duculan, J., Gulewicz, K. J., Wang, C. Q., et al. (2012). Progressive activation of T(H)2/T(H)22 cytokines and selective epidermal proteins characterizes acute and chronic atopic dermatitis. *J. Allergy Clin. Immunol.* 130, 1344–1354. doi: 10.1016/j.jaci.2012. 07.012

Graveley, B. R., Brooks, A. N., Carlson, J. W., Duff, M. O., Landolin, J. M., Yang, L., et al. (2011). The developmental transcriptome of *Drosophila melanogaster*. *Nature* 471, 473– 479. doi: 10.1038/nature09715

Griesinger, A. M., Birks, D. K., Donson, A. M., Amani, V., Hoffman, L. M., Waziri, A., et al. (2013). Characterization of distinct immunophenotypes across pediatric brain tumor types. *J. Immunol.* 191, 4880–4888. doi: 10.4049/jimmunol.1301966

Griesinger, A. M., Josephson, R. J., Donson, A. M., Mulcahy Levy, J. M., Amani, V., Birks, D. K., et al. (2015). Interleukin-6/STAT3 pathway signaling drives an inflammatory phenotype in group a ependymoma. *Cancer Immunol. Res.* 3, 1165–1174. doi: 10.1158/2326-6066.CIR-15-0061

Haldar, M., Hancock, J. D., Coffin, C. M., Lessnick, S. L., and Capecchi, M. R. (2007). A conditional mouse model of synovial sarcoma: insights into a myogenic origin. *Cancer* *Cell* 11, 375–388. doi: 10.1016/j.ccr.2007.01.016

Hanley, M. P., Hahn, M. A., Li, A. X., Wu, X., Lin, J., Wang, J., et al. (2017). Genome-wide DNA methylation profiling reveals cancer-associated changes within early colonic neoplasia. *Oncogene* 36, 5035–5044. doi: 10.1038/onc. 2017.130

Haqq, C., Nosrati, M., Sudilovsky, D., Crothers, J., Khodabakhsh, D., Pulliam, B. L., et al. (2005). The gene expression signatures of melanoma progression. *Proc. Natl. Acad. Sci. U S A* 102, 6092–6097. doi: 10.1073/pnas.0501564102

Hibaoui, Y., Grad, I., Letourneau, A., Sailani, M. R., Dahoun, S., Santoni, F. A., et al. (2014). Modelling and rescuing neurodevelopmental defect of Down syndrome using induced pluripotent stem cells from monozygotic twins discordant for trisomy 21. *EMBO Mol. Med*. 6, 259–277. doi: 10.1002/emmm.2013 02848

Hiraoka, N., Yamazaki-Itoh, R., Ino, Y., Mizuguchi, Y., Yamada, T., Hirohashi, S., et al. (2011). CXCL17 and ICAM2 are associated with a potential anti-tumor immune response in early intraepithelial stages of human pancreatic carcinogenesis. *Gastroenterology* 140, 310–321. doi: 10.1053/j.gastro.2010.10. 009

Horvath, S., Nazmul-Hossain, A. N., Pollard, R. P., Kroese, F. G., Vissink, A., Kallenberg, C. G., et al. (2012). Systems analysis of primary Sjögren’s syndrome pathogenesis in salivary glands identifies shared pathways in human and a mouse model. *Arthritis Res. Ther.* 14:R238. doi: 10.1186/ar4081

Howell, K. J., Kraiczy, J., Nayak, K. M., Gasparetto, M., Ross, A., Lee, C., et al. (2018). DNA methylation and transcription patterns in intestinal epithelial cells from pediatric patients with inflammatory bowel diseases differentiate disease subtypes and associate with outcome. *Gastroenterology* 154, 585–598. doi: 10.1053/j.gastro.2017.10.007

Huang, Y., Kim, J. K., Do, D. V., Lee, C., Penfold, C. A., Zylicz, J. J., et al. (2017). Stella modulates transcriptional and endogenous retrovirus programs during maternal-to- zygotic transition. *Elife* 6:e22345. doi: 10.7554/eLife.22345

Hunt-Newbury, R., Viveiros, R., Johnsen, R., Mah, A., Anastas, D., Fang, L., et al. (2007). High- throughput *in vivo* analysis of gene expression in Caenorhabditis elegans. *PLoS Biol.* 5:e237. doi: 10.1371/journal.pbio.0050237

Huttlin, E. L., Jedrychowski, M. P., Elias, J. E., Goswami, T., Rad, R., Beausoleil, S. A., et al. (2010). A tissue-specific atlas of mouse protein phosphorylation and expression. *Cell* 143, 1174–1189. doi: 10.1016/j.cell.2010. 12.001

Jabbari, A., Suárez-Fariñas, M., Fuentes-Duculan, J., Gonzalez, J., Cueto, I., Franks, A. G., et al. (2014). Dominant Th1 and minimal Th17 skewing in discoid lupus revealed by transcriptomic comparison with psoriasis. *J. Invest. Dermatol.* 134, 87–95. doi: 10.1038/jid.2013.269

Jiang, Y., Xie, M., Chen, W., Talbot, R., Maddox, J. F., Faraut, T., et al. (2014). The sheep genome illuminates biology of the rumen and lipid metabolism. *Science* 344, 1168–1173. doi: 10.1126/science.1252806

Jones, K. B., Salah, Z., Del Mare, S., Galasso, M., Gaudio, E., Nuovo, G. J., et al. (2012). miRNA signatures associate with pathogenesis and progression of osteosarcoma. *Cancer Res.* 72, 1865–1877. doi: 10.1158/0008-5472.CAN-11- 2663

Kanth, P., Bronner, M. P., Boucher, K. M., Burt, R. W., Neklason, D. W., Hagedorn, C. H., et al. (2016). Gene signature in sessile serrated polyps identifies colon cancer subtype. *Cancer Prev. Res.* 9, 456–465. doi: 10.1158/1940-6207.CAPR-15-0363

Keane, T. M., Goodstadt, L., Danecek, P., White, M. A., Wong, K., Yalcin, B., et al. (2011). Mouse genomic variation and its effect on phenotypes and gene regulation. *Nature* 477, 289–294. doi: 10.1038/nature10413

Kim, S. M., Park, Y.-Y., Park, E. S., Cho, J. Y., Izzo, J. G., Zhang, D., et al. (2010). Prognostic biomarkers for esophageal adenocarcinoma identified by analysis of tumor transcriptome. *PLoS One* 5:e15074. doi: 10.1371/journal.pone.0015074

Komatsu, M., Yoshimaru, T., Matsuo, T., Kiyotani, K., Miyoshi, Y., Tanahashi, T., et al. (2013). Molecular features of triple negative breast cancer cells by genome-wide gene expression profiling analysis. *Int. J. Oncol.* 42, 478–506. doi: 10.3892/ijo.2012.1744

Lee, S., Medina, D., Tsimelzon, A., Mohsin, S. K., Mao, S., Wu, Y., et al. (2007). Alterations of gene expression in the development of early hyperplastic precursors of breast cancer. *Am. J. Pathol.* 171, 252–262. doi: 10.2353/ajpath. 2007.061010

Li, B., Tsoi, L. C., Swindell, W. R., Gudjonsson, J. E., Tejasvi, T., Johnston, A., et al. (2014). Transcriptome analysis of psoriasis in a large case-control sample: RNA-seq provides insights into disease mechanisms. *J. Invest. Dermatol.* 134, 1828–1838. doi: 10.1038/jid.2014.28

Liao, X., Bao, H., Meng, Y., Plastow, G., Moore, S., and Stothard, P. (2014). Sequence, structural and expression divergence of duplicate genes in the bovine genome. *PLoS One* 9:e102868. doi: 10.1371/journal.pone.0102868

Linsley, P. S., Speake, C., Whalen, E., and Chaussabel, D. (2014). Copy number loss of the interferon gene cluster in melanomas is linked to reduced T cell infiltrate and poor patient prognosis. *PLoS One* 9:e109760. doi: 10.1371/journal.pone.0109760

Logsdon, C. D., Simeone, D. M., Binkley, C., Arumugam, T., Greenson, J. K., Giordano, T. J., et al. (2003). Molecular profiling of pancreatic adenocarcinoma and chronic pancreatitis identifies multiple genes differentially regulated in pancreatic cancer. *Cancer Res.* 63, 2649–2657.

Ma, Y., Li, C., Gu, J., Tang, F., Li, P., Ping, P., et al. (2012). Aberrant gene expression profiles in pluripotent stem cells induced from fibroblasts of a Klinefelter syndrome patient. *J. Biol. Chem.* 287, 38970–38979. doi: 10.1074/jbc.M112. 380204

Maag, J. L. V., Fisher, O. M., Levert-Mignon, A., Kaczorowski, D. C., Thomas, M. L., Hussey, D. J., et al. (2017). Novel aberrations uncovered in barrett’s esophagus and esophageal adenocarcinoma using whole transcriptome sequencing. *Mol. Cancer Res.* 15, 1558– 1569. doi: 10.1158/1541- 7786.MCR-17-0332

Marcinkiewicz, K. M., and Gudas, L. J. (2014). Altered epigenetic regulation of homeobox genes in human oral squamous cell carcinoma cells. *Exp. Cell Res.* 320, 128–143. doi: 10.1016/j.yexcr.2013.09.011

McKay, S. J., Johnsen, R., Khattra, J., Asano, J., Baillie, D. L., Chan, S., et al. (2003). Gene expression profiling of cells, tissues and developmental stages of the nematode *C. elegans. Cold Spring Harb. Symp. Quant. Biol.* 68, 159–169. doi: 10.1101/sqb.2003.68.159

Merkin, J., Russell, C., Chen, P., and Burge, C. B. (2012). Evolutionary dynamics of gene and isoform regulation in Mammalian tissues. *Science* 338, 1593–1599. doi: 10.1126/science.1228186

Naqvi, S., Godfrey, A. K., Hughes, J. F., Goodheart, M. L., Mitchell, R. N., and Page, D. C. (2019). Conservation, acquisition, and functional impact of sex-biased gene expression in mammals. *Science* 365:eaaw7317. doi: 10.1126/science.aaw7317

Ohnishi, K., Semi, K., Yamamoto, T., Shimizu, M., Tanaka, A., Mitsunaga, K., et al. (2014). Premature termination of reprogramming *in vivo* leads to cancer development through altered epigenetic regulation. *Cell* 156, 663–677. doi: 10.1016/j.cell.2014.01.005

Owens, N. D. L., Blitz, I. L., Lane, M. A., Patrushev, I., Overton, J. D., Gilchrist, M. J., et al. (2016). Measuring absolute RNA copy numbers at high temporal resolution reveals transcriptome kinetics in development. *Cell Rep.* 14, 632–647. doi: 10.1016/j.celrep.2015.12.050

Peeters, J. G., Vervoort, S. J., Tan, S. C., Mijnheer, G., de Roock, S., Vastert, S. J., et al. (2015). Inhibition of super-enhancer activity in autoinflammatory site-derived T cells reduces disease-associated gene expression. *Cell Rep.* 12, 1986–1996. doi: 10.1016/j.celrep.2015.08.046

Pfeiffer, M. J., Taher, L., Drexler, H., Suzuki, Y., Makaowski, W., Schwarzer, C., et al. (2015). Differences in embryo quality are associated with differences in oocyte composition: a proteomic study in inbred mice. *Proteomics* 15, 675–687. doi: 10.1002/pmic.201400334

Pipes, L., Li, S., Bozinoski, M., Palermo, R., Peng, X., Blood, P., et al. (2013). The non- human primate reference transcriptome resource (NHPRTR) for comparative functional genomics. *Nucleic Acids Res.* 41, D906–D914. doi: 10.1093/nar/gks1268

Rojas-Peña, M. L., Olivares-Navarrete, R., Hyzy, S., Arafat, D., Schwartz, Z., Boyan, B. D., et al. (2014). Characterization of distinct classes of differential gene expression in osteoblast cultures from non-syndromic craniosynostosis bone. *J. Genomics* 2, 121–130. doi: 10.7150/jgen.8833

Sabates-Bellver, J., Van der Flier, L. G., de Palo, M., Cattaneo, E., Maake, C., Rehrauer, H., et al. (2007). Transcriptome profile of human colorectal adenomas. *Mol. Cancer Res.* 5, 1263–1275. doi: 10.1158/1541-7786.MCR-07-0267

Scalise, M., Torella, M., Marino, F., Ravo, M., Giurato, G., Vicinanza, C., et al. (2020). Atrial myxomas arise from multipotent cardiac stem cells. *Eur. Heart J.* 41, 4332–4345. doi: 10.1093/eurheartj/ehaa156

Selamat, S. A., Chung, B. S., Girard, L., Zhang, W., Zhang, Y., Campan, M., et al. (2012). Genome-scale analysis of DNA methylation in lung adenocarcinoma and integration with mRNA expression. *Genome Res.* 22, 1197–1211. doi: 10.1101/gr.132662.111

Session, A. M., Uno, Y., Kwon, T., Chapman, J. A., Toyoda, A., Takahashi, S., et al. (2016). Genome evolution in the allotetraploid frog Xenopus laevis. *Nature* 538, 336–343. doi: 10.1038/nature19840

Straessler, K. M., Jones, K. B., Hu, H., Jin, H., van de Rijn, M., and Capecchi, M. R. (2013). Modeling clear cell sarcomagenesis in the mouse: cell of origin differentiation state impacts tumor characteristics. *Cancer Cell* 23, 215–227. doi: 10.1016/j.ccr.2012.12.019

Szabo, L., Morey, R., Palpant, N. J., Wang, P. L., Afari, N., Jiang, C., et al. (2015). Statistically based splicing detection reveals neural enrichment and tissue-specific induction of circular RNA during human fetal development. *Genome Biol.* 16:126. doi: 10.1186/s13059-015-0690-5

Tahiri, A., Leivonen, S. K., Lüders, T., Steinfeld, I., Ragle Aure, M., Geisler, J., et al. (2014). Deregulation of cancer-related miRNAs is a common event in both benign and malignant human breast tumors. *Carcinogenesis* 35, 76–85. doi: 10.1093/carcin/bgt333

Tan, T. Z., Miow, Q. H., Miki, Y., Noda, T., Mori, S., Huang, R. Y., et al. (2014). Epithelial- mesenchymal transition spectrum quantification and its efficacy in deciphering survival and drug responses of cancer patients. *EMBO Mol. Med.* 6, 1279–1293. doi: 10.15252/emmm.201404208

Tong, M., Chan, K. W., Bao, J. Y., Wong, K. Y., Chen, J. N., Kwan, P. S., et al. (2012). Rab25 is a tumor suppressor gene with antiangiogenic and anti-invasive activities in esophageal squamous cell carcinoma. *Cancer Res.* 72, 6024–6035. doi: 10.1158/0008-5472.CAN-12- 1269

Tyburczy, M. E., Kotulska, K., Pokarowski, P., Mieczkowski, J., Kucharska, J., Grajkowska, W., et al. (2010). Novel proteins regulated by mTOR in subependymal giant cell astrocytomas of patients with tuberous sclerosis complex and new therapeutic implications. *Am. J. Pathol.* 176, 1878–1890. doi: 10.2353/ajpath.2010.090950

Ulmasov, B., Oshima, K., Rodriguez, M. G., Cox, R. D., and Neuschwander-Tetri, B. A. (2013). Differences in the degree of cerulein-induced chronic pancreatitis in C57BL/6 mouse substrains lead to new insights in identification of potential risk factors in the development of chronic pancreatitis. *Am. J. Pathol.* 183, 692–708. doi: 10.1016/j.ajpath.2013. 05.020

Visel, A., Thaller, C., and Eichele, G. (2004). GenePaint.org: an atlas of gene expression patterns in the mouse embryo. *Nucleic Acids Res* 32, D552-6. doi:10.1093/nar/gkh029.

Voineagu, I., Wang, X., Johnston, P., Lowe, J. K., Tian, Y., Horvath, S., et al. (2011). Transcriptomic analysis of autistic brain reveals convergent molecular pathology. *Nature* 474, 380–384. doi: 10.1038/nature10110

Wang, D., Eraslan, B., Wieland, T., Hallström, B., Hopf, T., Zolg, D. P., et al. (2019). A deep proteome and transcriptome abundance atlas of 29 healthy human tissues. *Mol. Syst. Biol.* 15:e8503. doi: 10.15252/msb.20188503

Woodward, W. A., Krishnamurthy, S., Yamauchi, H., El-Zein, R., Ogura, D., Kitadai, E., et al. (2013). Genomic and expression analysis of microdissected inflammatory breast cancer. *Breast Cancer Res. Treat.* 138, 761–772. doi: 10.1007/s10549-013-2501-6

Yeung, T.-L., Leung, C. S., Wong, K.-K., Samimi, G., Thompson, M. S., Liu, J., et al. (2013). TGF-β modulates ovarian cancer invasion by upregulating CAF-derived versican in the tumor microenvironment. *Cancer Res.* 73, 5016–5028. doi: 10.1158/0008-5472.CAN-13- 0023

Yu, Y., Fuscoe, J. C., Zhao, C., Guo, C., Jia, M., Qing, T., et al. (2014). A rat RNA-Seq transcriptomic BodyMap across 11 organs and 4 developmental stages. *Nat. Commun.* 5:3230. doi: 10.1038/ncomms4230

Yue, F., Cheng, Y., Breschi, A., Vierstra, J., Wu, W., Ryba, T., et al. (2014). A comparative encyclopedia of DNA elements in the mouse genome. *Nature* 515, 355–364. doi: 10.1038/nature13992
